# Comparative genomics reveals synteny-guided remodeling of carbohydrate-foraging and intrinsic resistance in an ocular *Chryseobacterium* isolate, NG-CE01

**DOI:** 10.1186/s12866-026-04950-8

**Published:** 2026-03-31

**Authors:** Farouk Hassan, Süheyla Türkyılmaz, Taghrid S. El-Mahdy

**Affiliations:** 1 Department of Microbiology and Immunology, Faculty of Pharmacy, Modern University for Technology and Information, Cairo, Egypt; 2 Department of Microbiology, Faculty of Veterinary Medicine, Aydın Adnan Menderes University, Aydın, Türkiye; 3 Department of Microbiology and Immunology, Faculty of Pharmacy, Capital (formerly Helwan) University, Cairo, Egypt

**Keywords:** Chryseobacterium, Bovine keratitis, Ocular isolate, Synteny, Comparative genomics, Intrinsic resistance

## Abstract

**Background:**

*Chryseobacterium* spp. are environmentally widespread bacteria increasingly recognized as opportunistic pathogens in human and veterinary medicine, yet genome-scale determinants that may support persistence in host-associated microenvironments remain poorly defined. Isolates from anatomically specialized sites such as the ocular surface are underrepresented in comparative genomic datasets.

**Methods:**

We performed integrated phenotypic and genome-resolved characterization of *Chryseobacterium* sp. NG-CE01, isolated from a bovine corneal infection. A high-quality hybrid assembly was analyzed using phylogenomics, curated comparative gene-content profiling across a 14-genome *Chryseobacterium* panel, CAZyme annotation, synteny-guided locus interrogation, mobilome profiling, and systematic screening of antimicrobial resistance, virulence, and genome-defense determinants, supported by functional curation of isolate-specific genes.

**Results:**

Genome-based taxonomy placed NG-CE01 as a genomically distinct lineage most closely related to *Chryseobacterium cheonjiense*, falling below established species delineation thresholds and thus consistent with a putative novel genomospecies within the genus. Comparative gene-content profiling across the multi-species panel yielded an open interspecies repertoire (Heaps’ law α = 0.6897), with NG-CE01 contributing a focused isolate-specific set enriched in defense- and recombination-associated functions. Across analyses, the dominant axis of differentiation was synteny-guided, locus-scale remodeling of discrete carbohydrate-foraging modules (CBM48–GH13, CBM6–GH5, and a GH43-rich super-block), supported by conserved neighborhood architecture, Jaccard similarity gradients, and locus-centric enrichment patterns. Three genomic islands were identified; GI_3 represented the principal reservoir of isolate-specific functional novelty, enriched in envelope biogenesis and regulatory functions and consistent with a restrained mobilome. Comprehensive screening detected no acquired antimicrobial-resistance or virulence genes; instead, NG-CE01 encoded chromosomal intrinsic resistance and fitness-associated loci, while disk-diffusion results are reported as zone diameters given limited validated breakpoints for *Chryseobacterium*. Collectively, these results support a hypothesis that ocular-associated persistence may be shaped primarily by genome architecture and synteny-defined locus innovation rather than CAZyme burden or virulence acquisition.

**Conclusion:**

NG-CE01 exhibits pronounced synteny-defined remodeling of carbohydrate-foraging and intrinsic resistance loci, providing a genome-informed, hypothesis-generating framework for how *Chryseobacterium* may persist in specialized host environments and illustrating the value of synteny-aware comparative genomics for resolving functional remodeling beyond gene abundance alone.

**Supplementary Information:**

The online version contains supplementary material available at 10.1186/s12866-026-04950-8.

## Background

Members of the genus *Chryseobacterium* are environmentally widespread bacteria within the family Weeksellaceae, commonly detected in soil, water, food-processing environments, and animal-associated niches. In recent years, *Chryseobacterium* spp. have drawn increasing attention as opportunistic pathogens in both human and veterinary medicine; causing respiratory, bloodstream, wound, and ocular infections, with ocular cases reported particularly in livestock and companion animals [1–3]. Despite this clinical relevance, many isolates remain genomically and functionally under characterized, especially those recovered from anatomically specialized environments where selective pressures differ markedly from terrestrial or aquatic reservoirs. The ocular surface represents a highly restrictive ecological niche shaped by continuous tear turnover, antimicrobial peptides, immune surveillance, and a glycoprotein-rich mucosal layer dominated by host-derived carbohydrates. Persistence under these constraints is therefore more likely to rely on metabolic flexibility, adhesion, and stress tolerance than on classical virulence determinants [4–6]. Yet, for environmental-opportunistic taxa such as *Chryseobacterium*, the genomic basis of ocular niche adaptation remains poorly resolved. Most available genomic studies in this genus emphasize taxonomic assignment or resistance profiling, while offering limited locus-level resolution and little attention to niche-linked functional remodeling. Recent advances in long-read sequencing and hybrid genome assembly now enable near-complete bacterial genome reconstruction, supporting detailed interrogation of genome architecture, accessory gene dynamics, and locus-scale innovation [7, 8]. In parallel, pangenome studies indicate that many environmental bacteria maintain highly open gene repertoires shaped by ongoing gene gain, loss, and rearrangement [9]. Increasingly, comparative analyses show that ecological adaptation is often encoded not only in gene content but also in the organization and synteny of functional neighborhoods. In particular, the distribution and architecture of carbohydrate-active enzyme (CAZyme) loci can carry stronger ecological and evolutionary signals than CAZyme counts alone [10–12]. Nevertheless, synteny-aware approaches remain underutilized in studies of *Chryseobacterium* and related genera. Here, we characterize *Chryseobacterium* sp. NG-CE01, isolated from a bovine corneal infection, using an integrated phenotypic and genome-resolved framework. Leveraging a high-quality hybrid assembly, comparative phylogenomics, pangenome reconstruction, and synteny-guided locus analysis, we evaluate how carbohydrate-foraging systems, intrinsic resistance traits, and genome-defense features are organized and diversified within the *Chryseobacterium* lineage. By prioritizing locus-scale remodeling rather than bulk gene abundance, this study provides mechanistic insight into genome plasticity and niche adaptation in an opportunistic ocular isolate and offers a refined framework for interpreting functional innovation in environmentally derived pathogens.

## Materials and methods

### Study design and ethics statement

*Chryseobacterium* sp. NG-CE01 was isolated from a clinical corneal swab collected during routine veterinary diagnostic evaluation of bovine keratitis in Türkiye, following standard ocular surface sampling procedures [13]. The swab was submitted to the Microbiology Department Laboratory, Faculty of Veterinary Medicine, Aydın Adnan Menderes University for routine diagnostics. No experimental animals were used and no additional sampling was performed for research. In accordance with Article 8(o) of the ADU-HADYEK Directive, diagnostic/therapeutic clinical procedures are not subject to ethical approval; therefore, ADU-HADYEK confirmed that ethics approval was not required for this study.

### Strain isolation and phenotypic characterization

The corneal swab was cultured on Columbia blood agar (Oxoid) and incubated aerobically at 37 °C for 48 h. A yellow-pigmented colony was purified by repeated subculture and preserved at − 80 °C in Trypticase Soy Broth supplemented with 20% (v/v) glycerol (sigma aldrich, Germany). Phenotypic characterization included Gram staining and catalase, oxidase, coagulase, and urease tests, together with assays for indole production, bile esculin hydrolysis, nitrate reduction, and carbohydrate utilization. Growth and pigmentation were evaluated on MacConkey, eosin methylene blue (EMB), and triple sugar iron agars (TSI).

### Antimicrobial susceptibility testing

Antimicrobial susceptibility was assessed by Kirby–Bauer disk diffusion (Bioanalyse, Türkiye ) on Mueller–Hinton agar (Oxoid, United States). Inocula were standardized to 0.5 McFarland from 18 to 24 h cultures and incubated aerobically at 37 °C for 18–24 h. Inhibition zones were measured in millimeters and reported as mean values of replicate measurements. Because validated Clinical and Laboratory Standards Institute/ European Committee on Antimicrobial Susceptibility Testing (CLSI/EUCAST) breakpoints are unavailable for *Chryseobacterium* spp., results are presented as zone diameters with cautious interpretation (Tentative Breakpoints).

### High-molecular-weight DNA extraction

High-molecular-weight genomic DNA was extracted from overnight culture using the MagAttract HMW DNA Kit (Qiagen, Germany) using lysozyme-assisted lysis (20 mg/mL, 37 °C, 90 min). DNA was quantified using Qubit™ (Thermo Fisher Scientific, USA ) dsDNA HS assays, purity was assessed by spectrophotometry, and evaluated for integrity by 0.6% agarose gel electrophoresis.

### Hybrid sequencing and genome assembly

Hybrid whole-genome sequencing was performed using Oxford Nanopore Technologies long reads and Illumina NovaSeq paired-end sequencing (2 × 150 bp). Provider-side preprocessing included ONT read filtering with Filtlong [14], Illumina trimming with fastp [15], down sampling with Seqtk [16], read quality assessment with NanoPlot [17], and QC aggregation with MultiQC [18]. Long-read assembly was performed using Flye [19], followed by polishing with Medaka [20] and short-read polishing using BWA [21] and Polypolish [22]. The assembly was initially annotated with Bakta [23] and subsequently re-annotated with Prokka to ensure a consistent framework across comparative, pangenome, and locus-scale analyses. This Prokka annotation set was used for all downstream analyses. Initial quality assessment was performed using QUAST [24] and CheckM2 [25] (Supplementary Table S1).

### Independent quality control and genome validation

Assembly quality was independently re-assessed using Quality Assessment Tool for Genome Assemblies) (QUAST) [24] and read-level sequencing metrics were evaluated using FastQC and MultiQC [18]. Taxonomic purity was screened using Kraken2, and genome completeness was evaluated using Benchmarking Universal Single-Copy Orthologs (BUSCO) (bacteria_odb10).

### Taxonomic assignment and phylogenomics

Genome-based taxonomic classification was performed using GTDB-Tk v2.3.2 with the GTDB R220 database [26]. Phylogenomic inference was conducted using IQ-TREE v2.3.5 with 1,000 ultrafast bootstrap replicates. Average nucleotide identity (ANI) was calculated using FastANI, digital DNA–DNA hybridization (dDDH) was obtained via the TYGS web server, and 16 S rRNA gene similarity was assessed using BLASTn (BLAST+ v2.17.0).

### Genome annotation and functional classification

Genome annotation was generated using Prokka v1.5.2. Functional classification and COG assignment were performed using eggNOG-mapper v2.1.8 via the Galaxy platform with the eggNOG v5.0.2 database [27]. COG profiles were used for genome-wide functional summaries and enrichment analyses. COG letter definitions are provided in Supplementary Table S2A.

### Comparative gene-content analysis and gene–trait association

Gene-content diversity was analyzed across a curated 14-genome *Chryseobacterium* comparator panel (NG-CE01 plus 13 reference genomes) using Panaroo v1.5.2 [28]. Because the panel spans multiple named *Chryseobacterium* species, outputs are reported as an interspecies gene-cluster (comparative gene-content) survey rather than a species-level pangenome estimate for NG-CE01. To minimize annotation-driven bias, all genomes were annotated uniformly with Prokka and Prokka GFF3 files were used as Panaroo input. Panaroo was run with --clean-mode sensitive and --core_threshold 0.99 (core defined as present in 99–100% of genomes) to generate gene clusters and the gene presence/absence matrix; gene-repertoire growth (“openness”) was summarized descriptively using 500 random genome-order permutations and Heaps’ law modeling. Robustness was assessed in two ways: (i) parameter sensitivity by recalculating core size under alternative core-threshold settings (0.99 vs. 0.95) from the presence/absence matrix, and (ii) panel-composition sensitivity by repeating Panaroo on an ANI-selected closest-only subset. Gene–trait association analysis was performed using Scoary v1.6.16 with Benjamini–Hochberg false discovery rate correction [29].

### CAZyme identification

CAZyme annotation was performed using dbCAN3 [30], integrating HMMER and DIAMOND. Only CAZyme predictions supported by at least two independent methods were retained. CAZyme class abbreviations and biological meanings are defined in Supplementary Table S2B.

### Mobilome and genomic island analysis

Genomic islands and mobile genetic elements were identified using IslandViewer4 [31], integrating IslandPick, IslandPath-DIMOB, and SIGI-HMM. Additional mobile element screening used ISfinder, ICEfinder (ICEberg 3.0), and PHASTER [32]. Plasmid content was assessed using PlasmidFinder [33], and integrons were detected using IntegronFinder [34].

### Resistance, virulence, and genome defense profiling

Acquired antimicrobial resistance genes were screened using AMRFinderPlus v4.0.23 and ABRicate v1.0.1 against the NCBI AMR, ResFinder, and CARD databases. Virulence-associated genes were screened using ABRicate against the VFDB database (last update: 25-01-2026). CRISPR–Cas systems were detected using MinCED (Galaxy v0.1.5) and independently validated using the CRISPR Recognition Tool (CRT; Galaxy v1.2.0) [35].

### Statistical analysis and visualization

All analyses were performed on Ubuntu Linux 22.04.5 LTS. General analyses used Python v3.10.0, while dbCAN analyses used Python v3.8.20 due to dependency constraints. Synteny comparisons were visualized using clinker/clustermap.js [36], and Jaccard similarity was calculated from RBH-based gene-content overlap within the extracted windows. Circular genome and locus-scale visualizations were generated using CGView within Proksee [37], with additional figures produced using Python-based visualization libraries (Supplementary Table S1).

## Results

### Phenotypic characterization, microscopy, and antimicrobial susceptibility

Microscopy showed pleomorphic Gram-negative rods (Supplementary Fig. S1). NG-CE01 was oxidase- and catalase-positive and negative for coagulase, urease, and motility. Biochemical assays were positive for indole production, bile esculin hydrolysis, and nitrate reduction. The isolate grew on MacConkey and EMB with a non-lactose-fermenting phenotype and showed no growth on mannitol salt agar. Colonies were yellow on Mueller–Hinton and trypticase soy agar, and hemolysis was observed on blood agar. Carbohydrate utilization supported a non-fermentative profile with oxidative glucose use and a K/K reaction on TSI agar (Supplementary Table S3). Disk diffusion testing yielded measurable inhibition zones for imipenem and meropenem; ciprofloxacin, levofloxacin, and enrofloxacin; chloramphenicol and florfenicol; trimethoprim–sulfamethoxazole; gentamicin; and selected cephalosporins (cefoxitin, cefotaxime, ceftiofur) (Supplementary Table S4). No inhibition zones were observed for ampicillin, aztreonam, polymyxin B, ertapenem, tobramycin, neomycin, or bacitracin, while oxytetracycline produced a smaller inhibition zone relative to several other agents. Because species-specific CLSI/EUCAST disk-diffusion breakpoints are limited/absent for *Chryseobacterium* spp., results are reported primarily as inhibition-zone diameters (mm) and should be interpreted cautiously; raw zone values are provided in Supplementary Table S4.

### Genome sequencing, assembly, and quality assessment

De novo assembly of *Chryseobacterium* sp. NG-CE01 yielded a 4,331,766 bp genome with GC content of 37.43% (Table 1). Using a ≥ 500 bp reporting threshold, the assembly was fully contiguous, consisting of a single contig (N50 = 4,331,766 bp; L50 = 1) with 0% gaps (Table 1; Supplementary Fig. S2). This continuity was supported by the Nx curve (Supplementary Fig. S3) and a cumulative contig-length plot reaching the full genome length in one contig (Supplementary Fig. S4). Sliding-window GC content showed a unimodal distribution centered at 37.43%, consistent with compositional uniformity and arguing against large-scale contamination (Supplementary Fig. S5). BUSCO analysis (bacteria_odb10, *n* = 124) recovered 99.2% complete markers (99.2% single-copy; 0.0% duplicated), with 0.8% fragmented and none missing (Table 1). Annotation identified 3,923 protein-coding sequences, 9 rRNA genes, 63 tRNA genes, and 1 tmRNA gene (Table 1). Tandem Repeat Finder detected 198 repeats totaling 40,155 bp (0.927% of the genome) (Table 1). Collectively, these metrics indicate a high-quality, near-complete genome suitable for comparative genomics and genome-based taxonomic inference.

**Table 1:** Genome assembly and annotation statistics of *Chryseobacterium* sp. NG-CE01

Feature	Value
Genome size (bp)	4,331,766
GC content (%)	37.43
Number of contigs (≥ 500 bp)	1
Largest contig (bp)	4,331,766
N50 (bp)	4,331,766
L50	1
Percent gaps (%)	0
Protein-coding sequences (CDS)	3,923
rRNA genes	9
tRNA genes	63
tmRNA genes	1
TRF repeats (count)	198
Total repeat length (bp)	40,155
Repeat density (% genome)	0.927
BUSCO completeness (%)	99.2 (S: 99.2; D: 0.0; F: 0.8; M: 0.0)

### Reference genome panel selection and genome-based taxonomic placement of NG-CE01

**Fig. 1:**
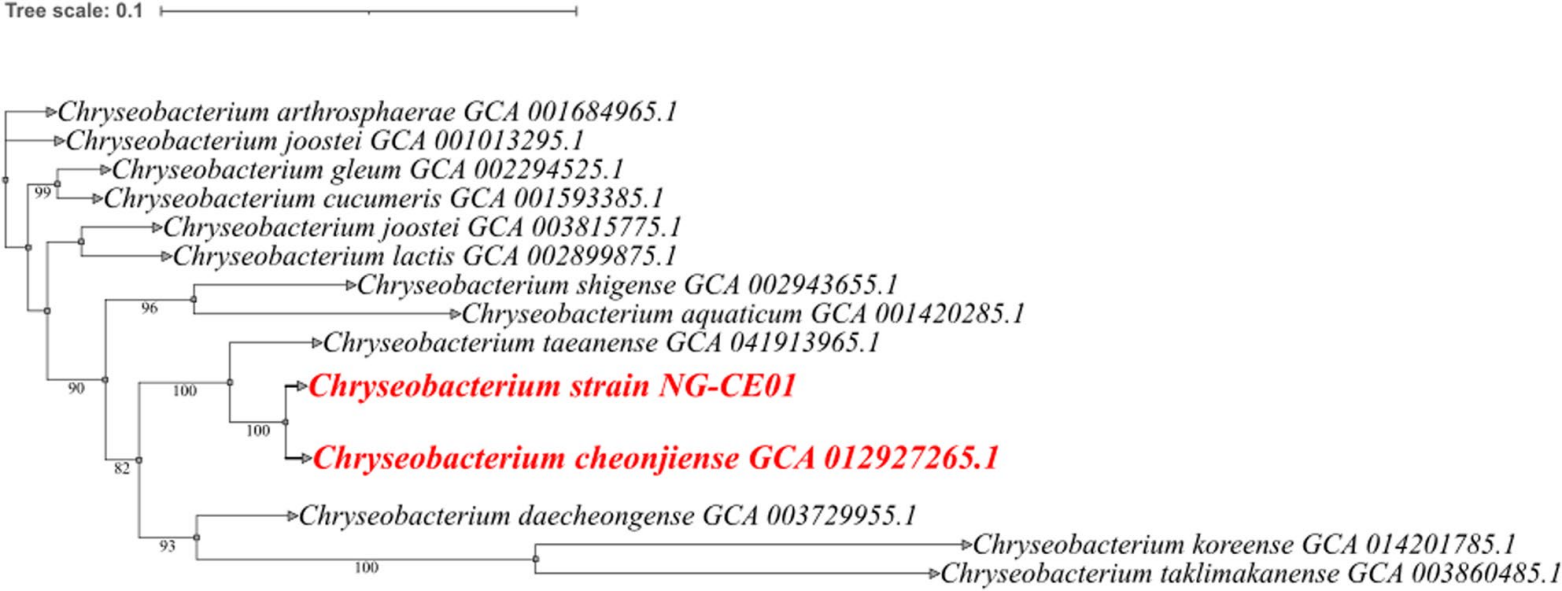
Genome-based phylogenetic placement of *Chryseobacterium* sp. NG-CE01 within the genus *Chryseobacterium*. Maximum-likelihood phylogeny of NG-CE01 and 13 reference genomes inferred with IQ-TREE from conserved marker alignments. NG-CE01 and its closest reference (*C. cheonjiense* GCA_012927265.1) are highlighted in red. Bootstrap support values ≥ 70% (1,000 replicates) are shown. The tree is midpoint-rooted, and the scale bar represents 0.1 substitutions per site

A reference panel of *Chryseobacterium* genomes with comparable genome sizes and GC contents was assembled and evaluated using BUSCO, showing high completeness and minimal fragmentation suitable for downstream comparative analyses (Supplementary Tables S5A–S5B). Phylogenomics based on concatenated conserved markers placed NG-CE01 within *Chryseobacterium* (Fig. 1) and resolved a strongly supported sister relationship with *C. cheonjiense* (100% bootstrap; branch length 0.0134 substitutions/site), with this pair positioned relative to *C. taeanense* (100% bootstrap; branch length 0.02188). Additional internal nodes supporting the NG-CE01 subclade also showed high support (82–93%) (Supplementary Table S6). Consistent with the phylogenomic topology, FastANI identified *C. cheonjiense* (GCA_012927265.1) as the closest available reference (ANI = 93.1206%; aligned fragments 1213/1443; AF = 84.06%), whereas all other pairwise comparisons were markedly lower (79.1442–82.5490%) (Supplementary Table S7). TYGS similarly supported interpretation of NG-CE01 as a putative novel genomospecies (candidate taxon) within the genus: dDDH against *C. cheonjiense* RJ-7-14 yielded d4 = 49.5% (CI 46.8–52.1), below the 70% boundary, despite higher gene-content estimators (d0 = 71.4; d6 = 68.4). The extracted 16 S rRNA gene showed 99.04–99.10% identity to *C. cheonjiense* but only 95–96% query coverage, consistent with limited species-level resolution of 16 S within *Chryseobacterium* (Supplementary Table S8). Together, phylogenomics, ANI, and dDDH support NG-CE01 as *Chryseobacterium* sp., representing a genomically distinct lineage most closely related to *C. cheonjiense*. We therefore report NG-CE01 as a putative novel genomospecies and reserve formal species description for future dedicated taxonomic work.

### Genome architecture and functional composition

**Fig. 2:**
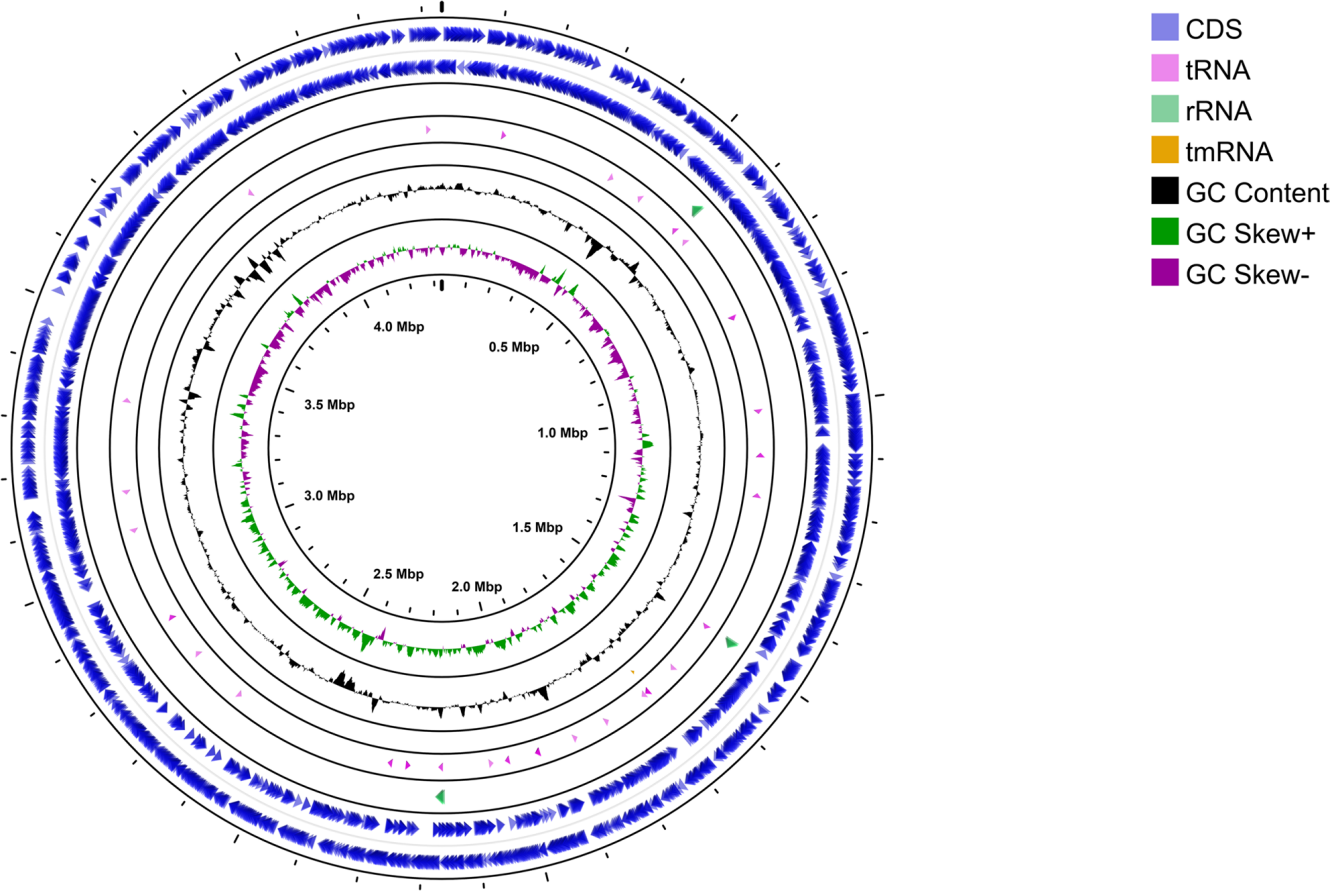
Circular genome map of *Chryseobacterium* sp. NG-CE01. Tracks (outer to inner) show coding sequences (CDS) on forward and reverse strands, tRNA, rRNA, and tmRNA features, followed by GC content and GC skew across the 4.33 Mb single-contig assembly. Positive and negative GC skews are shown as separate tracks

**Fig. 3:**
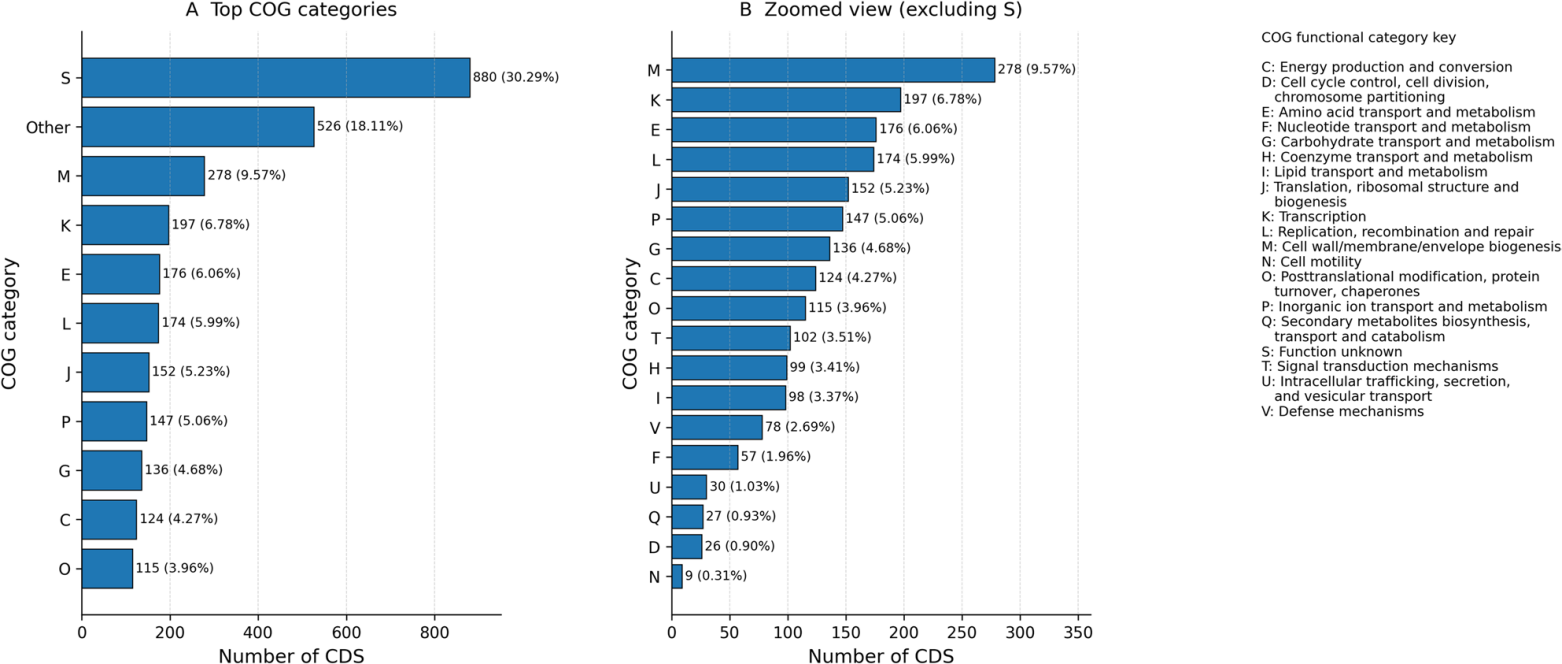
COG functional category distribution in*Chryseobacterium*sp. NG-CE01. Bar plots summarize counts of CDS assigned to COG categories. **A** Top categories including COG S (function unknown) and a pooled “Other” group. **B** Zoomed view excluding COG S to resolve relative contributions among assigned categories

Genome organization and functional capacity of *Chryseobacterium* sp. NG-CE01 were assessed using Prokka annotation with eggNOG-based COG assignment. The genome comprises a single 4,331,766 bp contig (GC 37.43%) encoding 3,923 CDS, 9 rRNA genes, 63 tRNA genes, and 1 tmRNA gene (Table 1). A circular genome map summarizes CDS/RNA distribution together with genome-wide GC content and GC skew (Fig. 2). GC and GC-skew profiles were broadly uniform, with no abrupt compositional shifts (Fig. 2). COG profiling showed that category S (function unknown) represented the largest fraction of annotated CDS (880; 30.29%), followed by a pooled “Other” group aggregating lower-abundance categories (Fig. 3A). When focusing on assigned functions (Fig. 3B), the most abundant categories were cell wall/membrane/envelope biogenesis (COG M; 278, 9.57%), transcription (COG K; 197, 6.78%), amino acid transport/metabolism (COG E; 176, 6.06%), replication/recombination/repair (COG L; 174, 5.99%), and translation/ribosomal biogenesis (COG J; 152, 5.23%). Additional major contributions included inorganic ion transport (COG P; 147, 5.06%), carbohydrate transport/metabolism (COG G; 136, 4.68%), and energy production/conversion (COG C; 124, 4.27%). Moderately represented categories comprised COG O (115, 3.96%), COG T (102, 3.51%), COG H (99, 3.41%), and COG I (98, 3.37%), whereas lower-frequency classes included COG V (78, 2.69%), COG F (57, 1.96%), COG U (30, 1.03%), COG Q (27, 0.93%), COG D (26, 0.90%), and COG N (9, 0.31%) (Fig. 3). Overall, the prominence of envelope biogenesis, genome maintenance, and broad transport/metabolic functions is consistent with a metabolically versatile genome rather than streamlining toward reduction.

### Comparative gene-content diversity across the curated comparator panel and NG-CE01-specific functional hotspots

**Fig. 4:**
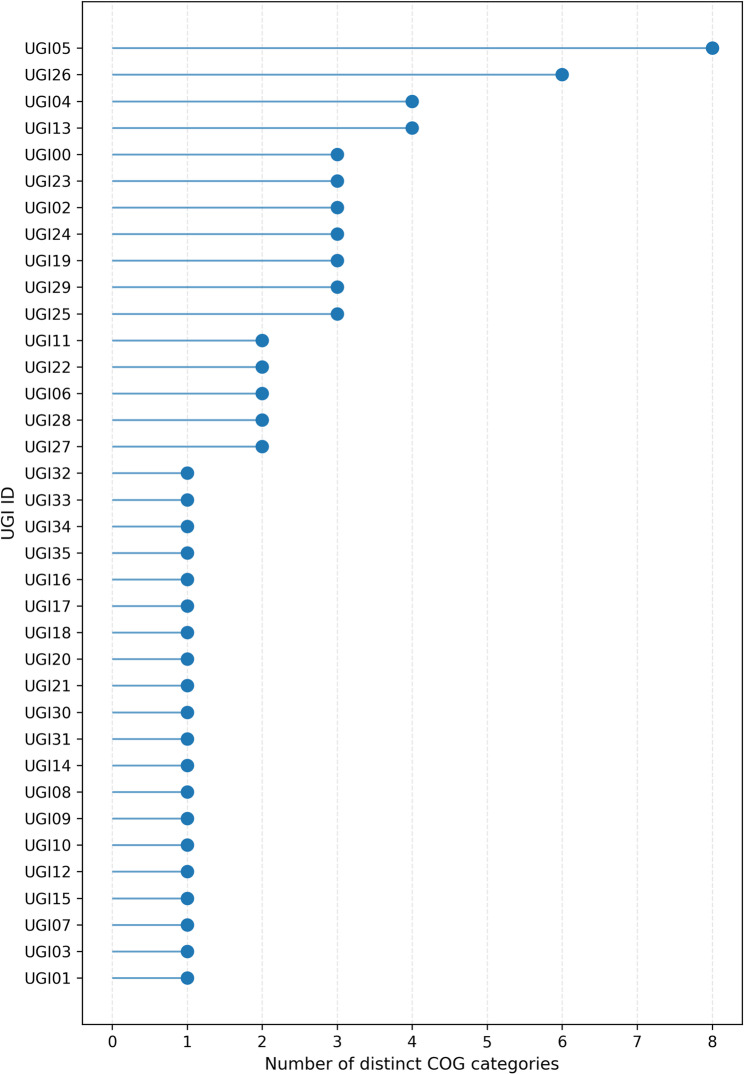
Functional breadth of predicted unique-gene islands (UGIs) in NG-CE01. Lollipop plot showing the number of distinct COG categories represented within each UGI, calculated from COG-assigned genes among the NG-CE01 Unique90 set (Unique90 = gene clusters present in NG-CE01 and absent from all 13 comparator genomes in the curated panel)

**Fig. 5:**
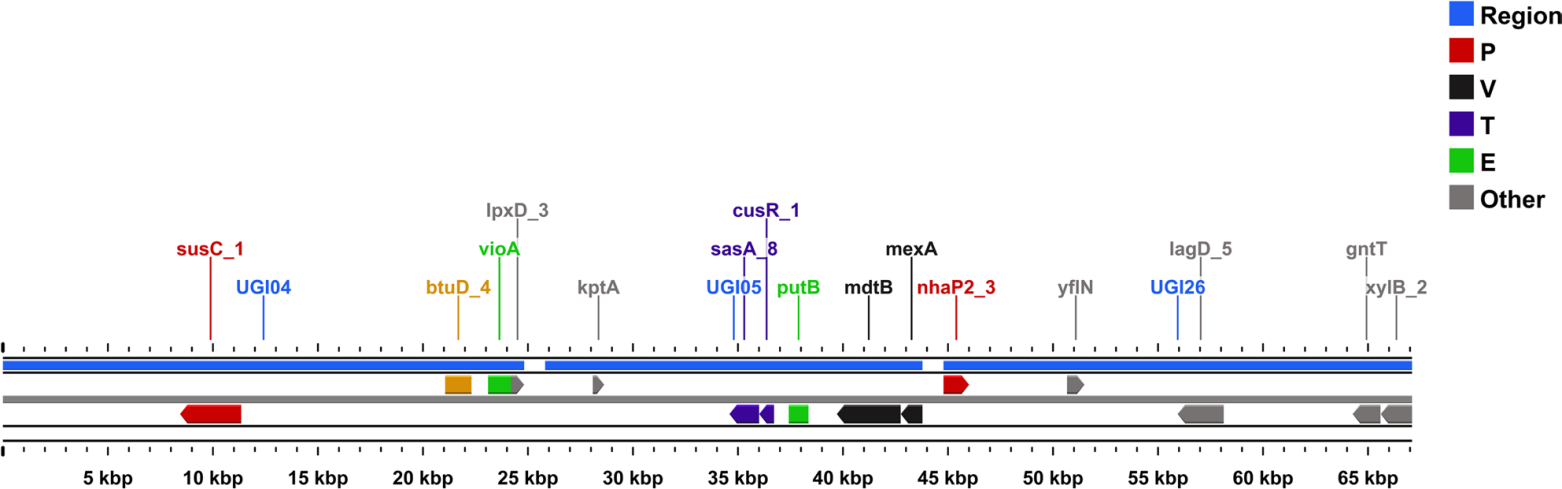
Gene-level context of the highest-scoring Unique90 UGIs in NG-CE01. Linear map showing the genomic locations of UGI04, UGI05, and UGI26 along the NG-CE01 chromosome. Colored blocks denote COG functional categories for annotated Unique90 genes (grey = other categories); the blue track indicates the genomic region, and the scale is shown in kb

Comparative gene-content analysis across NG-CE01 and 13 reference *Chryseobacterium* genomes (*N* = 14) indicated high accessory diversity across this multi-species comparator panel, as expected at interspecies scale (Supplementary Figs. S6–S8; Supplementary Tables S9–S10A). Across 500 genome-order permutations, the mean gene-cluster total increased to 22,436 at 14 genomes (Supplementary Table S9; Supplementary Fig. S6), and the final genome addition contributed 1,126.3 ± 503.2 new clusters (mean ± SD) (Supplementary Table S9; Supplementary Fig. S7). Heaps’ law modeling yielded α = 0.6897 (95% CI 0.5824–0.8473) for the full dataset, with α < 1 across nested panels (*n* = 8, 10, 12, 14) (Supplementary Table S10A; Supplementary Fig. S8). These accumulation statistics are reported descriptively to summarize gene-content diversity across the comparator panel and are not interpreted as a species-level pangenome property of NG-CE01. Under the applied thresholds, gene-cluster frequencies were dominated by accessory content: 39 core clusters (99–100% genomes; 0.17%) versus 22,397 accessory clusters (99.83%), driven primarily by cloud genes (18,152; 80.9%) with a smaller shell fraction (4,245; 18.92%) and no soft-core component (Table 2). As a panel-composition robustness check, repeating Panaroo on an ANI-selected closest-only subset (NG-CE01 plus the seven highest-ANI references; *N* = 8) increased the inferred core to 523 clusters, as expected for a taxonomically narrower panel (Supplementary Table S10B). NG-CE01 contributed 738 isolate-specific clusters (3.29%); most were hypothetical (648; 87.8%), leaving 90 annotated unique genes (12.2%) for downstream functional interpretation (Table 2). Unique90 denotes these 90 annotated, panel-defined isolate-specific genes (present in NG-CE01 and absent from all 13 comparator genomes), retained to focus downstream hotspot and functional analyses on interpretable candidates. These 90 genes mapped to unique-gene islands (UGIs) with uneven functional breadth, with most UGIs carrying few COG categories and a small subset concentrating multi-category diversity (Fig. 4; Supplementary Table S11). Using score = (unique genes) + (COG-assigned genes) + (distinct COG categories), three hotspots were prioritized: UGI05 (9 unique genes; 9 COG-assigned; 8 distinct COG categories; score 27), UGI26 (6; 6; 6; score 18), and UGI04 (6; 6; 4; score 18). UGI04 was enriched in transport-associated functions (COG P), UGI05 in regulatory (COG T) and defense-associated functions (COG V), and UGI26 combined ion transport and carbohydrate-related functions with additional defense-associated genes (Fig. 5); the heatmap confirmed that multi-category signals were concentrated in few UGIs (Fig. 5). Enrichment testing versus the whole-genome background identified a focused signature (Fig. 7; Supplementary Table S12): COG V enriched (12 observed vs. 2.42 expected; 4.97-fold; FDR = 5.8 × 10⁻⁵) and COG L enriched (14 vs. 5.39; 2.60-fold; FDR = 0.007), with COG S depleted (16 vs. 27.26; 0.59-fold; FDR = 0.0463); no other categories remained significant after correction. Collectively, these results identify an NG-CE01-specific concentration of defense (COG V) and replication/recombination-associated functions (COG L), providing context for subsequent mobilome and genome-defense analyses.

**Table 2:** Gene-cluster frequency composition across the curated 14-genome comparator panel and NG-CE01-specific clusters (Panaroo; *N* = 14)

Category	Presence frequency across genomes	Gene clusters (*n*)	% of total pangenome
Core	99–100%	39	0.17
Soft-core	95–<99%	0	0
Shell	15–<95%	4,245	18.92
Cloud	0–<15%	18,152	80.9
Accessory genome (Soft-core + Shell + Cloud)	< 99%	22,397	99.83
Total pangenome	0–100%	22,436	100
Unique to NG-CE01 (subset of Cloud)	Present in 1/14 genomes (7.14%)	738	3.29
└─ Hypothetical proteins (unique)	Present in 1/14 genomes (7.14%)	648	2.89
└─ Non-hypothetical/annotated (unique)	Present in 1/14 genomes (7.14%)	90	0.4

**Fig. 6:**
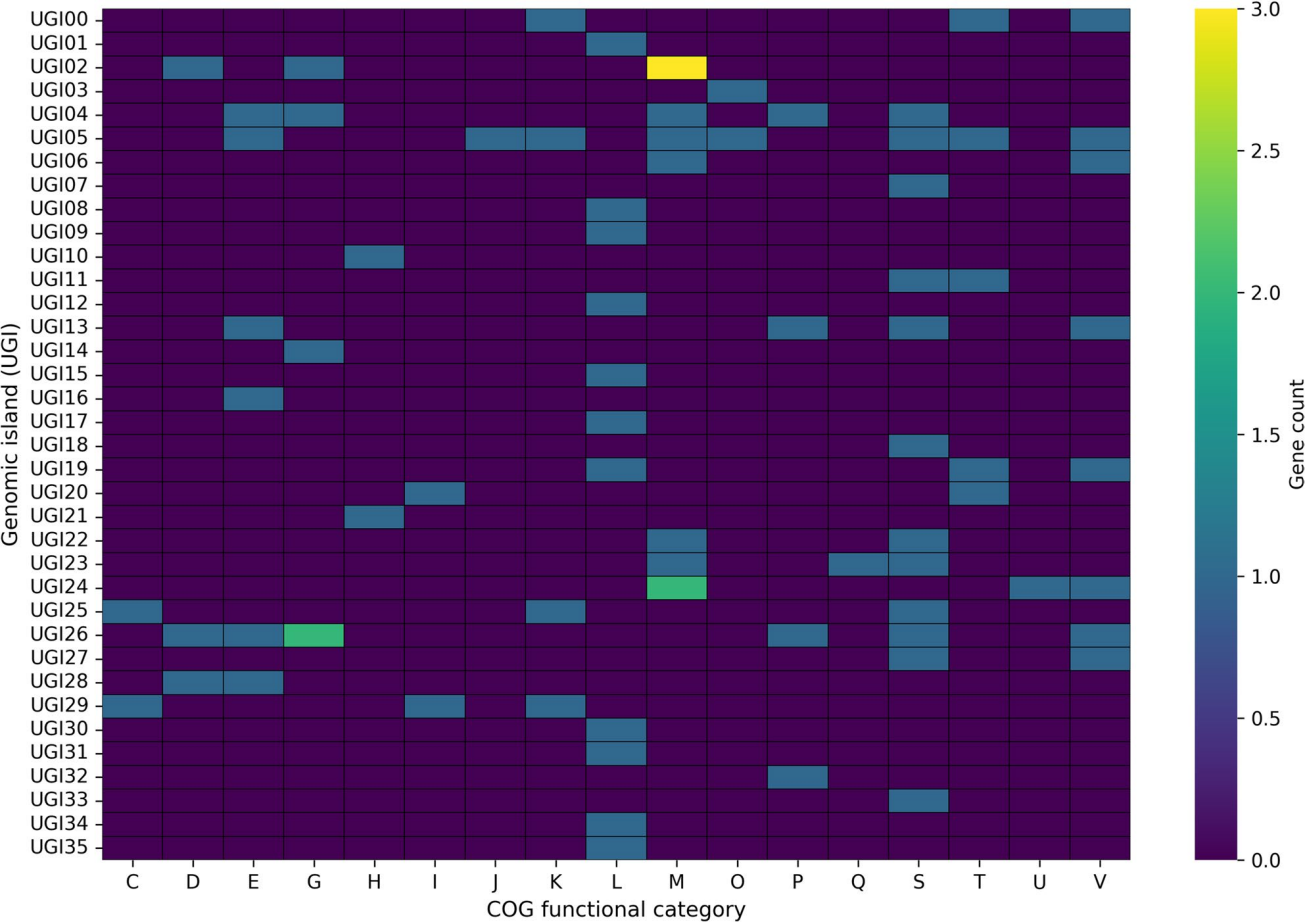
UGI-resolved distribution of COG functional categories among annotated Unique90 genes. Heatmap showing counts of COG-assigned Unique90 genes per UGI. Rows correspond to UGIs and columns to COG categories; color intensity indicates gene count per UGI–COG cell

**Fig. 7:**
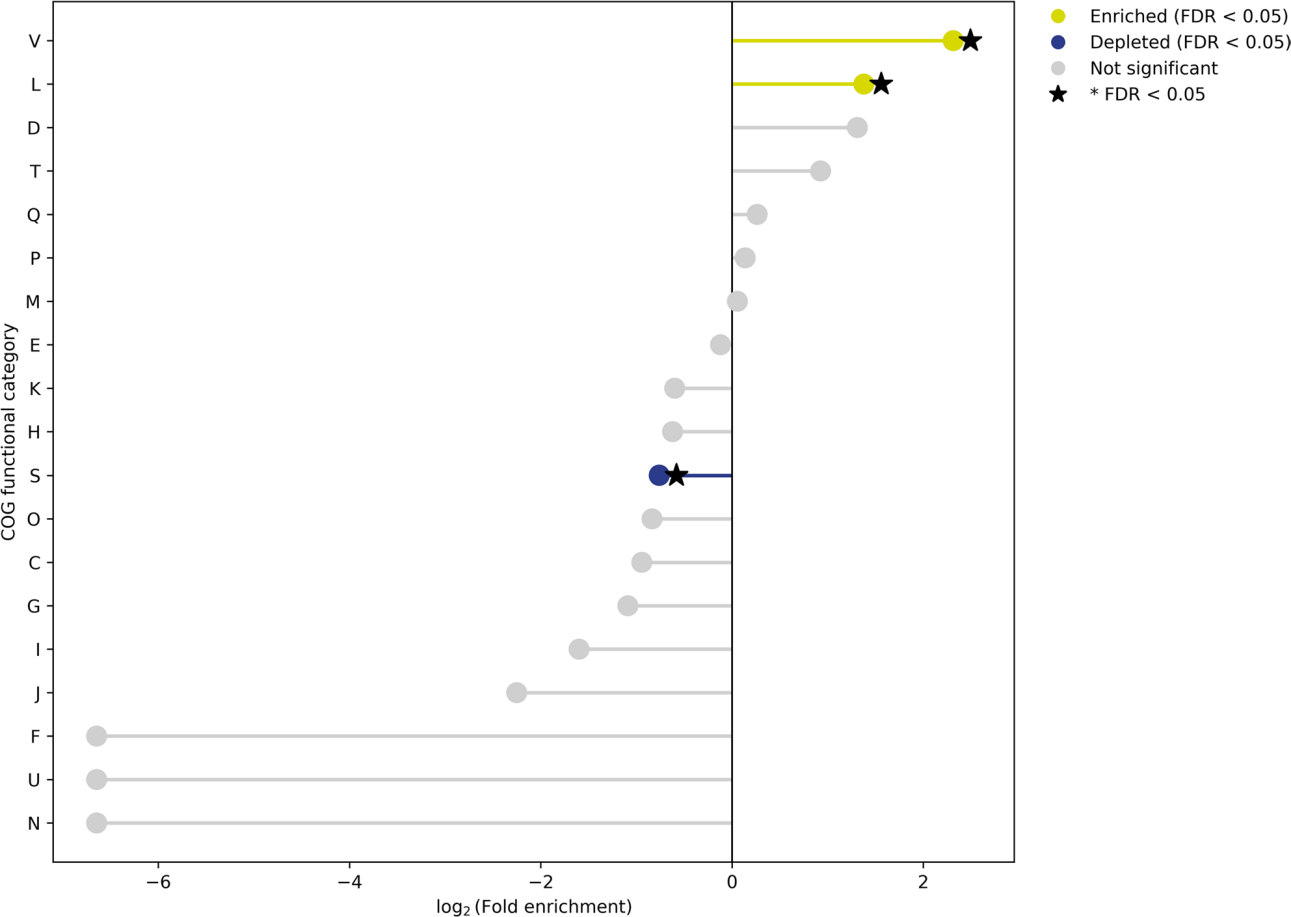
COG functional enrichment among UGI-associated annotated Unique90 genes. Log2 fold-enrichment of COG categories among COG-assigned Unique90 genes relative to whole-genome background. Points indicate enrichment (positive) or depletion (negative); categories significant after BH-FDR correction are marked with an asterisk (FDR < 0.05)

### CAZyme repertoire, class-level shifts, and NG-CE01–specific domain architectures

**Fig. 8:**
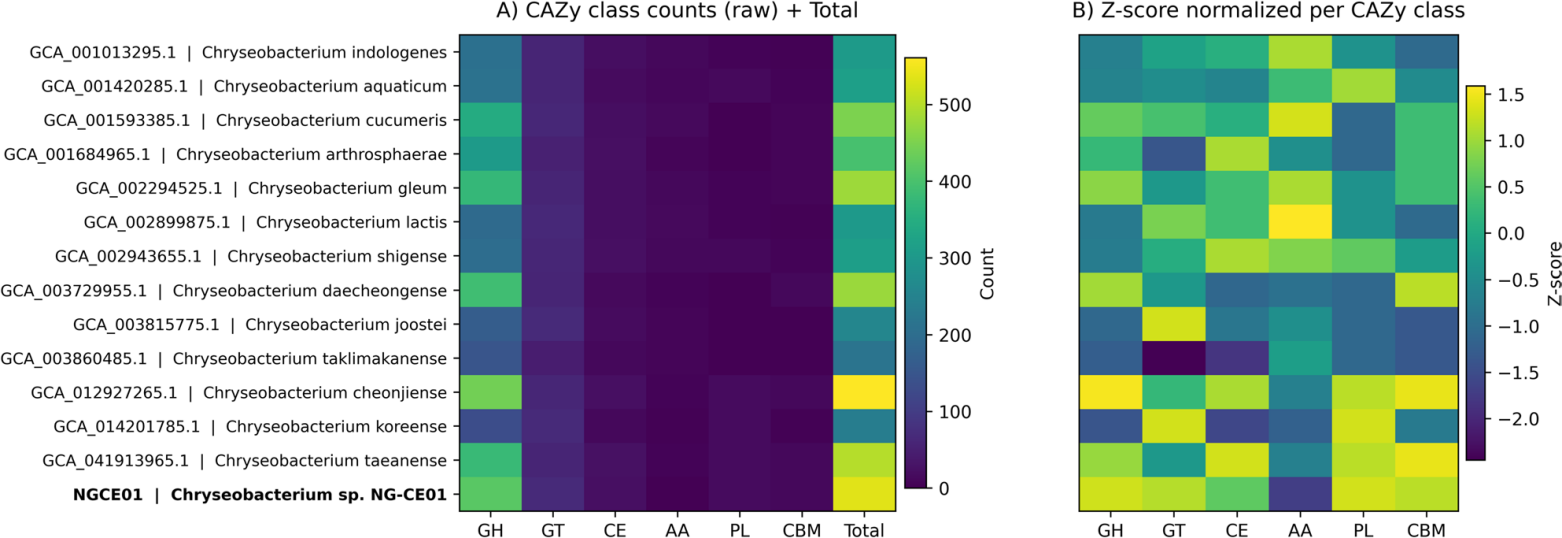
CAZyme class composition across NG-CE01 and reference*Chryseobacterium*genomes. **A** Heatmap of raw CAZyme class counts per genome with total CAZyme burden shown as an additional column. **B** Z-score–normalized CAZyme class composition across genomes. CAZyme classes include GH, GT, CE, AA, PL, and CBM

NG-CE01 encodes a CAZyme repertoire dominated by glycoside hydrolases (GH), with secondary glycosyltransferases (GT) and smaller contributions from CE, PL, CBM, and AA. GH comprised 417/535 CAZymes (77.9%), followed by GT 66/535 (12.3%), CE 22/535 (4.1%), PL 17/535 (3.2%), CBM 11/535 (2.1%), and AA 2/535 (0.4%) (Supplementary Table S13). This GH-first hierarchy was conserved across the 13 reference genomes; however, NG-CE01 combined a high total CAZyme load with a particularly GH-heavy profile (Supplementary Table S13). Raw class counts and per-class z-scores highlighted deviations from the reference distribution and positioned NG-CE01 among the most class-enriched genomes (Fig. 8). At the family level, NG-CE01 carried rare CAZyme families present in NG-CE01 but detected in ≤ 2/13 references under the applied dbCAN HMMER criteria including AA12 (*n* = 2) and single-copy GH117, GT90, GH1, and GT89 (Supplementary Table S14). At the protein-architecture level, NG-CE01 contained Unique90 multi-domain CAZyme genes, indicating combinatorial novelty beyond class abundance (Supplementary Table S15). These included GT fusions (e.g., GT2 + GT2), mixed GT4 + GT5 architectures, and a prominent GH130 multi-copy, subfamily-rich configuration, each supported by strong domain evidence (Supplementary Table S15). Together, class composition (Supplementary Table S13; Fig. 8), rare families (Supplementary Table S14), and distinctive multi-domain architectures (Supplementary Table S15) define an NG-CE01 CAZyme profile enriched in both abundance and domain-level diversification relative to closely related genomes.

### Locus-scale synteny remodeling defines carbohydrate-foraging plasticity in NG-CE01

**Fig. 9:**
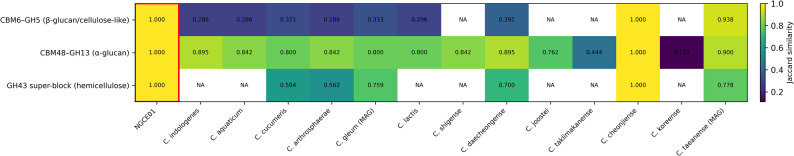
Locus-scale synteny similarity of three carbohydrate-foraging blocks across NG-CE01 and reference *Chryseobacterium* genomes. Jaccard similarity of three blocks (CBM6–GH5, CBM48–GH13, GH43) across NG-CE01 and 13 reference genomes. Homolog-group fingerprints were derived from ± 10 kb windows using clinker; NA indicates block absence

The CAZyme repertoire of NG-CE01 was GH-dominant and included rare families and NG-CE01–specific multi-domain architectures (Supplementary Tables S13–S15; Fig. 8). To distinguish whether NG-CE01’s carbohydrate-foraging diversification is driven primarily by CAZyme burden versus locus-scale architectural remodeling, we performed synteny-guided locus analysis on three anchor-defined carbohydrate-foraging blocks spanning distinct substrate spaces: CBM48–GH13 (α-glucans), CBM6–GH5 (β-glucans/cellulose-like), and a GH43-rich hemicellulose “super-block.” For each block, homologous ± 10 kb windows were extracted across 13 reference genomes to quantify local gain/loss and rearrangement at the neighborhood scale rather than global proteome similarity. Across the three blocks, significant BLASTP hits from NG-CE01 window proteins mapped predominantly within the homologous reference windows (CBM48–GH13: 192/271, 70.8%; GH43: 173/293, 59.0%; CBM6–GH5: 84/178, 47.2%), with the remaining hits outside these windows, indicating overall neighborhood coherence while highlighting broader dispersal of CBM6–GH5–associated components. Locus similarity was then quantified using clinker homolog-group fingerprints and pairwise Jaccard similarity relative to NG-CE01 (Supplementary Table S16; Fig. 9). CBM6–GH5 was detected in 9/13 references; NG-CE01 encoded 16 homolog groups, and most locus-positive references were strongly diverged (J = 0.286–0.391), whereas *C. cheonjiense* matched identically (J = 1.000) and *C. taeanense* was highly similar (J = 0.938) (Supplementary Table S15; Supplementary Table S16; Supplementary Fig. S11; Fig. 9). CBM48–GH13 was conserved in 13/13 references; the NG-CE01 window (18 homolog groups) spanned a wide similarity range (J = 0.111–1.000), with maximal divergence in *C. koreense* (J = 0.111), intermediate similarity in *C. taklimakanense* (J = 0.444), and high similarity to *C. cheonjiense* (J = 1.000) and *C. taeanense* (J = 0.900) (Supplementary Table S16; Supplementary Fig. S10; Fig. 9). The GH43 super-block showed the lowest prevalence (6/13); NG-CE01 encoded 24 homolog groups, and locus-positive genomes ranged from moderate divergence to identity (J = 0.562–1.000; e.g., *C. arthrosphaerae* 0.562; *C. cucumeris* 0.594; *C. daecheongense* 0.700; *C. cheonjiense* 1.000; *C. taeanense* 0.778) (Supplementary Table S16; Supplementary Fig. S12; Fig. 9). Together, these patterns indicate conserved scaffolds where present but substantial local gain/loss and re-organization across genomes, supporting locus-scale remodeling as the dominant signal underlying carbohydrate-foraging diversification rather than CAZyme counts alone (Supplementary Table S16; Fig. 9; Supplementary Figs. S10–S12). To test whether these blocks behave as co-inherited modules, we applied Scoary to the Panaroo gene-presence/absence matrix (22,436 clusters; 14 genomes) using traits for GH43 super-block presence (7/14 positive) and CBM6–GH5 presence (10/14 positive); CBM48–GH13 was conserved (14/14) and not testable. Under BH-FDR correction, Scoary identified 123 clusters associated with GH43 (minimum BH-FDR 0.0469) and 236 associated with CBM6–GH5 (minimum BH-FDR 0.0167; range 0.0167–0.0246). Enriched annotations included TonB/Sus-like uptake components, transporters/symporters, and transcriptional regulators alongside many hypothetical proteins, consistent with coordinated inheritance of uptake, regulation, and accessory functions.

### Genomic Island delineation and compositional support

Genomic island (GI) screening of the NG-CE01 main contig (edge_1) identified three discrete regions totaling 108.4 kb and 96 CDS (Supplementary Table S17): GI_1 (edge_1: 437,180–461,873; 24,694 bp; 26 genes), GI_2 (edge_1: 1,693,707–1,709,594; 15,888 bp; 13 genes), and GI_3 (edge_1: 3,811,259–3,879,037; 67,779 bp; 57 genes). All islands were enriched in hypothetical proteins, most prominently in GI_1 (23/26; 88.5%) and GI_3 (46/57; 80.7%), with a lower fraction in GI_2 (5/13; 38.5%) (Supplementary Table S17). Compositional metrics supported distinct signatures across islands. Relative to the chromosomal background (GC 37.43%), GI_1 was strongly AT-shifted (GC 30.09%; ΔGC − 7.34%), whereas GI_2 closely matched background (GC 37.97%; ΔGC + 0.54%) and GI_3 remained near-background (GC 36.69%; ΔGC − 0.74%). Mean GC-skew values were modest but distinguishable (GI_1 − 0.039; GI_2 + 0.104; GI_3 − 0.047) (Supplementary Table S18). Sliding-window profiles across each island and ± 10 kb flanks showed a pronounced GC depression for GI_1 but subtler shifts for GI_2 and GI_3 (Supplementary Fig. 13).

### Gene-content anchors and Unique90 overlap

Despite high hypothetical content, each island contained interpretable gene-content anchors (Supplementary Table S19). GI_1 encoded xerC (HEDAFHDN_00447) together with two additional annotated genes (HEDAFHDN_00452, ethylmalonyl-CoA/methylmalonyl-CoA epimerase; HEDAFHDN_00453, 30 S ribosome-binding factor) but showed no overlap with the Unique90 set (0/90) (Supplementary Table S20). GI_2 comprised a large housekeeping cassette (nusG; rplK/rplA/rplJ/rplL; rpoB/rpoC) and a single mobility-associated gene, an IS110-family transposase (HEDAFHDN_01658) (Supplementary Table S19); it overlapped with 1/90 Unique90 genes (the IS110 transposase), assigned to a defined UGI (Supplementary Tables S16, S18). In contrast, GI_3 concentrated the isolate-linked signal, containing 4/90 Unique90 genes (HEDAFHDN_03549, HEDAFHDN_03554, HEDAFHDN_03555, HEDAFHDN_03576) mapped to UGI29–UGI30 (Supplementary Table S20), including two reductases (2-hydroxy-3-oxopropionate reductase; 2-haloacrylate reductase), a transcriptional regulator (hxlR), and an IS3-family transposase.

### Functional partitioning by COG composition and enrichment

COG aggregation revealed distinct functional signatures across islands (Supplementary Tables S21–S22; Supplementary Fig. 14). GI_1 contained 10 COG-assigned CDS, dominated by COG L (4/10, 40.0%) and COG K (2/10, 20.0%). GI_2 contained 13 COG-assigned CDS, with the largest contributions from COG J and COG K (each 4/13, 30.8%). GI_3 contained 44 COG-assigned CDS and was dominated by COG M (11/44, 25.0%) and COG K (9/44, 20.5%) (Supplementary Tables S21–S22). Enrichment testing against the whole-genome background with BH-FDR correction supported GI_1 enrichment for COG L (fold-change 6.81; Fisher *p* = 0.00196; BH-FDR = 0.0373) and GI_3 enrichment for COG M (fold-change 2.68; Fisher *p* = 0.00218; BH-FDR = 0.0212) (Supplementary Table S23; Supplementary Fig. 14). For GI_2, increases in COG J (fold-change 6.01; *p* = 0.00356; BH-FDR = 0.0675) and COG K (fold-change 4.61; *p* = 0.00902; BH-FDR = 0.0857) did not remain significant after multiple-testing correction (Supplementary Table S23).

### Cross-genome conservation and synteny-supported interpretation

Comparative conservation across 13 reference *Chryseobacterium* genomes placed the three islands along a clear spectrum (Supplementary Tables S24–S25). GI_2 was broadly conserved, with merged coverage reaching 92.239% (14,655 bp) in the best match, high mean identity (96.332%), and few high-scoring segment pair (HSP) blocks (Supplementary Table S24). In contrast, GI_3 showed weak and mosaic conservation: the best reference covered 43.373% (29,398 bp) distributed across 25 HSPs, whereas most other genomes exhibited ≤ 12.734% coverage (Supplementary Table S25). Jaccard overlap analyses supported the same split: GI_2 reached J = 0.923 (12/13 shared) in the best match, whereas GI_3 overlapped detectably in only one reference (J = 0.211; 12/57 shared), with 12 genomes showing 0/57 shared content (Supplementary Table S26). These conservation profiles guided the synteny visualization strategy. GI_2 was displayed as a multi-genome clinker panel using the top five overlap references, whereas GI_3 was visualized only against the single best reference to avoid empty tracks in low-overlap comparisons (Supplementary Tables S26–S27; Supplementary Figs. 15–16). GI_1 showed minimal overlap (top 6/26; J = 0.231; with many references ≤ 2/26 or 0/26), rendering multi-genome synteny uninformative beyond quantitative conservation summaries (Supplementary Tables S26–S27).

### A consistent novelty gradient across GI_1–GI_3

Across compositional metrics, annotation density, COG structure, Unique90 overlap, and cross-genome conservation, the three islands formed a consistent novelty gradient (Supplementary Tables S18–S26; Supplementary Figs. 13–16). GI_2 showed near-background composition (ΔGC + 0.54%), the lowest hypothetical burden (38.5%), and high conservation (coverage up to 92.239%; J = 0.923), consistent with a widely distributed locus (Supplementary Tables S16, S19, S24, S25). GI_1 exhibited the strongest compositional shift (ΔGC − 7.34%), extreme hypothetical dominance (88.5%), no Unique90 overlap, and low/inconsistent conservation (Supplementary Tables S16, S24, S25; Supplementary Fig. 13). GI_3 combined large size (67.8 kb), enrichment for envelope functions (COG M; BH-FDR 0.0212), four Unique90 genes mapped to UGI29–UGI30, and near-zero conservation except for a single partial overlap (J = 0.211) (Supplementary Tables S18–S26; Supplementary Figs. 14–16). Collectively, these features identify GI_3 as the principal island-level reservoir of NG-CE01 isolate-specific functional novelty, contrasted with a conserved GI_2 and a compositionally divergent but sparsely annotated GI_1.

### Mobilome landscape of *Chryseobacterium* sp. NG-CE01

#### Insertion sequences and transposase repertoire

Insertion sequence (IS) content was screened using ISfinder (E-value ≤ 1 × 10⁻³) and locally validated by read remapping, locus-context inspection, and Pfam domain confirmation to distinguish intact transposases from motif-level similarities (Supplementary Tables S28–S29). ISfinder returned three high-confidence queries on edge_1 ISSau3 and ISSmi2 (IS1182 family) and ISLpn9 (IS4/IS10 group) (Supplementary Table S28) but local evidence supported only limited, erosion-consistent signatures rather than confidently bounded IS elements. The two IS1182 hits repeatedly mapped to the same ~ 1.14 Mb neighborhood and overlapped a single CDS (ORF 1_4) with Pfam support for a DDE transposase (DUF772/PF05598; DDE_Tnp_1/PF01609), consistent with a single transposase-associated locus or degraded IS-like remnant rather than two independent elements (Supplementary Table S28). In contrast, the IS4/IS10-like query (ISLpn9) produced only 28–32 bp motif-sized similarities near ~ 3.62 Mb in an intergenic interval without a co-localized transposase CDS, arguing against an intact IS4/IS10 element (Supplementary Table S28). To cross-validate independently of ISfinder similarity, Panaroo Unique90 candidates annotated as IS/transposase-like (COG L) were screened and Pfam-confirmed (Supplementary Tables S30–S31). This yielded a compact set dominated by IS110-family enzymes (Transposase_20/PF02371 and DEDD_Tnp_IS110/PF01548), whereas other candidates showed truncated profiles (Supplementary Table S30). Inspection of representative IS110 loci (HEDAFHDN_01658; HEDAFHDN_02294) recovered intact central transposase ORFs with strong Pfam support but weak terminal signatures and no detectable TSDs, consistent with eroded ends (Supplementary Table S32). Validated IS/transposase loci were not enriched within the three curated genomic islands: only HEDAFHDN_01658 localized to GI_2, while other candidates occurred outside GI_1–GI_3. Collectively, these results indicate a restrained mobilome dominated by few, erosion-consistent IS-like loci, and support interpretation of island-level functional novelty particularly GI_3 as not being driven by transposase clustering.

#### Prophage remnants

Prophage screening detected no intact prophages. PHASTER identified one incomplete prophage-like region on edge_1 (2,406,137–2,428,202; 22,066 bp; score 20; 14 CDS) (Supplementary Table S33). Gene-level inspection revealed only sparse matches to phage-associated functions (e.g., methyltransferase, tail assembly, HTH-like regulator) within a background of predominantly hypothetical CDS, consistent with a degraded prophage remnant rather than an inducible prophage (Supplementary Table S34). Cross-tool comparison supported this conservative interpretation, as PHASTEST reported no prophage for the same interval. This remnant is separate from GI_1–GI_3 and from the ICE-like locus described below, indicating that mobilome signals are dispersed rather than co-localized.

#### Plasmids and integron-associated elements

PlasmidFinder screening returned no plasmid replicons and no plasmid sequence evidence, consistent with a mobilome profile dominated by chromosomal features rather than an extrachromosomal replicon. IntegronFinder similarly detected no evidence of integrons, supporting the absence of integron-mediated gene capture in NG-CE01.

#### T4SS-associated mobilome

ICEfinder (ICEberg 3.0) identified a single mobility-associated locus with type IV secretion system (T4SS) features on edge_1 (3,720,179–3,736,023; 15,845 bp; GC 42.16%) (Supplementary Tables S35–S37). The region encodes a compact conjugation-associated module, including a type IV coupling protein (T4CP; VirD4-like), multiple Tra/VirB components (e.g., TraN/TraM/TraK/TraJ; VirB4-like ATPase), and a MOBP-family relaxase (Supplementary Tables S35–S37). However, classical ICE integration hallmarks were not definitive within the called coordinates (no clear tRNA association and no canonical short, near-perfect attachment (att)-like boundary repeat in the immediate flanks) (Supplementary Tables S36–S37). Accordingly, we interpret this locus conservatively as a putative conjugation-/mobility-associated region rather than a fully resolved integrative conjugative element. Overall, NG-CE01 shows a low-to-moderate mobilome burden, comprising no plasmid replicons, one incomplete prophage remnant (PHASTER), a compact transposase repertoire, and this single cargo-poor mobility-associated region. The prophage remnant, this mobility-associated region, and curated genomic islands are geographically separated, supporting analysis of each mobilome layer as distinct and indicating limited evidence for extensive recent mobile-element–driven remodeling. 

#### Resistance, fitness-associated loci, and genome-defense repertoire of *Chryseobacterium* sp. NG-CE01

**Fig. 10:**
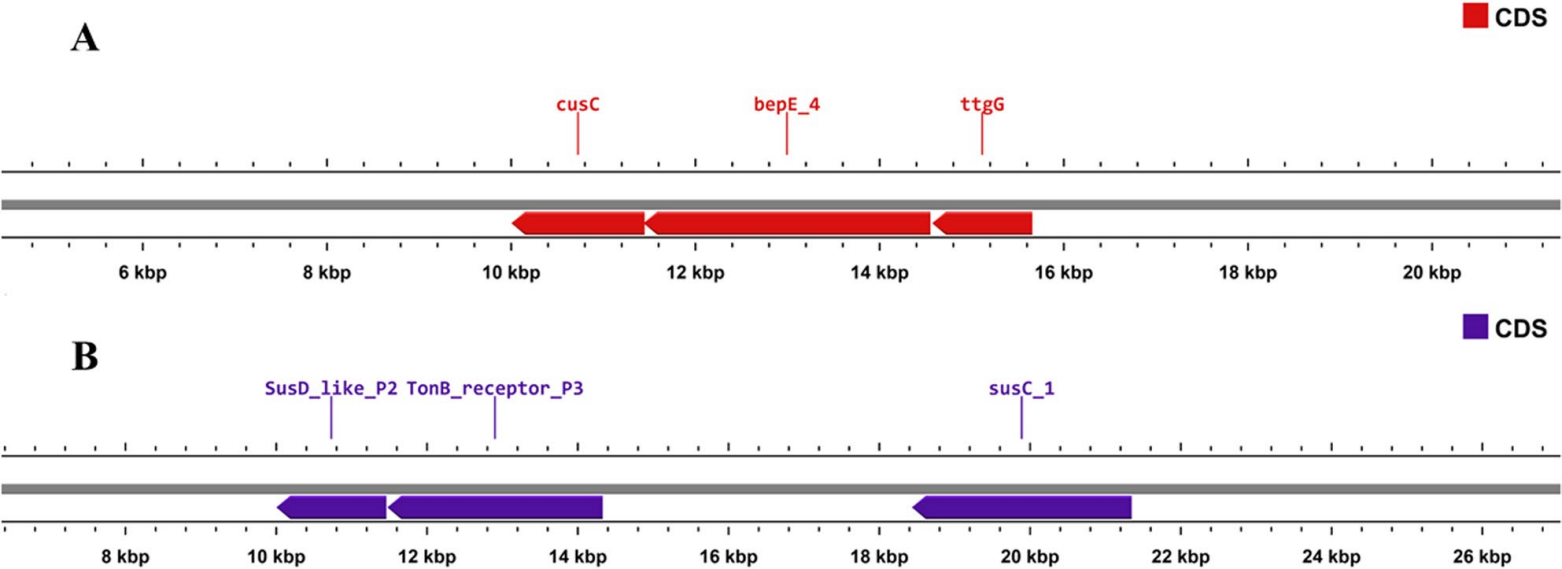
Genomic organization of curated chromosomal loci in *Chryseobacterium* sp. NG-CE01. **A** R2 efflux/permeability module (cusC–bepE_4–ttgG). **B** V1 Sus-like uptake module (SusD-like_P2–TonB_receptor_P3–susC_1). CDS are shown as directional blocks with coordinates in kb

Standard database screening detected no acquired AMR determinants (AMRFinderPlus, ResFinder, CARD) and no VFDB virulence factors; genome-defense features were limited to toxin–antitoxin (parE/parD) and Type I restriction subunits (hsdR), with no CRISPR arrays (Table 3). To link these negative acquired AMR/virulence results to the isolate-specific pangenome signal, we interrogated the Unique90 set (*n* = 90; Panaroo presence/absence matrix; Supplementary Table S11) and curated 24/90 genes (26.67%) into chromosomal loci (Supplementary Table S38): intrinsic resistance/AMR-like loci R1–R5 (11 genes; 12.22%), fitness/virulence-like loci V1–V5 (8 genes; 8.89%), and genome-defense loci D1–D2 (5 genes; 5.56%). The remaining 66/90 genes (73.33%) could not be assigned to curated locus categories (Supplementary Table S38). These curated modules were chromosomal and supported by neighborhood coherence rather than acquired-resistome signatures (Supplementary Table S38). As examples, we mapped R2 (Fig. 10A), a metal/solvent efflux neighborhood (cusC–bepE_4–ttgG; edge_1: 3,632,842–3,658,499), and V1 (Fig. 10B), a SusC/SusD/TonB uptake module (SusD-like_P2–TonB_receptor_P3–susC_1; edge_1: 813,263–824,609) (Fig. 10A–B; Supplementary Table S39). Cross-genome mapping to the nearest sister (*C. cheonjiense* RJ-7-14; GCA_012927265.1) identified corresponding regions with high nucleotide similarity (R2: 92.215% identity across 9,839 bp; V1: 94.581% across 10,039 bp; both E = 0.0), and synteny plots confirmed neighborhood conservation (Supplementary Fig. S17A–B). Reciprocal Best Hit (RBH) overlaps within the extracted windows indicated partial conservation with accessory turnover (R2: shared_RBH = 5; J = 0.294; V1: shared_RBH = 4; J = 0.200) (Supplementary Fig. S17; Supplementary Table S38). These intrinsic AMR-like loci also align with the disk-diffusion profile (Supplementary Table S4): despite the absence of acquired AMR genes, NG-CE01 encodes chromosomal permeability/efflux-associated modules (e.g., R1–R2) that can modulate baseline susceptibility. Collectively, these results connect (i) negative acquired-resistome screens (Table 3), (ii) the Unique90 isolate-specific signal (Supplementary Table S11), and (iii) antibiotic testing (Supplementary Table S4), indicating that NG-CE01’s distinctive gene content is concentrated in chromosomal functional neighborhoods rather than recognizable acquired AMR/virulence systems.

**Table 3:** Resistance, virulence, and genome defense systems in *Chryseobacterium* sp. NG-CE01

Functional category	Database / Method	Genes detected	Genomic context	Genomic location / coordinates
Antimicrobial resistance	AMRFinderPlus	None	–	– (no hits)
Antimicrobial resistance	ResFinder	None	–	– (no hits)
Antimicrobial resistance	CARD	None	–	– (no hits)
Virulence factors	VFDB	None	–	– (no hits)
Toxin–antitoxin system	HMM (Pfam)	parE/parD	Chromosomal	parE1_1:1,805,333–1,805,626 (−) parE1_2:3,097,116–3,097,415 (−) parD1:3,097,408–3,097,659 (−)
Restriction–modification	Pfam / Prokka	hsdR (Type I RM)	Chromosomal	hsdR_1834,426–2,837,533 (+) hsdR_23,390,327–3,393,185 (+)
CRISPR–Cas	MinCED/CRT Galaxy	None	Chromosomal	No CRISPR arrays detected

## Discussion

The phenotypic profile of *Chryseobacterium* sp. NG-CE01 (yellow pigmentation, pleomorphic Gram-negative rods, oxidative biochemical behavior) is consistent with established genus descriptions [1–3], supporting its placement within *Chryseobacterium*. Its recovery from bovine keratitis provides genome-resolved context for a host-associated ocular setting that remains underrepresented for this lineage. The ocular surface is a selective environment shaped by tear turnover, antimicrobial peptides, immune surveillance, and a glycan-rich mucosal layer [4–6]; persistence in such settings is plausibly supported by metabolic flexibility, controlled permeability, and stress tolerance rather than classical virulence determinants. Whole-genome comparisons place NG-CE01 below commonly used species delineation thresholds (ANI ~93%, dDDH ~49.5%), with phylogenomics identifying *C. cheonjiense* as the closest available reference. The contrast between this genome-wide divergence and relatively high 16S similarity reinforces the value of genome-scale metrics over single-gene markers for resolving *Chryseobacterium* lineages, where 16S resolution can be limited [7–9]. Accordingly, NG-CE01 is best described as a genomically distinct lineage (putative novel genomospecies) within the genus, while formal species description is reserved for future dedicated taxonomic work. At the gene-content level, we explicitly treat Panaroo outputs as an interspecies comparative gene-cluster survey across a curated comparator panel rather than a species-level pangenome property of NG-CE01. As expected for a multi-species panel, gene-content accumulation reflects high accessory diversity; importantly, panel narrowing by ANI increased the inferred core substantially,consistent with taxonomic breadth as a primary driver of “openness.” Within this framework, NG-CE01’s isolate-linked signal is structured rather than diffuse: the annotated “Unique90” subset concentrates into discrete chromosomal hotspots enriched for defense-associated (COG V) and replication/recombination-associated (COG L) functions, supporting localized innovation and neighborhood remodeling rather than genome-wide dominance of recently acquired cargo. This neighborhood-level interpretation is most evident in the carbohydrate-foraging landscape. While CAZyme-rich repertoires broadly support carbohydrate utilization in *Chryseobacterium*, abundance alone does not explain differentiation among related genomes. Instead, synteny-guided analyses indicate remodeling of discrete neighborhoods (CBM48–GH13, CBM6–GH5, and a GH43-rich super-block) that retain conserved anchor organization in subsets of the comparator panel but show graded divergence across references. Jaccard gradients and Scoary associations support that GH43 and CBM6–GH5 behave as co-inherited multi-gene modules, emphasizing locus-scale organization as a major axis of metabolic diversification [10–12]. Given the glycan-rich ocular surface, these modular neighborhoods are compatible with a hypothesis of flexible glycoprotein utilization and persistence under restrictive conditions, but the present evidence remains inferential rather than causal. The genomic-island landscape reinforces the theme of structured novelty. Mobile genetic elements are major drivers of bacterial genome plasticity, facilitating the redistribution, stabilization, and evolutionary testing of accessory genes across lineages [38, 39]. In this context, GI_2 appears broadly conserved across the comparator panel, whereas GI_1 is compositionally shifted and dominated by hypothetical CDS, leaving its contribution unresolved. In contrast, GI_3 represents a major reservoir of isolate-linked innovation, enriched for envelope-related functions, transcriptional regulation, and redox-associated processes with limited conservation beyond the nearest relative. Compositional features and remnant mobility signatures are compatible with historical acquisition, whereas the lack of intact mobility machinery supports stabilization and chromosomal integration rather than ongoing transfer. This interpretation is consistent with the broader view that mobile genetic elements often act as evolutionary delivery systems whose cargo may subsequently become fixed and remodeled within the chromosome even after transfer functions decay [38, 39]. Consistent with this, NG-CE01 exhibits a restrained mobilome overall: a degraded prophage remnant, a limited set of insertion sequence/transposase signatures dominated by IS110-like elements, and a single cargo-poor T4SS-associated mobility region (ICEfinder Region1) best interpreted as a putative conjugation-/mobility-associated locus unless hallmark ICE boundaries and complete core transfer architecture are demonstrated. Notably, although such elements are frequently implicated in the dissemination of antimicrobial resistance and adaptive traits [39], the NG-CE01 assembly did not support a mobilome dominated by recently imported resistance cargo. Finally, Unique90 curation helps contextualize inhibition-zone patterns in the absence of acquired AMR determinants. Standard resistome and VFDB screening did not identify canonical acquired AMR genes or virulence factors, yet isolate-linked genes cluster within chromosomal neighborhoods consistent with chromosome-encoded permeability/efflux and stress-response circuitry and niche fitness rather than imported cassettes. Here, “intrinsic” refers to genome-predicted, chromosome-encoded features and should not be interpreted as clinically validated resistance. Such architecture can plausibly shift inhibition-zone diameters via baseline changes in permeability, efflux, and envelope stress response even when acquired AMR genes are not detected; genome-resolved analysis of a multidrug-resistant C. indologenes isolate similarly reported multiple efflux- and envelope-associated features alongside broad phenotypic non-susceptibility, supporting this concept [40]. Given limited validated CLSI/EUCAST breakpoints for *Chryseobacterium* spp., reporting results primarily as zone diameters and interpreting them cautiously remains appropriate while motivating future breakpoint development for environmental opportunists [41, 42]. Overall, the integrated approach near-complete assembly, phylogenomic placement, comparator-panel–aware gene-content analysis, locus-scale synteny, and curated isolate-linked gene hotspots supports a coherent, hypothesis-generating model in which NG-CE01 distinctiveness reflects remodeling of carbohydrate-foraging neighborhoods, localized innovation within a stabilized GI_3, and chromosomal refinement of permeability/efflux-linked and nutrient-uptake pathways rather than extensive recent acquisition of classical virulence determinants.

## Conclusion

This study provides genome-resolved context for an ocular *Chryseobacterium* lineage and shows that NG-CE01 represents a genomically distinct member of the genus while formal taxonomic description awaits dedicated follow-up. Using comparator-panel–aware gene-content analysis together with synteny-guided locus interpretation, we find that NG-CE01 lacks canonical acquired virulence factors and known acquired AMR determinants by standard screens, yet exhibits structured chromosome-internal innovation: remodeling of discrete carbohydrate-foraging neighborhoods, concentration of isolate-linked functions within a stabilized genomic island (GI_3), and lineage-specific refinement of chromosomal regions consistent with baseline differences in permeability/efflux-associated and nutrient-uptake processes. These findings emphasize that gene organization and neighborhood-level evolution can convey stronger ecological signals than gene inventories alone, and that architecture-aware comparative genomics is a transferable strategy for generating mechanistic hypotheses in specialized host-associated niches beyond virulence- and acquired-resistome–centric models. 

### Limitations and future perspectives

This study is based on a single ocular isolate; therefore, the identified genomic patterns should be interpreted as hypothesis-generating rather than definitive ocular niche–specific determinants, and gene-content accumulation metrics derived from a curated multi-species *Chryseobacterium* comparator panel are reported descriptively rather than as species-level pangenome properties of NG-CE01. Functional links between remodeled CAZyme neighborhoods, GI-associated loci (notably GI_3), and chromosome-encoded permeability/efflux architecture remain inferential, and disk-diffusion results are presented as inhibition-zone diameters given the limited availability of validated breakpoints for *Chryseobacterium* spp. Future work should expand sampling to additional ocular isolates and closely related lineages, incorporate targeted phenotyping under ocular-mimicking constraints (glycan/mucin substrates, antimicrobial peptide or oxidative challenge, and nutrient limitation), and prioritize expression-based or genetic validation of hotspot/GI_3 candidates to connect locus-scale remodeling with measurable fitness and susceptibility variation, thereby strengthening mechanistic inference and informing breakpoint development for this under-characterized opportunist group. 

## Supplementary Information

Supplementary Material 1.

## Data Availability

Raw sequencing data for *Chryseobacterium* sp. NG-CE01 are publicly available in the NCBI Sequence Read Archive under BioProject **PRJNA1415249** (Illumina: **SRR37096161/SRX32042537** ; Nanopore: **SRR37096160/SRX32042536** ): (https://www.ncbi.nlm.nih.gov/bioproject/?term=PRJNA1415249) ; (https://www.ncbi.nlm.nih.gov/sra/SRX32042537%5Baccn); (https://www.ncbi.nlm.nih.gov/sra/SRX32042536%5Baccn). The genome assembly is available in GenBank under accession **JBUXJS000000000** . NG-CE01 is not currently deposited in two independent international culture collections; therefore, culture collection accession numbers are not available. The strain is maintained in the authors’ laboratory and may be provided by the corresponding author upon reasonable request, subject to institutional and material transfer policies.

## Abbreviations

ANI: Average nucleotide identity
dDDH: Digital DNA–DNA hybridization
CAZyme: Carbohydrate-active enzyme
CBM: Carbohydrate-binding module
CE: Carbohydrate esterase
GH: Glycoside hydrolase
GT: Glycosyltransferase
PL: Polysaccharide lyase
GI: Genomic island
ICE: Integrative conjugative element
IS: Insertion sequence
CRISPR: Clustered regularly interspaced short palindromic repeats
FDR: False discovery rate
CLSI: Clinical and Laboratory Standards Institute
EUCAST: European Committee on Antimicrobial Susceptibility Testing
COG: Clusters of Orthologous Groups
ORF: Open reading frame
rRNA: Ribosomal RNA
tRNA: Transfer RNA
T4SS: Type IV secretion system
GC: Guanine - cytosine
