# Supplementary material for: Comparative genomics reveals synteny-guided remodeling of carbohydrate-foraging and intrinsic resistance in an ocular Chryseobacterium isolate, NG-CE01
Source: BMC Microbiol. 2026 Mar 31;26:326. doi: 10.1186/s12866-026-04950-8 (PMC13063519; doi:10.1186/s12866-026-04950-8)
Supplement: Supplementary file 1 — Supplementary Material 1. [file 12866_2026_4950_MOESM1_ESM.docx]

**Supplementary Table S1. Computational tools used in this study**

| **Analysis category** | **Tool** | **Version** |
| --- | --- | --- |
| Sequencing QC | FastQC | 0.12.1 |
| Sequencing QC | MultiQC | 1.9 / 1.19 |
| Long-read QC | NanoPlot | 1.40.2 |
| Read trimming | fastp | 0.20.0 |
| Read filtering / down sampling | Filtlong | 0.2.1 |
| Read down sampling | Seqtk | 1 |
| Genome assembly | Flye | 2.9.3 |
| ONT polishing | Medaka | 1.8 |
| Short-read alignment | BWA | 0.7.17 |
| Short-read polishing | Polypolish | 0.6.0 |
| Assembly quality control | QUAST | 5.2.0 / 5.3.0 |
| Assembly quality control | CheckM2 | 1.0.1 |
| Genome completeness | BUSCO | 5.7.1 |
| Taxonomic purity | Kraken2 | — |
| Genome annotation | Bakta | 1.8.2 |
| Genome annotation | Prokka | 1.13 |
| Functional annotation | eggNOG-mapper | 2.1.8 |
| Taxonomic assignment | GTDB-Tk | 2.3.2 (GTDB R220) |
| ANI estimation | FastANI | 1.34 |
| dDDH | TYGS | Web |
| 16S rRNA analysis | BLASTn | 2.17.0 |
| Pangenome analysis | Panaroo | 1.5.2 |
| Gene–trait association | Scoary | 1.6.16 |
| CAZyme annotation | dbCAN3 | 3.0.6 |
| Genomic islands (primary) | IslandViewer4 | Web |
| Genomic islands (validation) | IslandPick | Local (Docker) |
| Genomic islands (validation) | IslandPath-DIMOB | Local (Docker) |
| Genomic islands (validation) | SIGI-HMM | Local (Docker) |
| Insertion sequences | ISfinder | Web |
| Integrative conjugative elements | ICEberg | 3 |
| Prophages | PHASTER | Web |
| Plasmid detection | PlasmidFinder | — |
| Integron detection | IntegronFinder | — |
| AMR screening | AMRFinderPlus | 4.0.23 |
| AMR / virulence screening | ABRicate | 1.0.1 |
| Virulence database | VFDB | Updated 25-01-2026 |
| CRISPR–Cas detection | MinCED | Galaxy v0.1.5 |
| CRISPR validation | CRT | Galaxy v1.2.0 |
| Synteny analysis | Clinker | — |
| Linear locus visualization | DNAFeaturesViewer | 3.1.5 |
| Circular genome visualization | CGView | 2.0.3 |
| Figure refinement | Proksee | Web |
| Statistical computing | Python | 3.10.0 |
| CAZyme workflows | Python | 3.8.20 |
| Numerical computing | NumPy | 1.24.4 |
| Statistical analysis | SciPy | 1.10.1 |
| Data handling | pandas | 2.2.2 |
| Plotting | Matplotlib | 3.7.5 |

**Supplementary Table S2A. Legend for eggNOG/COG single-letter functional categories used in this study**

| **COG letter** | **Functional category** |
| --- | --- |
| C | Energy production & conversion |
| D | Cell cycle control, cell division, chromosome partitioning |
| E | Amino acid transport & metabolism |
| F | Nucleotide transport & metabolism |
| G | Carbohydrate transport & metabolism |
| H | Coenzyme transport & metabolism |
| I | Lipid transport & metabolism |
| J | Translation, ribosomal structure & biogenesis |
| K | Transcription |
| L | Replication, recombination & repair |
| M | Cell wall/membrane/envelope biogenesis |
| N | Cell motility |
| O | Posttranslational modification, protein turnover, chaperones |
| P | Inorganic ion transport & metabolism |
| Q | Secondary metabolites biosynthesis, transport & catabolism |
| R | General function prediction only |
| S | Function unknown |
| T | Signal transduction mechanisms |
| U | Intracellular trafficking, secretion & vesicular transport |
| V | Defense mechanisms |
| W | Extracellular structures |
| Y | Nuclear structure (rare in bacteria) |
| Z | Cytoskeleton (rare in bacteria) |

**Supplementary Table S2B. Legend for CAZyme classes codes used in this study**

| **Abbreviation** | **Full name** | **Biologically Function** |
| --- | --- | --- |
| GH | Glycoside hydrolases | Enzymes that hydrolyze glycosidic bonds |
| GT | Glycosyltransferases | Enzymes that build glycosidic bonds by transferring sugars |
| CE | Carbohydrate esterases | Remove acetyl/succinyl (and related) esters from carbohydrates |
| PL | Polysaccharide lyases | Cleavage by β-elimination (not hydrolysis) |
| AA | Auxiliary activities | Redox enzymes that assist carbohydrate conversion (often oxidative) |
| CBM | Carbohydrate-binding modules | Non-catalytic binding domains that tether enzymes to substrates |

**Supplementary Table S3. Biochemical and culture characteristics of *Chryseobacterium* NGCE01**

| **Test / Medium** | **Result** |
| --- | --- |
| Catalase | Positive |
| Oxidase | Positive |
| Coagulase | Negative |
| Urease | Negative |
| Indole | Positive |
| Bile esculin | Positive |
| Nitrate reduction | Positive |
| Motility | Negative |
| Mannitol fermentation | Negative |
| Sorbitol fermentation | Negative |
| β-galactosidase | Negative |
| Oxidation of glucose | Positive |
| Fermentation of glucose | Negative |
| TSI | K/K |
| Citrate | Variable (V) |
| Mannitol salt agar | No growth |
| EMB agar | Growth (non-lactose fermenter) |
| MacConkey agar | Growth (non-lactose fermenter) |
| Sorbitol MacConkey agar | Growth (colorless) |
| Mueller–Hinton agar | Yellow (lemon) colonies |
| Trypticase soy agar | Yellow (lemon) colonies |
| Blood agar | Growth |
| Hemolysis | Positive |

**Supplementary Table S4. Disk diffusion inhibition-zone diameters for *Chryseobacterium* sp. NG-CE01.**

| **Antibiotic class** | **Antibiotic** | **Disk code** | **Zone (mm)** |
| --- | --- | --- | --- |
| **β-lactams (penicillins)** | Ampicillin | AMP 10 | 0 |
|  | Amoxicillin–clavulanate | AMC 30 | 17.8 |
| **β-lactams (cephalosporins)** | Cefoxitin | FOX 30 | 22.7 |
|  | Cefotaxime | CTX 30 | 30.6 |
|  | Ceftriaxone | CRO 30 | 14.2 |
|  | Ceftiofur | FUR 30 | 24.6 |
| **β-lactams (monobactam)** | Aztreonam | ATM 30 | 0 |
| **β-lactams (carbapenems)** | Imipenem | IMP 10 | 38.8 |
|  | Meropenem | MEM 10 | 32 |
|  | Ertapenem | ETP 10 | 0 |
| **β-lactam + inhibitor (synergy disk)** | Cefotaxime–clavulanate | CTC 40 | 38.2 |
| **Aminoglycosides** | Gentamicin | CN 10 | 21.2 |
|  | Tobramycin | TOB 10 | 0 |
|  | Neomycin | N 10 | 0 |
| **Fluoroquinolones** | Ciprofloxacin | CIP 5 | 34.2 |
|  | Levofloxacin | LEV 5 | 36.6 |
|  | Enrofloxacin | ENR 5 | 40 |
| **Tetracyclines** | Doxycycline | DO 30 | 25.6 |
|  | Oxytetracycline | T 30 | 18.8 |
| **Phenicols** | Chloramphenicol | C 30 | 27.2 |
|  | Florfenicol | FFC 30 | 33.2 |
| **Folate pathway inhibitors** | Trimethoprim–sulfamethoxazole | SXT 25 | 26.8 |
| **Polymyxins** | Polymyxin B | PB 300 | 0 |
| **Polypeptides** | Bacitracin | B 10 | 0 |

**Supplementary Table S5A. Comparator genome panel used for comparative analyses.**

Assembly accessions, primary sequence IDs, organism/strain labels, and assembly statistics (total length, GC%, and N50) for the 13 reference *Chryseobacterium* genomes included alongside NG-CE01 in the curated comparator panel (N = 14). This table documents comparator selection and dataset characteristics for interspecies comparative analyses.

| **Assembly accession** | **PrimarySeqID** | **Organism (strain/isolate)** | **Total length (bp)** | | **GC (%)** | **N50 (bp)** |
| --- | --- | --- | --- | --- | --- | --- |
| GCA_001013295.1 | LAZY01000001.1 | *C. indologenes (J31)* | | 5,811,735 | 36.88 | 106,549 |
| GCA_001420285.1 | LLYZ01000001.1 | *C. aquaticum (KCTC 12483)* | | 3,812,160 | 33.94 | 465,295 |
| GCA_001593385.1 | LUVZ01000001.1 | *C. cucumeris (GSE06)* | | 5,323,754 | 36.09 | 611,595 |
| GCA_001684965.1 | MAYG01000001.1 | *C. arthrosphaerae (CC-VM-7)* | | 5,043,790 | 38.25 | 3,426,081 |
| GCA_002294525.1 | DBES01000008.1 | *C. gleum (UBA900)* | | 5,200,680 | 36.81 | 805,045 |
| GCA_002899875.1 | PPEH01000001.1 | *C. lactis (NCTC11390)* | | 5,589,501 | 36.08 | 584,344 |
| GCA_002943655.1 | MUGP01000001.1 | *C. shigense (DSM 17126)* | | 4,889,274 | 37.47 | 324,632 |
| GCA_003729955.1 | RJTX01000007.1 | *C. daecheongense (DSM 15235)* | | 3,831,316 | 36.23 | 983,762 |
| GCA_003815775.1 | CP033926.1 | *C. joostei (DSM 16927)* | | 4,890,621 | 35.45 | 4,860,412 |
| GCA_003860485.1 | CP034171.1 | *C. taklimakanense (H4753)* | | 2,626,692 | 40.64 | 2,596,260 |
| GCA_012927265.1 | JABBGF010000001.1 | *C. cheonjiense (RJ-7-14)* | | 4,276,416 | 37.25 | 2,336,728 |
| GCA_014201785.1 | JACHFI010000001.1 | *C. koreense (DSM 25209)* | | 3,178,681 | 40.11 | 192,384 |
| GCA_041913965.1 | JAYSEF010000001.1 | *C. taeanense (ZZ2_ZZA248_bin.41)* | | 3,927,566 | 36.22 | 138,688 |

| **Assembly accession** | **BUSCO C (%)** | **BUSCO D (%)** | **BUSCO F (%)** | **BUSCO M (%)** |
| --- | --- | --- | --- | --- |
| GCA_001013295.1 | 99.2 | 0.8 | 0.8 | 0 |
| GCA_001420285.1 | 100 | 0 | 0 | 0 |
| GCA_001593385.1 | 99.2 | 0 | 0.8 | 0 |
| GCA_001684965.1 | 99.2 | 0 | 0.8 | 0 |
| GCA_002294525.1 | 99.2 | 0.8 | 0.8 | 0 |
| GCA_002899875.1 | 99.2 | 0.8 | 0.8 | 0 |
| GCA_002943655.1 | 99.2 | 0.8 | 0.8 | 0 |
| GCA_003729955.1 | 100 | 0 | 0 | 0 |
| GCA_003815775.1 | 98.4 | 0 | 1.6 | 0 |
| GCA_003860485.1 | 91.1 | 0 | 6.5 | 2.4 |
| GCA_012927265.1 | 98.4 | 0 | 1.6 | 0 |
| GCA_014201785.1 | 99.2 | 0 | 0.8 | 0 |
| GCA_041913965.1 | 95.2 | 0 | 1.6 | 3.2 |

**Supplementary Table S5B. Completeness assessment for comparator genomes (BUSCO).**

BUSCO completeness metrics (Complete, Duplicated, Fragmented, Missing; %) for the reference genomes listed in Supplementary Table S5A. These values provide quality context for the curated comparator panel used in comparative gene-content and locus-scale analyses.

**Supplementary Table S6. Key branch lengths and bootstrap support values for internal nodes supporting the phylogenomic placement of *Chryseobacterium* strain NG-CE01 inferred from concatenated conserved marker genes. Branch lengths represent substitutions per site; bootstrap values ≥70% are shown.**

| **Node ID** | **Relationship** | **Descendant taxa (split)** | **Branch length (subs/site)** | **Bootstrap (%)** |
| --- | --- | --- | --- | --- |
| N4 | Context clade | *C. aquaticum + C. shigense — (C. daecheongense + downstream taxa including NG-CE01 clade)* | 0.01396 | 90 |
| N6 | NG-CE01-containing subclade | *(C. daecheongense + C. taklimakanense + C. koreense) — (C. cheonjiense + NG-CE01 + C. taeanense)* | 0.00812 | 82 |
| N9 | NG-CE01 vs nearest outgroup | *(C. cheonjiense + NG-CE01) — C. taeanense* | 0.02188 | 100 |
| **N10** | **Focal sister relationship** | ***C. cheonjiense — Chryseobacterium strain NG-CE01*** | **0.0134** | **100** |
| N7 | Supporting sister split | *C. daecheongense — (C. taklimakanense + C. koreense)* | 0.01387 | 93 |

**Supplementary Table S7. Pairwise FastANI comparisons between strain NG-CE01 and reference *Chryseobacterium* genomes.**

| **Query genome** | **Reference genome (accession)** | **Reference species/strain** | **FastANI (%)** | **AF (Aligned/Total)** | **AF (%)** | **Aligned fragments** | **Total fragments** |
| --- | --- | --- | --- | --- | --- | --- | --- |
| **NG-CE01** | NG-CE01 (self) | *Chryseobacterium sp. NG-CE01* | 100 | 1443/1443 | 100 | 1443 | 1443 |
| **NG-CE01** | **GCA_012927265.1** | ***C. cheonjiense strain RJ-7-14*** | **93.1206** | **1213/1443** | **84.06** | **1213** | **1443** |
| **NG-CE01** | GCA_041913965.1 | *C. taeanense isolate ZZ2_ZZA248_bin.41 (MAG)* | 82.549 | 873/1443 | 60.5 | 873 | 1443 |
| **NG-CE01** | GCA_001593385.1 | *C. cucumeris strain GSE06* | 79.9544 | 657/1443 | 45.53 | 657 | 1443 |
| **NG-CE01** | GCA_002294525.1 | *C. gleum isolate UBA900 (MAG)* | 79.8511 | 696/1443 | 48.23 | 696 | 1443 |
| **NG-CE01** | GCA_002943655.1 | *C. shigense strain DSM 17126* | 79.7376 | 593/1443 | 41.09 | 593 | 1443 |
| **NG-CE01** | GCA_001684965.1 | *C. arthrosphaerae strain CC-VM-7* | 79.7201 | 626/1443 | 43.38 | 626 | 1443 |
| **NG-CE01** | GCA_003815775.1 | *C. joostei strain DSM 16927 (chromosome)* | 79.7167 | 575/1443 | 39.85 | 575 | 1443 |
| **NG-CE01** | GCA_003729955.1 | *C. daecheongense strain DSM 15235* | 79.6934 | 614/1443 | 42.55 | 614 | 1443 |
| **NG-CE01** | GCA_002899875.1 | *C. lactis strain NCTC11390* | 79.6304 | 580/1443 | 40.19 | 580 | 1443 |
| **NG-CE01** | GCA_001013295.1 | *C. indologenes strain J31* | 79.498 | 632/1443 | 43.8 | 632 | 1443 |
| **NG-CE01** | GCA_001420285.1 | *C. aquaticum strain KCTC 12483* | 79.1442 | 601/1443 | 41.65 | 601 | 1443 |

**Supplementary Table S8.** **BLASTn results for the 16S rRNA gene extracted from strain NG-CE01.** The top matches correspond to *Chryseobacterium cheonjiense*, showing high sequence identity but incomplete query coverage, consistent with genus-level identification and limited species-level resolution of 16S rRNA within the genus *Chryseobacterium*.

| **Query sequence** | **Description (Top BLAST hit)** | **Scientific name** | **Query cover (%)** | **Percent identity (%)** | **E value** | **Accession number** |
| --- | --- | --- | --- | --- | --- | --- |
| NG-CE01 16S rRNA | *Chryseobacterium cheonjiense* strain RJ-7-14 16S ribosomal RNA, partial sequence | *Chryseobacterium cheonjiense* | 95 | 99.10 | 0 | NR_180687.1 |
| NG-CE01 16S rRNA | *Chryseobacterium cheonjiense* strain RJ-7-14 16S ribosomal RNA, partial sequence | *Chryseobacterium cheonjiense* | 96 | 99.04 | 0 | NR_180931.1 |

**Supplementary Table S9. Gene-cluster accumulation across the curated multi-species comparator panel (500 permutations).**

| **Genomes_n** | **Pangenome_Mean** | **Pangenome_SD** | **New_Genes_Mean** | **New_Genes_SD** |
| --- | --- | --- | --- | --- |
| **1** | 3415.536 | 1184.695 | 3415.536 | 1184.695 |
| **2** | 5813.48 | 1045.7 | 2397.944 | 1042.247 |
| **3** | 7729.334 | 1019.195 | 1915.854 | 869.637 |
| **4** | 9477.642 | 1002.019 | 1748.308 | 736.363 |
| **5** | 11062.32 | 977.742 | 1584.678 | 640.734 |
| **6** | 12498.52 | 956.991 | 1436.204 | 559.959 |
| **7** | 13930.12 | 964.471 | 1431.596 | 554.355 |
| **8** | 15294.49 | 891.5 | 1364.372 | 542.645 |
| **9** | 16565.87 | 876.25 | 1271.38 | 505.594 |
| **10** | 17788.94 | 830.918 | 1223.066 | 507.396 |
| **11** | 18996.14 | 762.962 | 1207.2 | 504.133 |
| **12** | 20173.17 | 670.395 | 1177.03 | 537.927 |
| **13** | 21309.68 | 503.16 | 1136.516 | 488.286 |
| **14** | 22436 | 0 | 1126.316 | 503.16 |

**Supplementary Table S10A. Heaps’ α for gene-cluster accumulation across nested comparator-panel sizes (interpretation limited at interspecies scale).**

| **Genomes_n** | **Heaps_alpha** | **alpha_CI_low** | **alpha_CI_high** | **Pangenome_total** | **Pangenome_SD** | **New_genes_last_mean** | **New_genes_last_SD** |
| --- | --- | --- | --- | --- | --- | --- | --- |
| **8** | 0.7085 | 0.5357 | 0.9967 | 15343.97 | 888.913 | 1340.348 | 559.708 |
| **10** | 0.7006 | 0.5588 | 0.9189 | 17769.41 | 848.529 | 1234.518 | 506.437 |
| **12** | 0.6938 | 0.5612 | 0.8768 | 20201.87 | 693.367 | 1198.836 | 499.335 |
| **14** | 0.6897 | 0.5824 | 0.8473 | 22436 | 0 | 1119.75 | 507.035 |

**Supplementary Table S10B. Panaroo summary statistics for ANI-selected closest-only comparator subset (N = 8).** Summary gene-cluster frequency statistics from Panaroo v1.5.2 run on an ANI-selected closest-only subset (NG-CE01 plus the seven highest-ANI reference genomes; N = 8). Panaroo was run in --clean-mode sensitive with --core_threshold 0.99 (core defined as present in 99–100% of genomes; for N = 8 this corresponds to 8/8 genomes). Values are reported to document panel-composition sensitivity of core/accessory partitioning.

| **Category (Panaroo output)** | **Definition (as reported by Panaroo)** | **Gene clusters (count)** |
| --- | --- | --- |
| Core genes | 99% ≤ strains ≤ 100% | 523 |
| Soft core genes | 95% ≤ strains < 99% | 0 |
| Shell genes | 15% ≤ strains < 95% | 5,493 |
| Cloud genes | 0% ≤ strains < 15% | 12,366 |
| Total genes | 0% ≤ strains ≤ 100% | 18,382 |

**Supplementary Table S11.Genomic island–associated unique genes identified relative to the comparator panel (Panaroo-defined isolate-specific clusters) and mapped to UGIs**. The table summarizes the genomic coordinates (start, end, and strand), locus tags, Prokka-assigned gene names, functional product annotations, and corresponding eggNOG COG categories for each unique gene located within the predicted genomic islands (UGIs). UGI identifiers indicate the genomic island number and its genomic span, facilitating mapping of functionally enriched gene clusters across the genome.

| **UGI** | **Start** | **End** | **Strand** | **Locus_tag** | **Gene Name** | **Product_annotation** | **COG** |
| --- | --- | --- | --- | --- | --- | --- | --- |
| UGI00_24649-44296 | 24649 | 26646 | + | HEDAFHDN_00026 | ycf3 | Photosystem I assembly protein Ycf3 | T |
| UGI00_24649-44296 | 34673 | 35311 | + | HEDAFHDN_00039 | rhaS_1 | HTH-type transcriptional activator RhaS | K |
| UGI00_24649-44296 | 39188 | 41374 | + | HEDAFHDN_00044 | lagD_1 | Lactococcin-G-processing and transport ATP-binding protein LagD | V |
| UGI00_24649-44296 | 41384 | 44296 | + | HEDAFHDN_00045 | btuD_1 | Vitamin B12 import ATP-binding protein BtuD | V |
| UGI01_217481-218119 | 217481 | 218119 | − | HEDAFHDN_00218 |  | IS110 family transposase ISCaa14 | L |
| UGI02_270305-285160 | 270305 | 271192 | − | HEDAFHDN_00267 | gnu | N-acetyl-α-D-glucosaminyl-diphospho-ditrans, octacis-undecaprenol 4-epimerase | GM |
| UGI02_270305-285160 | 280261 | 281412 | − | HEDAFHDN_00276 | wecB | UDP-N-acetylglucosamine 2-epimerase | M |
| UGI02_270305-285160 | 282791 | 285160 | − | HEDAFHDN_00278 | ptk | Tyrosine-protein kinase Ptk | DM |
| UGI03_585392-586501 | 585392 | 586501 | + | HEDAFHDN_00564 | resA_2 | Thiol-disulfide oxidoreductase ResA | O |
| UGI04_813263-838080 | 813263 | 814723 | − | HEDAFHDN_00810 | - | SusD-like protein P2 | S |
| UGI04_813263-838080 | 814735 | 817593 | − | HEDAFHDN_00811 | - | TonB-dependent receptor P3 | P |
| UGI04_813263-838080 | 821694 | 824609 | − | HEDAFHDN_00814 | susC_1 | TonB-dependent receptor SusC | P |
| UGI04_813263-838080 | 834321 | 835571 | + | HEDAFHDN_00823 | btuD_4 | Vitamin B12 import ATP-binding protein BtuD | GM |
| UGI04_813263-838080 | 836364 | 837446 | + | HEDAFHDN_00825 | vioA | dTDP-4-amino-4,6-dideoxy-D-glucose transaminase | E |
| UGI04_813263-838080 | 837448 | 838080 | + | HEDAFHDN_00826 | lpxD_3 | UDP-3-O-(3-hydroxymyristoyl)glucosamine N-acyltransferase | S |
| UGI05_1077309-1095274 | 1077309 | 1077818 | + | HEDAFHDN_01054 | ymdB | O-acetyl-ADP-ribose deacetylase | S |
| UGI05_1077309-1095274 | 1077949 | 1078635 | + | HEDAFHDN_01055 | cobB | NAD-dependent protein deacylase | K |
| UGI05_1077309-1095274 | 1078635 | 1079597 | + | HEDAFHDN_01056 | tri1 | ADP-ribosylarginine hydrolase Tri1 | O |
| UGI05_1077309-1095274 | 1079578 | 1080123 | + | HEDAFHDN_01057 | kptA | Putative RNA 2′-phosphotransferase | J |
| UGI05_1077309-1095274 | 1086084 | 1087496 | − | HEDAFHDN_01064 | sasA_8 | Adaptive-response sensory kinase SasA | T |
| UGI05_1077309-1095274 | 1087501 | 1088208 | − | HEDAFHDN_01065 | cusR_1 | Transcriptional regulatory protein CusR | T |
| UGI05_1077309-1095274 | 1088910 | 1089845 | − | HEDAFHDN_01066 | putB | Proline dehydrogenase 2 | E |
| UGI05_1077309-1095274 | 1091198 | 1094236 | − | HEDAFHDN_01069 | mdtB | Multidrug resistance protein MdtB | V |
| UGI05_1077309-1095274 | 1094243 | 1095274 | − | HEDAFHDN_01070 | mexA | Multidrug resistance protein MexA | M |
| UGI06_1188336-1191673 | 1188336 | 1189514 | + | HEDAFHDN_01168 | hlyD | Hemolysin secretion protein D | M |
| UGI06_1188336-1191673 | 1189514 | 1191673 | + | HEDAFHDN_01169 | lagD_3 | Lactococcin-G-processing and transport ATP-binding protein LagD | V |
| UGI07_1303723-1305063 | 1303723 | 1305063 | − | HEDAFHDN_01292 | hcf136 | Ycf48-like protein | S |
| UGI08_1697243-1698232 | 1697243 | 1698232 | + | HEDAFHDN_01658 | - | IS110 family transposase ISCaa14 | L |
| UGI09_1825291-1825995 | 1825291 | 1825995 | + | HEDAFHDN_01777 | - | IS1182 family transposase ISMsp1 | L |
| UGI10_2019033-2019725 | 2019033 | 2019725 | − | HEDAFHDN_01949 | perB | GDP-perosamine N-acetyltransferase | H |
| UGI11_2329943-2335785 | 2329943 | 2330809 | + | HEDAFHDN_02249 | kfoC_2 | Chondroitin synthase | S |
| UGI11_2329943-2335785 | 2330799 | 2331473 | + | HEDAFHDN_02250 | cusR_2 | Transcriptional regulatory protein CusR | T |
| UGI11_2329943-2335785 | 2331485 | 2332831 | + | HEDAFHDN_02251 | arlS | Signal transduction histidine-protein kinase ArlS | T |
| UGI11_2329943-2335785 | 2333503 | 2335785 | − | HEDAFHDN_02253 | yccS | Inner membrane protein YccS | S |
| UGI12_2388364-2389341 | 2388364 | 2389341 | + | HEDAFHDN_02294 | - | IS110 family transposase ISCaa7 | L |
| UGI13_2444006-2471066 | 2444006 | 2446198 | − | HEDAFHDN_02338 | lagD_4 | Lactococcin-G-processing and transport ATP-binding protein LagD | V |
| UGI13_2444006-2471066 | 2456132 | 2458321 | + | HEDAFHDN_02348 | f1pep1_2 | Prolyl endopeptidase | E |
| UGI13_2444006-2471066 | 2458322 | 2460514 | + | HEDAFHDN_02349 | nisB_1 | Nisin biosynthesis protein NisB | S |
| UGI13_2444006-2471066 | 2460517 | 2461377 | + | HEDAFHDN_02350 | nisB_2 | Nisin biosynthesis protein NisB | S |
| UGI13_2444006-2471066 | 2470356 | 2471066 | + | HEDAFHDN_02358 | magA_2 | Iron transporter MagA | P |
| UGI14_2636176-2637201 | 2636176 | 2637201 | − | HEDAFHDN_02502 | - | 4-O-β-D-mannosyl-D-glucose phosphorylase | G |
| UGI15_2667302-2668291 | 2667302 | 2668291 | + | HEDAFHDN_02532 | - | IS110 family transposase ISCaa14 | L |
| UGI16_2809085-2810236 | 2809085 | 2810236 | + | HEDAFHDN_02651 | - | Putative zinc-binding alcohol dehydrogenase | E |
| UGI17_2834426-2837533 | 2834426 | 2837533 | + | HEDAFHDN_02675 | hsdR_1 | Type I restriction enzyme EcoR124II R protein | L |
| UGI18_3059924-3061645 | 3059924 | 3061645 | − | HEDAFHDN_02887 | nfdA | N-substituted formamide deformylase | S |
| UGI19_3353478-3386632 | 3353478 | 3354698 | + | HEDAFHDN_03167 | xerC_4 | Tyrosine recombinase XerC | L |
| UGI19_3353478-3386632 | 3359040 | 3363470 | + | HEDAFHDN_03170 | recD | RecBCD enzyme subunit RecD | L |
| UGI19_3353478-3386632 | 3366648 | 3368093 | + | HEDAFHDN_03176 | norR_2 | Anaerobic nitric oxide reductase regulator NorR | T |
| UGI19_3353478-3386632 | 3376201 | 3377247 | − | HEDAFHDN_03182 | ampH | D-alanyl-D-alanine carboxypeptidase AmpH | V |
| UGI19_3353478-3386632 | 3385064 | 3386632 | + | HEDAFHDN_03192 | prmC_2 | Release factor glutamine methyltransferase | L |
| UGI20_3398240-3399022 | 3398240 | 3399022 | − | HEDAFHDN_03199 | ynbD | Putative protein YnbD | IT |
| UGI21_3512157-3515024 | 3512157 | 3515024 | − | HEDAFHDN_03288 | - | TonB-dependent receptor P3 | H |
| UGI22_3529307-3530709 | 3529307 | 3529822 | − | HEDAFHDN_03301 | dapH_4 | Tetrahydrodipicolinate N-acetyltransferase | S |
| UGI22_3529307-3530709 | 3529819 | 3530709 | − | HEDAFHDN_03302 | arnC_4 | Undecaprenyl-phosphate transferase | M |
| UGI23_3579176-3593022 | 3579176 | 3581161 | − | HEDAFHDN_03350 | yjcS | Putative alkyl/aryl-sulfatase | Q |
| UGI23_3579176-3593022 | 3582738 | 3583808 | − | HEDAFHDN_03353 | wecA_2 | Undecaprenyl-phosphate glycosyltransferase | M |
| UGI23_3579176-3593022 | 3586256 | 3587488 | − | HEDAFHDN_03356 | mshA_3 | D-inositol-3-phosphate glycosyltransferase | M |
| UGI23_3579176-3593022 | 3587436 | 3588176 | − | HEDAFHDN_03357 | - | Putative acetyltransferase | S |
| UGI23_3579176-3593022 | 3591907 | 3593022 | − | HEDAFHDN_03360 | mshA_4 | D-inositol-3-phosphate glycosyltransferase | M |
| UGI24_3642842-3651366 | 3642842 | 3644290 | − | HEDAFHDN_03389 | cusC | Cation efflux system protein CusC | MU |
| UGI24_3642842-3651366 | 3644277 | 3647393 | − | HEDAFHDN_03390 | bepE_4 | Efflux pump membrane transporter BepE | V |
| UGI24_3642842-3651366 | 3647411 | 3648499 | − | HEDAFHDN_03391 | ttgG | Toluene efflux pump linker protein TtgG | M |
| UGI24_3642842-3651366 | 3650260 | 3651366 | + | HEDAFHDN_03394 | rfbD_2 | UDP-galactopyranose mutase | M |
| UGI25_3665798-3668451 | 3665798 | 3666712 | − | HEDAFHDN_03410 | rhaR_4 | HTH-type transcriptional activator RhaR | K |
| UGI25_3665798-3668451 | 3666730 | 3667467 | − | HEDAFHDN_03411 | - | Putative oxidoreductase | S |
| UGI25_3665798-3668451 | 3667474 | 3668451 | − | HEDAFHDN_03412 | iolS_2 | Aldo-keto reductase IolS | C |
| UGI26_3682454-3704765 | 3682454 | 3683674 | + | HEDAFHDN_03427 | nhaP2_3 | K⁺/H⁺ antiporter NhaP2 | P |
| UGI26_3682454-3704765 | 3683736 | 3684824 | + | HEDAFHDN_03428 | - | Putative transport protein | D |
| UGI26_3682454-3704765 | 3688340 | 3689167 | + | HEDAFHDN_03434 | yflN | Putative metallo-hydrolase YflN | S |
| UGI26_3682454-3704765 | 3693604 | 3695796 | − | HEDAFHDN_03439 | lagD_5 | Lactococcin-G-processing and transport ATP-binding protein LagD | V |
| UGI26_3682454-3704765 | 3701937 | 3703259 | − | HEDAFHDN_03445 | gntT | High-affinity gluconate transporter | EG |
| UGI26_3682454-3704765 | 3703275 | 3704765 | − | HEDAFHDN_03446 | xylB_2 | Xylulose kinase | G |
| UGI27_3751878-3764001 | 3751878 | 3752903 | + | HEDAFHDN_03489 | flp | Protein Flp | V |
| UGI27_3751878-3764001 | 3755158 | 3756459 | + | HEDAFHDN_03492 | pbpE_2 | Penicillin-binding protein 4 | V |
| UGI27_3751878-3764001 | 3757381 | 3758835 | + | HEDAFHDN_03494 | dap_4 | D-aminopeptidase | V |
| UGI27_3751878-3764001 | 3760552 | 3761226 | + | HEDAFHDN_03497 | - | 4-sulfomuconolactone hydrolase | S |
| UGI27_3751878-3764001 | 3762550 | 3764001 | − | HEDAFHDN_03499 | dap_5 | D-aminopeptidase | V |
| UGI28_3798655-3804379 | 3798655 | 3799791 | + | HEDAFHDN_03534 | iscS_2 | Cysteine desulfurase IscS | E |
| UGI28_3798655-3804379 | 3802241 | 3804379 | + | HEDAFHDN_03537 | smc | Chromosome partition protein Smc | D |
| UGI29_3814746-3820038 | 3814746 | 3815603 | + | HEDAFHDN_03549 | GlxR | 2-hydroxy-3-oxopropionate reductase | I |
| UGI29_3814746-3820038 | 3818502 | 3819551 | − | HEDAFHDN_03554 | - | 2-haloacrylate reductase | C |
| UGI29_3814746-3820038 | 3819682 | 3820038 | + | HEDAFHDN_03555 | hxlR | HTH-type transcriptional activator HxlR | K |
| UGI30_3846197-3847075 | 3846197 | 3847075 | + | HEDAFHDN_03576 | - | IS3 family transposase ISLbl1 | L |
| UGI31_3928957-3929946 | 3928957 | 3929946 | − | HEDAFHDN_03648 | - | IS110 family transposase ISCaa14 | L |
| UGI32_3987129-3990008 | 3987129 | 3990008 | − | HEDAFHDN_03700 | susC-4 | TonB-dependent receptor SusC | P |
| UGI33_4103734-4107163 | 4103734 | 4104966 | + | HEDAFHDN_03810 | menH_4 | Succinyl-hydroxycyclohexadiene synthase | S |
| UGI33_4103734-4107163 | 4105007 | 4107163 | + | HEDAFHDN_03811 | nisB_3 | Nisin biosynthesis protein NisB | S |
| UGI34_4126182-4127159 | 4126182 | 4127159 | − | HEDAFHDN_03832 | - | IS110 family transposase ISCaa7 | L |
| UGI35_4187963-4193938 | 4187963 | 4188940 | + | HEDAFHDN_03882 | - | IS110 family transposase ISCaa7 | L |
| UGI35_4187963-4193938 | 4192961 | 4193938 | + | HEDAFHDN_03886 | - | IS110 family transposase ISCaa7 | L |

**Supplementary Table S12.COG functional category enrichment analysis of the 90 unique genomic island–associated genes compared with the whole-genome background.** The table reports the observed number of genes within each COG category among the unique gene set (k in 90), genome-wide counts (Genome K), expected counts under random distribution, fold enrichment, and statistical significance based on Fisher’s exact test with Benjamini–Hochberg false discovery rate (FDR) correction. Categories with FDR < 0.05 were considered significantly enriched or depleted relative to the genome background.

| **COG** | **k in 90** | **Genome K** | **Expected in 90** | **Fold enrichment** | **p-value** | **FDR (BH)** | **Significance (FDR<0.05)** |
| --- | --- | --- | --- | --- | --- | --- | --- |
| **V** | **12** | **78** | **2.42** | **4.97** | **3.00E-06** | **5.80E-05** | **Significant** |
| **L** | **14** | **174** | **5.39** | **2.6** | **7.40E-04** | **0.007** | **Significant** |
| **S** | **16** | **880** | **27.26** | **0.59** | **0.0073** | **0.0463** | **Significant (depleted)** |
| T | 6 | 102 | 3.16 | 1.9 | 0.132 | 0.502 | Non-Significant |
| D | 2 | 26 | 0.81 | 2.48 | 0.192 | 0.607 | Non-Significant |
| J | 1 | 152 | 4.71 | 0.21 | 0.088 | 0.42 | Non-Significant |
| M | 9 | 278 | 8.61 | 1.04 | 0.855 | 1.016 | Non-Significant |
| P | 5 | 147 | 4.55 | 1.1 | 0.805 | 1.02 | Non-Significant |
| E | 5 | 176 | 5.45 | 0.92 | 1 | 1 | Non-Significant |
| K | 4 | 197 | 6.1 | 0.66 | 0.521 | 0.991 | Non-Significant |
| O | 2 | 115 | 3.56 | 0.56 | 0.583 | 0.922 | Non-Significant |
| H | 2 | 99 | 3.07 | 0.65 | 0.769 | 1.043 | Non-Significant |
| G | 2 | 136 | 4.21 | 0.47 | 0.441 | 0.93 | Non-Significant |
| Q | 1 | 27 | 0.84 | 1.2 | 0.574 | 0.992 | Non-Significant |
| I | 1 | 98 | 3.04 | 0.33 | 0.369 | 1.003 | Non-Significant |
| C | 2 | 124 | 3.84 | 0.52 | 0.59 | 0.863 | Non-Significant |
| F | 0 | 57 | 1.77 | 0 | 0.422 | 1.002 | Non-Significant |
| N | 0 | 9 | 0.28 | 0 | 1 | 1.056 | Non-Significant |
| U | 0 | 30 | 0.93 | 0 | 1 | 1.118 | Non-Significant |

**Supplementary Table S13. CAZyme class composition across NG-CE01 and 13 reference *Chryseobacterium* genomes.** Values are shown as **count (percentage of total CAZymes per genome)** for each class: glycoside hydrolases (GH), glycosyltransferases (GT), carbohydrate esterases (CE), auxiliary activities (AA), polysaccharide lyases (PL), and carbohydrate-binding modules (CBM).

| **Genome accession** | **Species** | **GH** | **GT** | **CE** | **AA** | **PL** | **CBM** |
| --- | --- | --- | --- | --- | --- | --- | --- |
| **NGCE01** | *Chryseobacterium* sp. *NG-CE01* | 417 (77.9%) | 66 (12.3%) | 22 (4.1%) | 2  (0.4%) | 17 (3.2%) | 11 (2.1%) |
| **GCA_001013295.1** | *Chryseobacterium indologenes* | 206 (67.3%) | 59 (19.3%) | 20 (6.5%) | 13 (4.2%) | 5  (1.6%) | 3  (1.0%) |
| **GCA_001420285.1** | *Chryseobacterium aquaticum* | 212 (67.1%) | 57 (18.0%) | 17 (5.4%) | 10 (3.2%) | 15 (4.7%) | 5  (1.6%) |
| **GCA_001593385.1** | *Chryseobacterium cucumeris* | 346 (76.9%) | 62 (13.8%) | 20 (4.4%) | 14 (3.1%) | 0  (0.0%) | 8  (1.8%) |
| **GCA_001684965.1** | *Chryseobacterium arthrosphaerae* | 306 (77.1%) | 52 (13.1%) | 24 (6.0%) | 7  (1.8%) | 0  (0.0%) | 8  (2.0%) |
| **GCA_002294525.1** | *Chryseobacterium gleum* | 374 (78.1%) | 58 (12.1%) | 21 (4.4%) | 13 (2.7%) | 5  (1.0%) | 8  (1.7%) |
| **GCA_002899875.1** | *Chryseobacterium lactis* | 194 (64.2%) | 64 (21.2%) | 21 (7.0%) | 15 (5.0%) | 5  (1.7%) | 3  (1.0%) |
| **GCA_002943655.1** | *Chryseobacterium shigense* | 200 (63.7%) | 60 (19.1%) | 24 (7.6%) | 12 (3.8%) | 12 (3.8%) | 6  (1.9%) |
| **GCA_003729955.1** | *Chryseobacterium daecheongense* | 388 (81.3%) | 58 (12.2%) | 15 (3.1%) | 5  (1.0%) | 0  (0.0%) | 11 (2.3%) |
| **GCA_003815775.1** | *Chryseobacterium joostei* | 165 (64.2%) | 67 (26.1%) | 16 (6.2%) | 7  (2.7%) | 0  (0.0%) | 2  (0.8%) |
| **GCA_003860485.1** | *Chryseobacterium taklimakanense* | 148 (68.5%) | 46 (21.3%) | 12 (5.6%) | 8  (3.7%) | 0  (0.0%) | 2  (0.9%) |
| **GCA_012927265.1** | *Chryseobacterium cheonjiense* | 442 (78.8%) | 61 (10.9%) | 24 (4.3%) | 6  (1.1%) | 16 (2.9%) | 12 (2.1%) |
| **GCA_014201785.1** | *Chryseobacterium koreense* | 133 (55.9%) | 67 (28.2%) | 13 (5.5%) | 4  (1.7%) | 17 (7.1%) | 4  (1.7%) |
| **GCA_041913965.1** | *Chryseobacterium taeanense* | 381 (76.5%) | 58 (11.6%) | 25 (5.0%) | 6  (1.2%) | 16 (3.2%) | 12 (2.4%) |

**Supplementary Table S14. Rare CAZyme families in NGCE01 relative to 13 reference *Chryseobacterium* genomes.** Rare families are those detected in NGCE01 but present in **≤2/13** reference genomes. CAZyme family presence was called using dbCAN HMMER thresholds (i-Evalue ≤ 1e−15 and HMM coverage ≥ 0.35); low-confidence partial matches were excluded from family counts

| **Family** | **NGCE01_count** | **Ref_presence_N** | **Ref_total** | **Reference genomes with family (accession; species)** |
| --- | --- | --- | --- | --- |
| AA12 | 2 | 2 | 13 | GCA_002943655.1 (*Chryseobacterium shigense*); GCA_012927265.1 (*Chryseobacterium cheonjiense*) |
| GH117 | 1 | 1 | 13 | GCA_012927265.1 (*Chryseobacterium cheonjiense*) |
| GT90 | 1 | 1 | 13 | GCA_041913965.1 (*Chryseobacterium taeanense*) |
| GH1 | 1 | 2 | 13 | GCA_003860485.1 (*Chryseobacterium taklimakanense*); GCA_012927265.1 (*Chryseobacterium cheonjiense*) |
| GT89 | 1 | 2 | 13 | GCA_003815775.1 (*Chryseobacterium joostei*); GCA_012927265.1 (*Chryseobacterium cheonjiense*) |

**Supplementary Table S15. NG-CE01–unique multi-domain CAZyme genes within the Unique90 (non-hypothetical) gene set.** Multi-domain CAZyme proteins among NG-CE01 genes that are unique relative to 13 reference *Chryseobacterium* genomes (Unique90 set). “Architecture” summarizes CAZyme HMM composition per protein. “Domain evidence” reports iE-value and coverage ranges across detected domains.

| **Locus tag** | **Gene name** | **Product annotation** | **COG** | **UGI** | **CAZyme architecture** | **# domains** | **Domain evidence (iE range; cov range)** |
| --- | --- | --- | --- | --- | --- | --- | --- |
| HEDAFHDN_02249 | kfoC_2 | Chondroitin synthase | S | UGI11 | GT2 + GT2 | 2 | 2.4e−32–3.0e−20; 0.53–0.89 |
| HEDAFHDN_02502 | — | 4-O-β-D-mannosyl-D-glucose phosphorylase | G | UGI14 | GH130 ×15 (subfamily-rich) | 15 | 1.8e−173–4.4e−16; 0.38–1.11 |
| HEDAFHDN_03302 | arnC_4 | Undecaprenyl-phosphate 4-deoxy-4-formamido-L-arabinose transferase | M | UGI22 | GT2 + GT2 | 2 | 4.7e−32–3.3e−17; 0.36–0.65 |
| HEDAFHDN_03360 | mshA_4 | D-inositol-3-phosphate glycosyltransferase | M | UGI23 | GT4 + GT5 | 2 | 4.8e−30–7.5e−16; 0.37–0.91 |

Multi-domain CAZymes were defined as proteins with ≥2 CAZyme HMM matches (dbCAN/CAZy HMM library) passing the applied HMMER significance criteria (domain iE-value and coverage thresholds). Only genes classified as NG-CE01–unique within the Unique90 (non-hypothetical) set are shown.

**Supplementary Table S16. Quantitative synteny fingerprint summary for three focal carbohydrate-utilization blocks in NGCE01.** For each block (CBM6–GH5 β-glucan/cellulose-like module, CBM48–GH13 α-glucan module, and GH43 hemicellulose-processing super-block), we report (i) reference prevalence across the 13-genome panel (N/13), (ii) the number of NGCE01 orthology/homolog groups recovered in the clinker-derived tight locus window, and (iii) the range of pairwise Jaccard similarity values computed between each reference locus and the NGCE01 locus based on shared homolog-group membership. The most divergent and most similar reference loci (and second-ranked cases) are listed with their corresponding J values, highlighting locus-level remodeling intensity and clade-restricted conservation relative to NGCE01.

| **Block** | **Ref prevalence (N/13)** | **NGCE01 homolog groups** | **Jaccard range vs NGCE01** | **Most divergent (J)** | **2nd divergent (J)** | **Most similar (J)** | **2nd similar (J)** |
| --- | --- | --- | --- | --- | --- | --- | --- |
| CBM6–GH5 (β-glucan/cellulose-like) | 9/13 | 16 | 0.286–1.000 | *Chryseobacterium indologenes* (0.286) | *Chryseobacterium aquaticum* (0.286) | *Chryseobacterium cheonjiense* (1.000) | *Chryseobacterium taeanense* (MAG) (0.938) |
| CBM48–GH13 (α-glucan) | 13/13 | 18 | 0.111–1.000 | *Chryseobacterium koreense* (0.111) | *Chryseobacterium taklimakanense* (0.444) | *Chryseobacterium cheonjiense* (1.000) | *Chryseobacterium taeanense* (MAG) (0.900) |
| GH43 super-block (hemicellulose) | 6/13 | 24 | 0.562–1.000 | *Chryseobacterium arthrosphaerae* (0.562) | *Chryseobacterium cucumeris* (0.594) | *Chryseobacterium cheonjiense* (1.000) | *Chryseobacterium taeanense* (MAG) (0.778) |

**Supplementary Table S17. Genomic islands in NG-CE01: coordinates, size, gene content, mobility, and Unique90 overlap**

| **GI** | **Contig** | **Start** | **End** | **Length (bp)** | **Genes total** | **Hypothetical (n)** | **Hypothetical (%)** | **Mobility-associated genes (n)** | **Unique90 genes inside GI (n/90)** |
| --- | --- | --- | --- | --- | --- | --- | --- | --- | --- |
| GI_1 | edge_1 | 437,180 | 461,873 | 24,694 | 26 | 23 | 88.5 | 1 | 0 |
| GI_2 | edge_1 | 1,693,707 | 1,709,594 | 15,888 | 13 | 5 | 38.5 | 2 | 1 |
| GI_3 | edge_1 | 3,811,259 | 3,879,037 | 67,779 | 57 | 46 | 80.7 | 4 | 4 |

**Supplementary Table S18.Composition metrics of predicted genomic islands on edge_1**

| **Genomic island** | **GC content (%)** | **Background GC (%)** | **ΔGC (GI − BG)** | **GC skew** |
| --- | --- | --- | --- | --- |
| GI_1 | 30.09 | 37.43 | −7.34 | −0.039 |
| GI_2 | 37.97 | 37.43 | 0.54 | 0.104 |
| GI_3 | 36.69 | 37.43 | −0.74 | −0.047 |

**Supplementary Table S19. Annotated (non-hypothetical) genes within each genomic island**

| **GI** | **Locus tag** | **Gene / label** | **Product annotation** |
| --- | --- | --- | --- |
| GI_1 | HEDAFHDN_00447 | xerC | Tyrosine recombinase XerC |
| GI_1 | HEDAFHDN_00452 | — | Ethylmalonyl-CoA/methylmalonyl-CoA epimerase |
| GI_1 | HEDAFHDN_00453 | — | 30S ribosome-binding factor |
| GI_2 | HEDAFHDN_01653 | nusG | Transcription termination/antitermination protein NusG |
| GI_2 | HEDAFHDN_01654 | rplK | 50S ribosomal protein L11 |
| GI_2 | HEDAFHDN_01655 | rplA | 50S ribosomal protein L1 |
| GI_2 | HEDAFHDN_01656 | rplJ | 50S ribosomal protein L10 |
| GI_2 | HEDAFHDN_01657 | rplL | 50S ribosomal protein L7/L12 |
| GI_2 | HEDAFHDN_01658 | — | IS110 family transposase |
| GI_2 | HEDAFHDN_01659 | rpoB | DNA-directed RNA polymerase subunit beta |
| GI_2 | HEDAFHDN_01660 | rpoC | DNA-directed RNA polymerase subunit beta′ |
| GI_3 | HEDAFHDN_03549 | — | 2-hydroxy-3-oxopropionate reductase |
| GI_3 | HEDAFHDN_03554 | — | 2-haloacrylate reductase |
| GI_3 | HEDAFHDN_03555 | hxlR | HTH-type transcriptional activator HxlR |
| GI_3 | HEDAFHDN_03564 | lytR | Sensory transduction protein LytR |
| GI_3 | HEDAFHDN_03566 | bepF | Efflux pump periplasmic linker BepF |
| GI_3 | HEDAFHDN_03567 | bepE | Efflux pump membrane transporter BepE |
| GI_3 | HEDAFHDN_03568 | oprM | Outer membrane protein OprM |
| GI_3 | HEDAFHDN_03569 | yghO | Protein YghO |
| GI_3 | HEDAFHDN_03570 | entS | Enterobactin exporter EntS |
| GI_3 | HEDAFHDN_03572 | ddl | D-alanine–D-alanine ligase |
| GI_3 | HEDAFHDN_03576 | — | IS3 family transposase ISLbl1 |

**Supplementary Table S20. Unique90 genes that fall inside GI_2 and GI_3 (with UGI membership)**

| **GI** | **Unique90 locus tag** | **Product (Unique90 master annotation)** | **UGI** |
| --- | --- | --- | --- |
| GI_2 | HEDAFHDN_01658 | IS110 family transposase ISCaa14 | edge_1_UGI08_1697243–1698232 |
| GI_3 | HEDAFHDN_03549 | 2-hydroxy-3-oxopropionate reductase | edge_1_UGI29_3814746–3820038 |
| GI_3 | HEDAFHDN_03554 | 2-haloacrylate reductase | edge_1_UGI29_3814746–3820038 |
| GI_3 | HEDAFHDN_03555 | HTH-type transcriptional activator HxlR | edge_1_UGI29_3814746–3820038 |
| GI_3 | HEDAFHDN_03576 | IS3 family transposase ISLbl1 | edge_1_UGI30_3846197–3847075 |

**Supplementary Table S21. COG functional-category composition within predicted genomic islands (GI_1-GI_3)**

| **COG category** | **GI_1** | **GI_2** | **GI_3** | **Total (count)** |
| --- | --- | --- | --- | --- |
| **C** | 0 (0.0%) | 1 (7.7%) | 1 (2.3%) | 2 |
| **D** | 1 (10.0%) | 0 (0.0%) | 0 (0.0%) | 1 |
| **E** | 1 (10.0%) | 0 (0.0%) | 1 (2.3%) | 2 |
| **F** | 0 (0.0%) | 0 (0.0%) | 1 (2.3%) | 1 |
| **G** | 0 (0.0%) | 0 (0.0%) | 2 (4.5%) | 2 |
| **I** | 0 (0.0%) | 0 (0.0%) | 1 (2.3%) | 1 |
| **J** | 1 (10.0%) | 4 (30.8%) | 0 (0.0%) | 5 |
| **K** | 2 (20.0%) | 4 (30.8%) | 9 (20.5%) | 15 |
| **L** | 4 (40.0%) | 1 (7.7%) | 4 (9.1%) | 9 |
| **M** | 0 (0.0%) | 1 (7.7%) | 11 (25.0%) | 12 |
| **O** | 0 (0.0%) | 0 (0.0%) | 2 (4.5%) | 2 |
| **P** | 0 (0.0%) | 0 (0.0%) | 2 (4.5%) | 2 |
| **S** | 1 (10.0%) | 0 (0.0%) | 6 (13.6%) | 7 |
| **T** | 0 (0.0%) | 1 (7.7%) | 2 (4.5%) | 3 |
| **U** | 0 (0.0%) | 1 (7.7%) | 1 (2.3%) | 2 |
| **V** | 0 (0.0%) | 0 (0.0%) | 1 (2.3%) | 1 |

*Values are shown as **count (percent within each GI)**, calculated using **COG-assigned genes only** (GI_1 n=10; GI_2 n=13; GI_3 n=44). C, energy production & conversion; D, cell cycle control/division; E, amino acid transport & metabolism; F, nucleotide transport & metabolism; G, carbohydrate transport & metabolism; I, lipid transport & metabolism; J, translation/ribosomal biogenesis; K, transcription; L, replication/recombination/repair; M, cell wall/membrane/envelope biogenesis; O, posttranslational modification/protein turnover/chaperones; P, inorganic ion transport & metabolism; S, function unknown; T, signal transduction; U, intracellular trafficking/secretion/vesicular transport; V, defense mechanisms.

**Supplementary Table S22.Dominant COG categories per GI (COG-assigned genes only)**

| **Genomic island** | **COG-assigned genes (n)** | | **Top categories (count, %)** |
| --- | --- | --- | --- |
| GI_1 | 10 | L = 4 (40.0%); K = 2 (20.0%); D/E/J/S = 1 (10.0% each) | |
| GI_2 | 13 | J = 4 (30.8%); K = 4 (30.8%); C/L/M/T/U = 1 (7.7% each) | |
| GI_3 | 44 | M = 11 (25.0%); K = 9 (20.5%); S = 6 (13.6%); L = 4 (9.1%) | |

**Supplementary Table S23.Enrichment of COG functional categories in genomic islands relative to genomic background**

| **Genomic island** | **COG category** | **GI (n/N)** | **BG (n/N)** | **GI prop** | **BG prop** | **Fold-change** | **Fisher (right) p** | **BH-FDR** |
| --- | --- | --- | --- | --- | --- | --- | --- | --- |
| GI_1 | L | 4/10 | 170/2895 | 0.4 | 0.0587 | 6.81 | 0.00196 | 0.0373 |
| GI_1 | D | 1/10 | 25/2895 | 0.1 | 0.00864 | 11.58 | 0.0861 | 0.818 |
| GI_1 | K | 2/10 | 195/2895 | 0.2 | 0.0674 | 2.97 | 0.144 | 0.911 |
| GI_1 | J | 1/10 | 151/2895 | 0.1 | 0.0522 | 1.92 | 0.416 | 1 |
| GI_1 | E | 1/10 | 175/2895 | 0.1 | 0.0604 | 1.65 | 0.465 | 1 |
| GI_1 | S | 1/10 | 879/2895 | 0.1 | 0.304 | 0.329 | 0.973 | 1 |
| GI_2 | J | 4/13 | 148/2892 | 0.308 | 0.0512 | 6.01 | 0.00356 | 0.0675 |
| GI_2 | K | 4/13 | 193/2892 | 0.308 | 0.0667 | 4.61 | 0.00902 | 0.0857 |
| GI_2 | U | 1/13 | 29/2892 | 0.0769 | 0.01 | 7.67 | 0.126 | 0.801 |
| GI_2 | T | 1/13 | 101/2892 | 0.0769 | 0.0349 | 2.2 | 0.372 | 1 |
| GI_2 | C | 1/13 | 123/2892 | 0.0769 | 0.0425 | 1.81 | 0.434 | 1 |
| GI_2 | L | 1/13 | 173/2892 | 0.0769 | 0.0598 | 1.29 | 0.553 | 1 |
| GI_2 | M | 1/13 | 277/2892 | 0.0769 | 0.0958 | 0.803 | 0.73 | 1 |
| GI_3 | M | 11/44 | 267/2861 | 0.25 | 0.0933 | 2.68 | 0.00218 | 0.0212 |
| GI_3 | K | 9/44 | 188/2861 | 0.205 | 0.0657 | 3.11 | 0.00223 | 0.0212 |
| GI_3 | L | 4/44 | 170/2861 | 0.0909 | 0.0594 | 1.53 | 0.268 | 1 |
| GI_3 | U | 1/44 | 29/2861 | 0.0227 | 0.0101 | 2.24 | 0.369 | 1 |
| GI_3 | T | 2/44 | 100/2861 | 0.0455 | 0.035 | 1.3 | 0.462 | 1 |
| GI_3 | O | 2/44 | 113/2861 | 0.0455 | 0.0395 | 1.15 | 0.526 | 1 |
| GI_3 | F | 1/44 | 56/2861 | 0.0227 | 0.0196 | 1.16 | 0.585 | 1 |
| GI_3 | G | 2/44 | 134/2861 | 0.0455 | 0.0468 | 0.97 | 0.619 | 1 |
| GI_3 | P | 2/44 | 145/2861 | 0.0455 | 0.0507 | 0.897 | 0.662 | 1 |
| GI_3 | V | 1/44 | 77/2861 | 0.0227 | 0.0269 | 0.844 | 0.701 | 1 |
| GI_3 | I | 1/44 | 97/2861 | 0.0227 | 0.0339 | 0.67 | 0.782 | 1 |
| GI_3 | C | 1/44 | 123/2861 | 0.0227 | 0.043 | 0.529 | 0.855 | 1 |
| GI_3 | E | 1/44 | 175/2861 | 0.0227 | 0.0612 | 0.372 | 0.937 | 1 |
| GI_3 | S | 6/44 | 874/2861 | 0.136 | 0.305 | 0.446 | 0.997 | 1 |

GI enrichment was tested per island using **right-tailed Fisher’s exact test** (over-representation). P values were corrected within each GI using **Benjamini–Hochberg FDR**. Fold-change = (GI proportion / background proportion). Only categories present in each GI are listed (others were 0 in GI and are not shown). **GI_1** showed significant enrichment for **COG L (replication/recombination/repair)** (fold-change 6.81; Fisher p=0.00196; **BH-FDR=0.0373**). **GI_3** was significantly enriched for **COG M (cell wall/membrane/envelope biogenesis)** and **COG K (transcription)** (fold-change 2.68 and 3.11; Fisher p≈0.0022; **BH-FDR=0.0212** for both). **GI_2** displayed elevated **J** and **K** proportions (fold-change 6.01 and 4.61) but did **not** reach FDR significance (BH-FDR 0.0675–0.0857).

**Supplementary Table S24. Comparative conservation of GI_2 across reference genomes (merged coverage per reference contig)**

| **GI** | **Reference sequence** | **Covered bp** | **Coverage (%)** | **Max identity (%)** | **Mean identity (len-weighted, %)** | **HSPs** |
| --- | --- | --- | --- | --- | --- | --- |
| GI_2 | gb\|JABBGF010000003.1\| | 14,655 | 92.239 | 97.623 | 96.332 | 2 |
| GI_2 | tpg\|DBES01000005.1\| | 14,080 | 88.62 | 91.16 | 88.361 | 5 |
| GI_2 | gb\|LUVZ01000044.1\| | 13,951 | 87.808 | 91.53 | 88.764 | 5 |
| GI_2 | gb\|RJTX01000001.1\| | 13,951 | 87.808 | 90.222 | 87.536 | 4 |
| GI_2 | gb\|CP033926.1\| | 13,892 | 87.437 | 90.826 | 88.207 | 4 |
| GI_2 | gb\|MUGP01000010.1\| | 13,779 | 86.726 | 89.642 | 87.923 | 5 |
| GI_2 | gb\|PPEH01000004.1\| | 13,660 | 85.977 | 91.716 | 89.135 | 5 |
| GI_2 | gb\|LAZY01000170.1\| | 12,938 | 81.433 | 91.306 | 88.537 | 4 |
| GI_2 | gb\|MAYG01000001.1\| | 12,051 | 75.85 | 91.736 | 90.937 | 4 |
| GI_2 | gb\|LLYZ01000003.1\| | 11,920 | 75.025 | 90.944 | 88.844 | 4 |
| GI_2 | gb\|CP034171.1\| | 10,633 | 66.925 | 83.687 | 83.22 | 3 |
| GI_2 | gb\|JACHFI010000012.1\| | 8,637 | 54.362 | 82.575 | 81.723 | 2 |
| GI_2 | gb\|JAYSEF010000024.1\| | 8,525 | 53.657 | 94.472 | 90.077 | 3 |
| GI_2 | gb\|JACHFI010000005.1\| | 2,165 | 13.627 | 90.076 | 84.28 | 2 |
| GI_2 | gb\|LAZY01000072.1\| | 772 | 4.859 | 91.062 | 91.062 | 1 |
| GI_2 | gb\|JABBGF010000001.1\| | 112 | 0.705 | 91.071 | 91.071 | 1 |

* Derived from BLAST HSP merging on query coordinates; qlen = 15,888 bp

**Supplementary Table S25. Comparative conservation of GI_3 across reference genomes (merged coverage per reference contig)**

| **GI** | **Reference sequence** | **Covered bp** | **Coverage (%)** | **Max identity (%)** | **Mean identity (len-weighted, %)** | **HSPs** |
| --- | --- | --- | --- | --- | --- | --- |
| GI_3 | gb\|JABBGF010000001.1\| | 29,398 | 43.373 | 96.639 | 90.658 | 25 |
| GI_3 | gb\|LUVZ01000034.1\| | 8,631 | 12.734 | 87.539 | 87.539 | 1 |
| GI_3 | gb\|LLYZ01000005.1\| | 1,859 | 2.743 | 79.925 | 79.925 | 1 |
| GI_3 | gb\|JABBGF010000003.1\| | 1,851 | 2.731 | 92.361 | 89.092 | 5 |
| GI_3 | gb\|JAYSEF010000001.1\| | 1,559 | 2.3 | 84.727 | 77.833 | 2 |
| GI_3 | gb\|LLYZ01000009.1\| | 1,361 | 2.008 | 72.785 | 72.785 | 1 |
| GI_3 | gb\|JABBGF010000007.1\| | 1,065 | 1.571 | 86.406 | 83.706 | 2 |
| GI_3 | gb\|MAYG01000001.1\| | 960 | 1.416 | 91.358 | 84.366 | 4 |
| GI_3 | gb\|JAYSEF010000006.1\| | 883 | 1.303 | 79.867 | 79.867 | 1 |
| GI_3 | gb\|PPEH01000004.1\| | 763 | 1.126 | 86.107 | 86.107 | 1 |
| GI_3 | gb\|LAZY01000142.1\| | 733 | 1.081 | 75.541 | 75.541 | 1 |
| GI_3 | gb\|LAZY01000078.1\| | 732 | 1.08 | 89.333 | 79.645 | 2 |
| GI_3 | gb\|JACHFI010000023.1\| | 728 | 1.074 | 79.372 | 79.372 | 1 |
| GI_3 | gb\|LUVZ01000002.1\| | 714 | 1.053 | 91.176 | 81.198 | 2 |
| GI_3 | tpg\|DBES01000009.1\| | 672 | 0.991 | 91.892 | 84.077 | 2 |
| GI_3 | gb\|LAZY01000032.1\| | 645 | 0.952 | 79.753 | 79.753 | 1 |
| GI_3 | gb\|RJTX01000001.1\| | 643 | 0.949 | 80.269 | 80.269 | 1 |
| GI_3 | gb\|PPEH01000012.1\| | 594 | 0.876 | 74.415 | 74.415 | 1 |
| GI_3 | gb\|LLYZ01000021.1\| | 442 | 0.652 | 88.262 | 88.262 | 1 |
| GI_3 | gb\|JABBGF010000004.1\| | 423 | 0.624 | 83.924 | 83.924 | 1 |
| GI_3 | gb\|LUVZ01000046.1\| | 325 | 0.479 | 77.812 | 77.812 | 1 |
| GI_3 | gb\|LAZY01000019.1\| | 298 | 0.44 | 85.382 | 85.382 | 1 |
| GI_3 | gb\|CP033926.1\| | 239 | 0.353 | 90.244 | 88.664 | 3 |
| GI_3 | gb\|RJTX01000005.1\| | 225 | 0.332 | 85.333 | 85.333 | 1 |
| GI_3 | gb\|LUVZ01000043.1\| | 192 | 0.283 | 92.593 | 87.114 | 2 |
| GI_3 | tpg\|DBES01000005.1\| | 192 | 0.283 | 77.949 | 77.949 | 1 |
| GI_3 | gb\|JABBGF010000005.1\| | 162 | 0.239 | 80.247 | 80.247 | 1 |
| GI_3 | gb\|JAYSEF010000008.1\| | 161 | 0.238 | 85.802 | 85.802 | 1 |
| GI_3 | gb\|LAZY01000037.1\| | 158 | 0.233 | 86.076 | 86.076 | 1 |
| GI_3 | gb\|JACHFI010000009.1\| | 142 | 0.21 | 86.713 | 86.713 | 1 |
| GI_3 | gb\|JAYSEF010000011.1\| | 112 | 0.165 | 96.226 | 95.575 | 2 |
| GI_3 | tpg\|DBES01000010.1\| | 83 | 0.122 | 89.157 | 89.157 | 1 |
| GI_3 | tpg\|DBES01000007.1\| | 79 | 0.117 | 92.593 | 92.593 | 1 |
| GI_3 | gb\|LLYZ01000020.1\| | 79 | 0.117 | 90.123 | 90.123 | 1 |
| GI_3 | gb\|CP034171.1\| | 79 | 0.117 | 90.123 | 90.123 | 1 |
| GI_3 | gb\|LAZY01000003.1\| | 78 | 0.115 | 92.5 | 92.5 | 1 |
| GI_3 | gb\|MAYG01000023.1\| | 78 | 0.115 | 92.5 | 92.5 | 1 |
| GI_3 | gb\|RJTX01000002.1\| | 78 | 0.115 | 91.358 | 91.358 | 1 |
| GI_3 | gb\|PPEH01000003.1\| | 78 | 0.115 | 91.25 | 91.25 | 1 |
| GI_3 | gb\|MUGP01000002.1\| | 78 | 0.115 | 86.25 | 86.25 | 1 |
| GI_3 | gb\|JAYSEF010000020.1\| | 73 | 0.108 | 89.333 | 89.333 | 1 |
| GI_3 | gb\|LUVZ01000047.1\| | 68 | 0.1 | 92.647 | 92.647 | 1 |
| GI_3 | gb\|LUVZ01000005.1\| | 68 | 0.1 | 91.176 | 91.176 | 1 |
| GI_3 | gb\|JAYSEF010000043.1\| | 55 | 0.081 | 96.364 | 96.364 | 1 |
| GI_3 | gb\|JAYSEF010000039.1\| | 53 | 0.078 | 96.226 | 96.226 | 1 |

*Derived from BLAST HSP merging on query coordinates; qlen = 67,779 bp

**Supplementary Table S26. Cross-genome conservation of NGCE01 genomic-island gene clusters (Jaccard similarity)**

| **GI** | **Main overlap pattern across references** |
| --- | --- |
| GI_1 | Mostly absent/rare: top overlap 6/26 (J=0.231), many genomes only 2/26 (J=0.077), and 3 genomes = 0/26. |
| GI_2 | Broadly conserved: one genome 12/13 (J=0.923), several 11/13 (J=0.846) and 9/13 (J=0.692); only 2 genomes = 0/13. |
| GI_3 | Largely unique: only one genome shows any overlap (12/57, J=0.211); 12 genomes = 0/57. |

*Jaccard was computed against NGCE01 GI cluster sets (Union = NGCE01 clusters because other genomes contain subsets of the NGCE01 GI cluster repertoire in this table). Values below summarize 13 reference genomes per GI (excluding NGCE01 self-comparison).

**Supplementary Table S27. Reference selection logic for clinker synteny panels (GI_1–GI_3)**

| **GI** | **Synteny_panel generated** | **References used** | **Selection basis** | **Best_reference** | **Best_overlapstat** | **Rationale** |
| --- | --- | --- | --- | --- | --- | --- |
| GI_1 | No | 0 | Jaccard screening across 13 refs | GCA_002899875.1_ASM289987v1_genomic | J = 0.231 (6/26 shared) | GI_1 showed uniformly low gene-content overlap across references (most J ≤ 0.077; several 0), indicating weak conservation and poor synteny informativeness; adding references would produce mostly unmatched hypothetical ORFs and clutter without improving biological interpretability. |
| GI_2 | Yes | 5 | Top Jaccard ranks (most shared clusters) | GCA_012927265.1_ASM1292726v1_genomic | J = 0.923 (12/13 shared) | GI_2 is broadly conserved; five highest-overlap references (J = 0.923, 0.846, 0.846, 0.846, 0.769) were chosen to visualize a shared backbone and stable gene order while keeping the panel readable. |
| GI_3 | Yes | 1 | Nonzero overlap only | GCA_012927265.1_ASM1292726v1_genomic | J = 0.211 (12/57 shared) | GI_3 is highly isolate-specific: only one reference had any detectable gene-content overlap (J = 0.211), while all other references were J = 0.0. Including additional genomes would add empty/unaligned tracks and reduce clarity; a single best-hit reference provides the most interpretable “conserved fragment within mosaic island” view. |

**Supplementary Table S28. ISfinder web screening hits retained as high-confidence**

| **ISfinder query** | **IS family** | **Group** | **Reported origin (ISfinder)** | **Bit score** | **E-value** |
| --- | --- | --- | --- | --- | --- |
| ISSau3 | **IS1182** | — | *Staphylococcus aureus* | 77.8 | 1e−10 |
| ISSmi2 | **IS1182** | — | *Streptococcus mitis* | 67.9 | 1e−07 |
| ISLpn9 | **IS4** | IS10 | *Legionella pneumophila* | 56 | 5e−04 |

*Retention criterion: E-value ≤ 1×10⁻³ from the ISfinder web output

**Supplementary Table S29. Local validation of high-confidence ISfinder signals in NG-CE01**

| **ISfinder query** | **Local mapping outcome on edge_1** | **Locus context** | **Pfam/domain validation** | **Final interpretation** |
| --- | --- | --- | --- | --- |
| ISSau3 (IS1182) | Short, significant matches at multiple loci; co-localizes with ISSmi2 in ~1.14 Mb region | Motifs overlap ORF ID=1_4 (473 aa) in inspected window | DUF772 (PF05598; 1.4×10⁻²⁰) + DDE_Tnp_1 (PF01609; 1.3×10⁻¹⁷) (+ DDE_Tnp_1_6; 1.2×10⁻¹⁵) | Confirmed DDE transposase locus (likely intact/functional CDS); full IS boundaries not resolved (no TIR detected) |
| ISSmi2 (IS1182) | Short, significant matches at multiple loci; co-localizes with ISSau3 in ~1.14 Mb region | Same ORF ID=1_4 | Same as above | Confirmed DDE transposase locus (likely intact/functional CDS); full IS boundaries not resolved (no TIR detected) |
| ISLpn9 (IS4/IS10) | Multiple short (≈28–32 bp) matches within ~3.62 Mb neighborhood | Matches fall intergenic between ORF 1_4 and ORF 1_5 (no CDS overlap) | Flanking ORFs show non-transposase domains (FGGY kinase; other enzyme/domain hit); no DDE/transposase signatures | Motif-level similarity only; not supported as an IS element at this locus |

**Supplementary Table S30. Screening table (Panaroo unique genes flagged as IS/transposase)**

| **UGI block** | **Contig** | **Start** | **End** | **Strand** | **Locus_tag** | **Annotation (Panaroo)** | **COG** |
| --- | --- | --- | --- | --- | --- | --- | --- |
| UGI01_217481-218119 | edge_1 | 217481 | 218119 | − | HEDAFHDN_00218 | IS110 family transposase ISCaa14 | L |
| UGI08_1697243-1698232 | edge_1 | 1697243 | 1698232 | + | HEDAFHDN_01658 | IS110 family transposase ISCaa14 | L |
| UGI09_1825291-1825995 | edge_1 | 1825291 | 1825995 | + | HEDAFHDN_01777 | IS1182 family transposase ISMsp1 | L |
| UGI12_2388364-2389341 | edge_1 | 2388364 | 2389341 | + | HEDAFHDN_02294 | IS110 family transposase ISCaa7 | L |
| UGI15_2667302-2668291 | edge_1 | 2667302 | 2668291 | + | HEDAFHDN_02532 | IS110 family transposase ISCaa14 | L |
| UGI30_3846197-3847075 | edge_1 | 3846197 | 3847075 | + | HEDAFHDN_03576 | IS3 family transposase ISLbl1 | L |
| UGI31_3928957-3929946 | edge_1 | 3928957 | 3929946 | − | HEDAFHDN_03648 | IS110 family transposase ISCaa14 | L |
| UGI34_4126182-4127159 | edge_1 | 4126182 | 4127159 | − | HEDAFHDN_03832 | IS110 family transposase ISCaa7 | L |
| UGI35_4187963-4188940 | edge_1 | 4187963 | 4188940 | + | HEDAFHDN_03882 | IS110 family transposase ISCaa7 | L |
| UGI35_4192961-4193938 | edge_1 | 4192961 | 4193938 | + | HEDAFHDN_03886 | IS110 family transposase ISCaa7 | L |

*These are the 10 unique genes from the Panaroo presence/absence matrix that are IS/transposase-related.

**Supplementary Table S31. Validation table (Pfam domain evidence and locus status)**

| **Locus_tag** | **AA length** | **Pfam domains (E ≤ 1e−5)** | **Validation call** |
| --- | --- | --- | --- |
| HEDAFHDN_00218 | 212 | Transposase_20 (PF02371; 2.6e−19) | Likely truncated/fragmented transposase |
| HEDAFHDN_01658 | 329 | Transposase_20 (PF02371; 1.1e−19); DEDD_Tnp_IS110 (PF01548; 1.7e−14) | Intact IS110 transposase CDS (high confidence) |
| HEDAFHDN_01777 | 234 | DUF772 (PF05598; 3.7e−21) | Likely truncated/fragmented transposase |
| HEDAFHDN_02294 | 325 | DEDD_Tnp_IS110 (PF01548; 3.1e−18); Transposase_20 (PF02371; 1.1e−17) | Intact IS110 transposase CDS (high confidence) |
| HEDAFHDN_02532 | 329 | Transposase_20 (PF02371; 1.1e−19); DEDD_Tnp_IS110 (PF01548; 1.7e−14) | Intact IS110 transposase CDS (high confidence) |
| HEDAFHDN_03576 | 292 | rve (PF00665; 3.5e−21); rve_3 (PF13683; 1.1e−13); rve_2 (PF13333; 1.9e−12); HTH_21 (PF13276; 1.8e−11) | Integrase-like mobile gene; not a canonical IS transposase |
| HEDAFHDN_03648 | 329 | Transposase_20 (PF02371; 1.1e−19); DEDD_Tnp_IS110 (PF01548; 1.7e−14) | Intact IS110 transposase CDS (high confidence) |
| HEDAFHDN_03832 | 325 | DEDD_Tnp_IS110 (PF01548; 3.1e−18); Transposase_20 (PF02371; 1.1e−17) | Intact IS110 transposase CDS (high confidence) |
| HEDAFHDN_03882 | 325 | DEDD_Tnp_IS110 (PF01548; 3.1e−18); Transposase_20 (PF02371; 1.1e−17) | Intact IS110 transposase CDS (high confidence) |
| HEDAFHDN_03886 | 325 | DEDD_Tnp_IS110 (PF01548; 3.1e−18); Transposase_20 (PF02371; 1.1e−17) | Intact IS110 transposase CDS (high confidence) |

**Supplementary Table S32. Detailed validation summary (two representative IS110 loci)**

| **Locus** | **Genome coords (edge_1)** | **Window used** | **Transposase ORF (window coords)** | **Pfam evidence (E≤1e−5)** | **IR-like ends** | **TSD (4–10 bp)** | **Final call** |
| --- | --- | --- | --- | --- | --- | --- | --- |
| IS110A (HEDAFHDN_01658) | 1,697,243–1,698,232 (+) | 1,695,243–1,700,232 (4,990 bp) | 2001–2990 (+) | PF02371 (1.1e−19); PF01548 (1.7e−14) | 14 bp, 1 mismatch | Not detected | Intact IS110 transposase; termini unresolved (likely degraded/divergent) |
| IS110B (HEDAFHDN_02294) | 2,388,364–2,389,341 (+) | 2,386,364–2,391,341 (4,978 bp) | 2001–2978 (+) | PF01548 (3.1e−18); PF02371 (1.1e−17) | 14 bp, 2 mismatches | Not detected | Intact IS110 transposase; termini unresolved (likely degraded/divergent) |

**Supplementary Table S33. Prophage-like region overview**

| **Contig** | **Boundaries  (attL–attR)** | **Span (bp)** | **Span (kb)** | **PHASTER call** | **PHASTER score** | **CDS (n)** | **Key hits** | **Cross-tool agreement** |
| --- | --- | --- | --- | --- | --- | --- | --- | --- |
| edge_1 | 2406137..2428202 | 22,066 | 22.07 | Incomplete | 20 | 14 | methyltransferase; tail assembly; HTH-like regulator | PHASTEST: no prophage call |

**Supplementary Table S34. Gene-by-gene annotation of the PHASTER “incomplete prophage-like” region**

| **Feature/ORF #** | **Coordinates (edge_1)** | | **Strand** | **Best hit / annotation (PHASTER)** | **Locus tag** | **E-value** |
| --- | --- | --- | --- | --- | --- | --- |
| **attL** | | **2406137..2406149** | **+** | **attL** | **—** | **0** |
| 2 | | 2406320..2407459 | + | Methyltransferase (type II); PHAGE_Flavob_vB_FspS_laban6_1 (NC_048834) | HEDAFHDN_02311 | 2.40E-12 |
| 3 | | 2407652..2407936 | + | Hypothetical protein; PHAGE_Brevib_Abouo (NC_029029) | HEDAFHDN_02312 | 4.04E-06 |
| 4 | | 2408040..2409200 | + | Hypothetical protein; PHAGE_Flavob_vB_FspM_pippi8_1 (NC_048830) | HEDAFHDN_02313 | 3.03E-48 |
| 5 | | 2409442..2410323 | + | Hypothetical protein | HEDAFHDN_02314 | 0 |
| 6 | | 2410320..2410958 | + | Hypothetical protein | HEDAFHDN_02315 | 0 |
| 7 | | 2411364..2411705 | + | Hypothetical protein | HEDAFHDN_02316 | 0 |
| 8 | | 2411692..2411922 | + | Hypothetical protein | HEDAFHDN_02317 | 0 |
| 9 | | 2412082..2413515 | + | Phage protein (ea59); PHAGE_Entero_lambda (NC_001416) | HEDAFHDN_02318 | 2.59E-05 |
| 10 | | complement(2413711..2414790) | − | Tail assembly protein; PHAGE_Bacill_Mgbh1 (NC_041879) | HEDAFHDN_02319 | 4.78E-15 |
| 11 | | complement(2414807..2415622) | − | Hypothetical protein | HEDAFHDN_02320 | 0 |
| 12 | | 2416017..2416241 | + | Helix–turn–helix (HTH) domain protein; PHAGE_Bacill_IEBH (NC_011167) | HEDAFHDN_02321 | 2.27E-05 |
| 13 | | 2416384..2417982 | + | Hypothetical protein; PHAGE_Mycoba_Panchino (NC_031281) | HEDAFHDN_02322 | 1.07E-07 |
| 14 | | 2417979..2419835 | + | Hypothetical protein; PHAGE_Lactob_BH1 (NC_048737) | HEDAFHDN_02323 | 1.02E-17 |
| 15 | | 2419860..2420687 | + | Hypothetical protein; PHAGE_Salmon_SEN8 (NC_047753) | HEDAFHDN_02324 | 6.17E-14 |
| **attR** | | **2428190..2428202** | **+** | **attR** | **—** | **0** |

**Supplementary Table S35. ICEfinder overview of a conjugation-/mobility-associated locus in NG-CE01.**

| **Element** | **Contig** | **Coordinates** | **Length (bp)** | **ICEfinder call** | **Key hallmarks** |
| --- | --- | --- | --- | --- | --- |
| Region1 | edge_1 | 3,720,179–3,736,023 | 15,845 | T4SS-associated mobility locus (ICEfinder call) | T4CP, Tra proteins, VirB4, relaxase |

**Supplementary Table S36. Feature-level summary of the T4SS-associated mobility locus in NG-CE01 (ICEfinder Region1).**

| **Feature** | **Description** |
| --- | --- |
| **Locus ID (ICEfinder Region1)** | ICE-NGCE01-1 (ICEfinder output label) |
| **Element type** | **T4SS-associated conjugation/mobility locus (ICEfinder call)** |
| **Genomic location (nt)** | edge_1: 3,720,179 – 3,736,023 |
| **Length (bp)** | 15,845 |
| **GC content (%)** | 42.16 |
| **Copy number** | Single |
| **Association with tRNA** | No |
| **Direct repeats (DRs)** | Not detected |
| **oriT** | Not detected |
| **Relaxase family** | MOBP1 |
| **Mating pair formation system** | Type B (MPF-B) |
| **Cargo genes** | Predominantly hypothetical proteins |
| **AMR genes** | None detected (ResFinder: 0 hits within ICE interval) |
| **Virulence genes** | None detected within the interval (no virulence keywords; VFDB not required for this claim) |

The locus is described conservatively as a T4SS-associated mobility region because classical ICE integration hallmarks (e.g., clear att boundaries and/or tRNA association) were not definitively supported in the flanks.

**Supplementary Table S37. Gene content of ICEfinder Region1 (T4SS-associated mobility locus) in NG-CE01.**

| **Gene / locus tag** | **Coordinates (edge_1)** | **Strand** | **Size (bp)** | **Best annotation** | **Feature class** |
| --- | --- | --- | --- | --- | --- |
| HEDAFHDN_03458 | 3715607..3715868 | − | 262 | hypothetical protein | Flank |
| HEDAFHDN_03459 | 3716171..3716855 | − | 685 | hypothetical protein | Flank |
| HEDAFHDN_03460 | 3716921..3718577 | − | 1657 | hypothetical protein | Flank |
| HEDAFHDN_03461 | 3718633..3719329 | − | 697 | hypothetical protein | Flank |
| HEDAFHDN_03462 | 3719364..3720180 | − | 817 | hypothetical protein | Flank |
| HEDAFHDN_03463 | 3720179..3722171 | − | 1993 | t4cp2 | T4CP |
| HEDAFHDN_03464 | 3722231..3723098 | − | 868 | B_traN | T4SS |
| HEDAFHDN_03465 | 3723102..3724305 | − | 1204 | B_traM | T4SS |
| HEDAFHDN_03466 | 3724338..3724692 | − | 355 | hypothetical protein | — |
| HEDAFHDN_03467 | 3724693..3725308 | − | 616 | B_traK | T4SS |
| HEDAFHDN_03468 | 3725375..3726623 | − | 1249 | B_traJ | T4SS |
| HEDAFHDN_03469 | 3726625..3727456 | − | 832 | hypothetical protein | — |
| HEDAFHDN_03470 | 3727461..3728127 | − | 667 | hypothetical protein | — |
| HEDAFHDN_03471 | 3728129..3728813 | − | 685 | hypothetical protein | — |
| HEDAFHDN_03472 | 3728823..3729477 | − | 655 | hypothetical protein | — |
| HEDAFHDN_03473 | 3729463..3732541 | − | 3079 | virB4  (DnaJ-chaperone annotated in neighborhood) | T4SS |
| HEDAFHDN_03474 | 3732544..3732826 | − | 283 | B_traF | T4SS |
| HEDAFHDN_03475 | 3732829..3733150 | − | 322 | B_traE | T4SS |
| HEDAFHDN_03476 | 3733262..3733847 | − | 586 | hypothetical protein | — |
| HEDAFHDN_03477 | 3733867..3734518 | − | 652 | hypothetical protein | — |
| HEDAFHDN_03478 | 3734583..3736023 | − | 1441 | MOBP1 | Relaxase |
| HEDAFHDN_03479 | 3736034..3736370 | − | 337 | hypothetical protein | Flank |
| HEDAFHDN_03480 | 3736392..3736836 | − | 445 | hypothetical protein | Flank |
| HEDAFHDN_03481 | 3736965..3737142 | + | 178 | hypothetical protein | Flank |
| HEDAFHDN_03482 | 3737740..3739222 | + | 1483 | hypothetical protein | Flank |
| HEDAFHDN_03483 | 3739226..3742121 | + | 2896 | hypothetical protein | Flank |

***attL/attR**: No canonical short, near-perfect direct repeats were recovered at Region1 boundaries using non-overlapping flank comparisons (upstream vs downstream). **Integrase/recombinase**: Not detected in the local ±50 kb neighborhood by keyword screening or PFAM-domain interrogation (integrase/recombinase families). **Cargo**: ResFinder = 0 hits within the 15,845 bp **Region1 interval**; no IS/transposase/metal/stress keyword hits inside Region1. **Context**: A separate PHASTER incomplete prophage (with attL/attR) occurs at 2.406–2.428 Mb on edge_1 and is distinct from Region1 (3.720–3.736 Mb).

**Supplementary Table S38. Partitioning of the 90 NG-CE01–unique genes into curated locus categories.**

| **Category** | **Loci included** | **Gene count** | **% of Unique90 (N=90)** |
| --- | --- | --- | --- |
| Resistance / AMR-like (intrinsic) | R1–R5 | 11 | 12.22% |
| Virulence / fitness-like | V1–V5 | 8 | 8.89% |
| Genome defense | D1–D2 | 5 | 5.56% |
| Total (curated loci categories) | — | 24 | 26.67% |
| Other Unique90 genes (not in curated loci list) | — | 66 | 73.33% |

Unique90” refers to genes classified as unique to NG-CE01 based on the Panaroo presence/absence matrix (Supplementary Table S7). Percentages are calculated as (gene count / 90) × 100.

**Supplementary Table S39. Curated resistance-like, fitness/virulence-like, and genome-defense loci in NG-CE01 with genomic coordinates, anchor genes, and annotation-based system completeness.**

| **Group** | **Locus_ID** | **Module name** | **Genomic span (contig:bp)** | **Strand** | **Core gene(s) / anchors** | **Status notes** | **System completeness (annotation scan)** | **Genome confidence** | |
| --- | --- | --- | --- | --- | --- | --- | --- | --- | --- |
| AMR_intrinsic | R1 | Efflux + regulation module | edge_1:1087501–1095274 | − | cusR_1; mdtB; mexA | One module (same region) | NA | | High |
| AMR_intrinsic | R2 | Metal/solvent efflux module | edge_1:3642842–3648499 | − | cusC; bepE_4; ttgG | One module (same region) | NA | | High |
| AMR_intrinsic | R3 | Envelope/cell-wall resistance-like locus | edge_1:3376201–3377247 | − | ampH | Single-gene locus | NA | | Medium |
| AMR_intrinsic | R4 | PBP-associated resistance-like locus | edge_1:3755158–3756459 | + | pbpE_2 | Single-gene locus | NA | | Medium |
| AMR_intrinsic | R5 | Surface/glycan & envelope remodeling locus (UGI block) | edge_1:270305–285160 | . | gnu; wecB; ptk | Confirmed: gnu + wecB + ptk within 270305–285160 | NA | | High |
| Fitness_like | V1 | SusC/SusD/TonB uptake module | edge_1:813263–824609 | − | SusD_like; TonB_receptor; susC_1 | One module (same region) | NA | | High |
| Fitness_like | V2 | Type I secretion–associated module | edge_1:1188336–1189514 | + | hlyD; lagD_3 | Confirmed adjacency: lagD_3 next to hlyD (boundary at 1189514) | Supported/Partial: hlyD + lagD_3 adjacent; TolC-family OM channels present elsewhere (OprM_1–OprM_5). No dedicated adjacent HlyB/RTX ABC exporter detected. | | High |
| Fitness_like | V3 | Iron uptake / fitness locus | edge_1:2470356–2471066 | + | magA_2 | Single-gene locus | NA | | Medium |
| Fitness_like | V4 | Outer membrane receptor locus | edge_1:3512157–3515024 | − | TonB_receptor_P3 | Single-gene locus (2nd TonB receptor) | NA | | Medium |
| Fitness_like | V5 | SusC-like outer membrane uptake locus | edge_1:3987129–3990008 | − | susC_4 | Single-gene locus | NA | | Medium |
| Defense | D1 | Toxin–antitoxin loci (ParE/ParD) | edge_1:1805333–3097659 | . | parE1_1; parE1_2; parD1 | Two sites; one paired parE1_2–parD1 region | NA | | High |
| Defense | D2 | Restriction–modification (Type I) | edge_1:2834426–3393185 | . | hsdR_1; hsdR_2 | Two sites | Partial: hsdR subunits present at two loci; no hsdM or hsdS detected by annotation-based searches | | Medium |

**System completeness** (annotation scan) indicates whether additional required partners for a multi-component system were detected elsewhere in the genome by keyword/annotation sweeps. **NA** = Not Applicable; system-completeness scoring was not applied because these modules represent curated chromosomal neighborhoods rather than standardized gene systems with defined component requirements. **Genome** confidence reflects curation confidence based on co-localization, gene context, and annotation support.


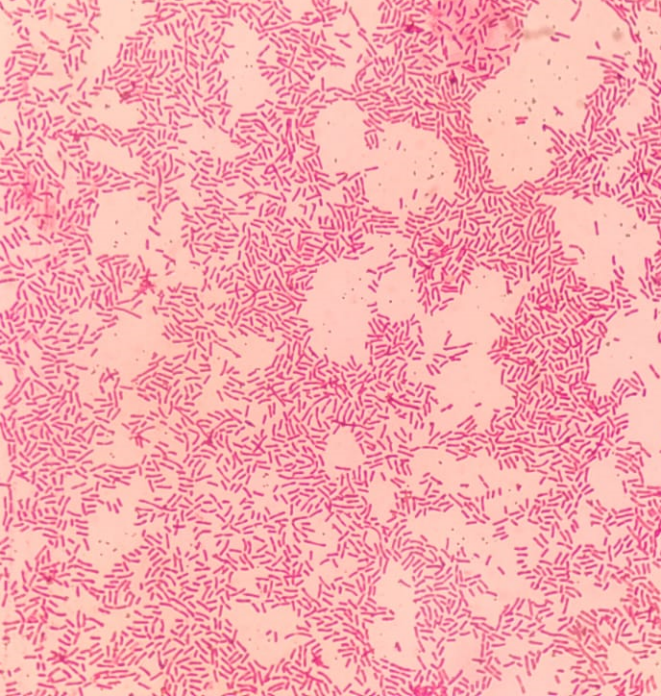


**Supplementary Figure** **S1.** **Gram-stained smear of *Chryseobacterium* sp. NG-CE01 showing pleomorphic Gram-negative rods distributed singly and in short aggregates (oil immersion).**


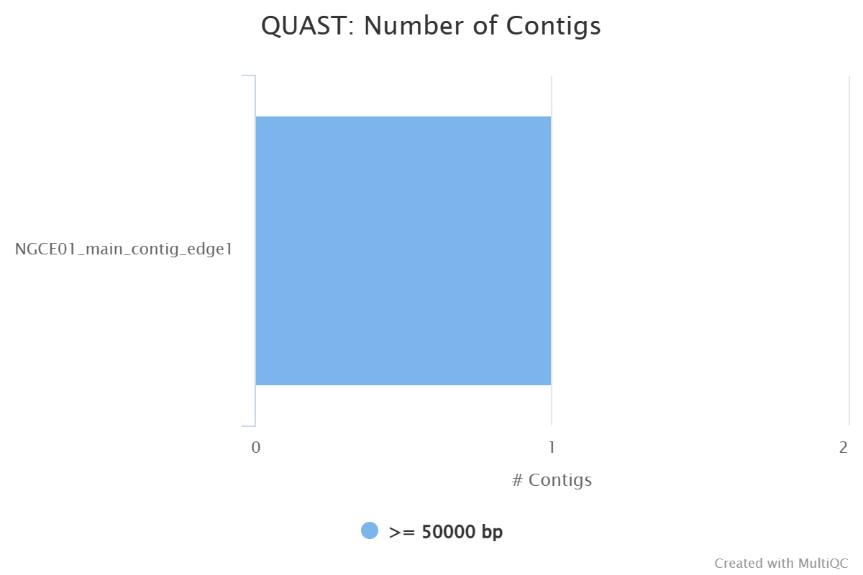


**Supplementary Figure S2. QUAST summary of assembly contiguity for *Genus species* strain.** MultiQC visualization of QUAST results showing the number of contigs (≥50,000 bp) in the final assembly. The genome is represented by a single contig, consistent with a highly contiguous assembly.


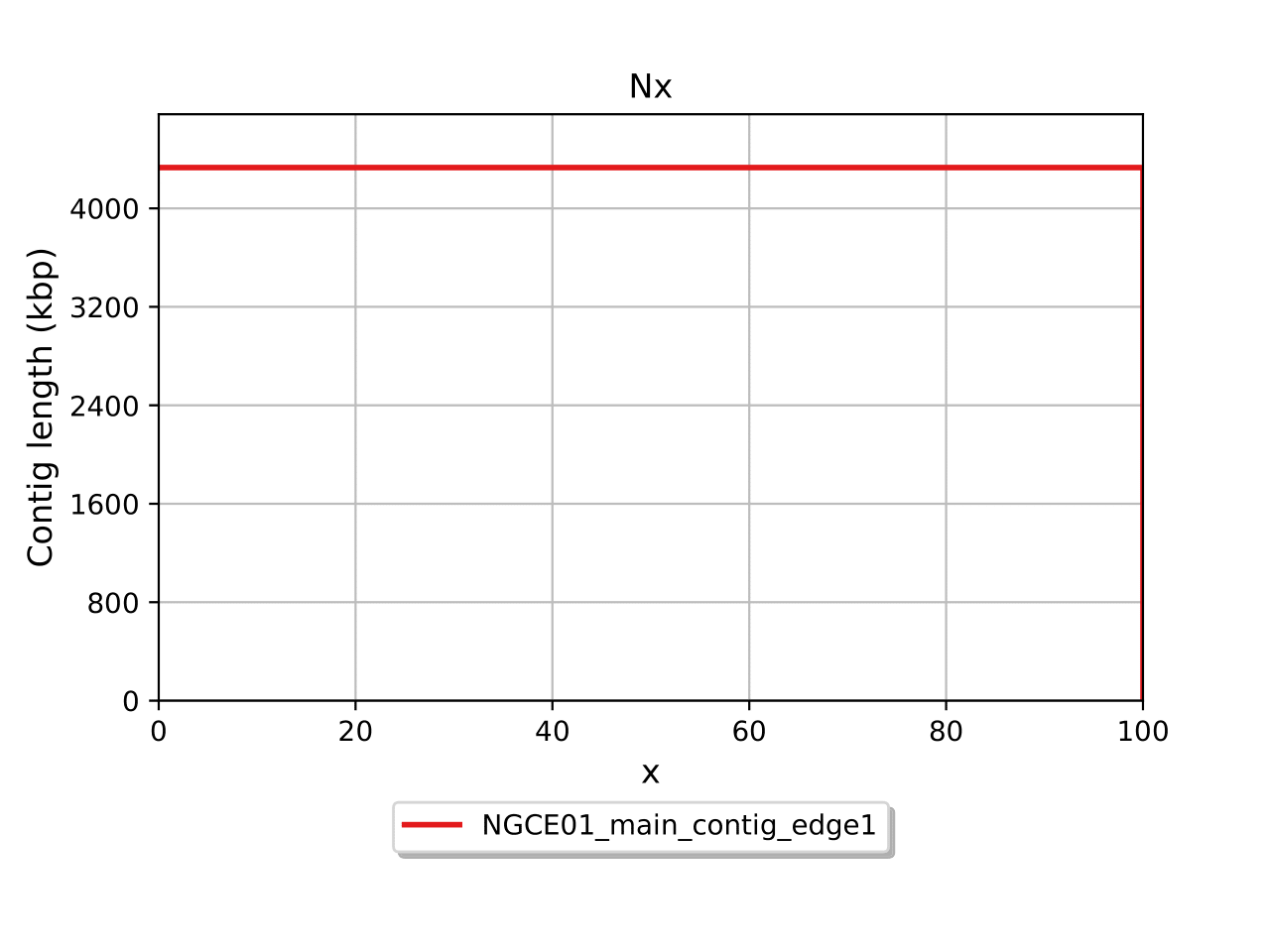


**Supplementary Figure S3. Assembly continuity profile of *Chryseobacterium* sp. NG-CE01.** Nx curve illustrating the relationship between cumulative genome coverage and contig length, supporting a coherent scaffold structure and near-complete draft genome assembly.


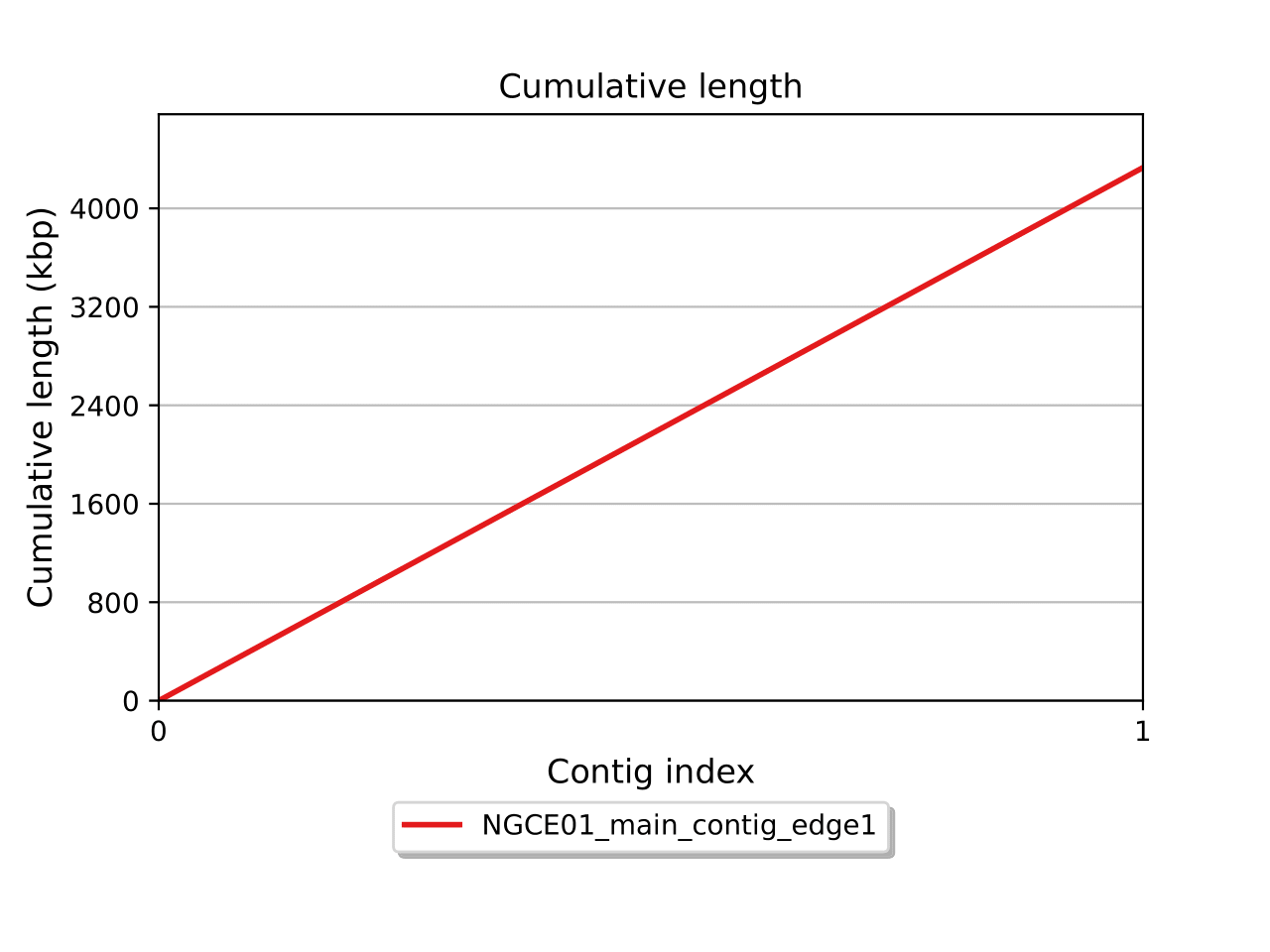


**Supplementary Fig. S4. Cumulative contig-length profile of the *Chryseobacterium* sp. NG-CE01strain assembly.** Cumulative assembly length (kbp) plotted against contig index, showing that the total genome length is reached within a single contig, consistent with a fully contiguous (single-contig) assembly.


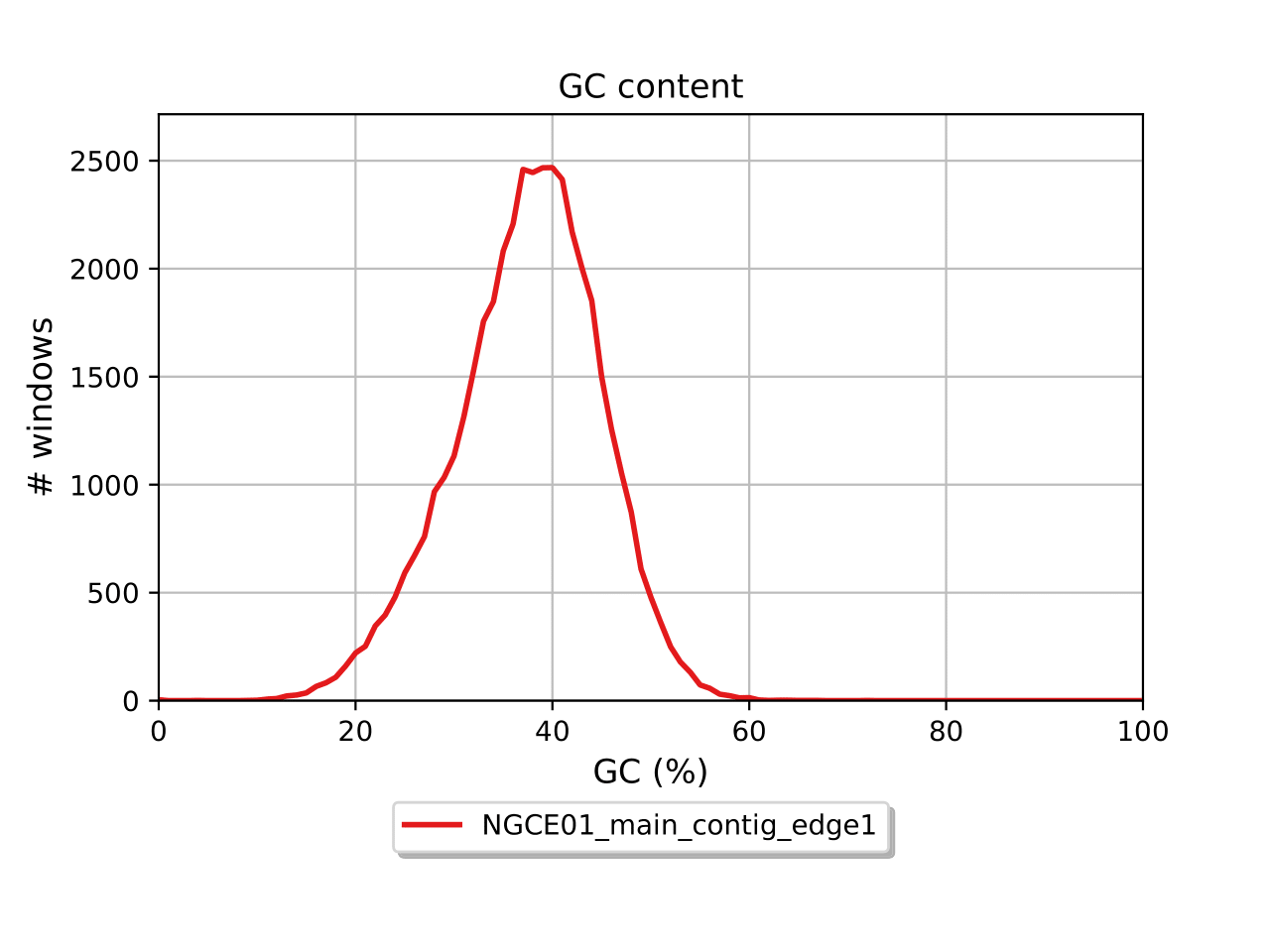


**Supplementary Figure S5. GC content distribution across assembled scaffolds.** Genome-wide GC content calculated in sliding windows across all scaffolds shows a unimodal distribution centered at 37.43 %, further supporting assembly consistency and absence of large-scale contamination.


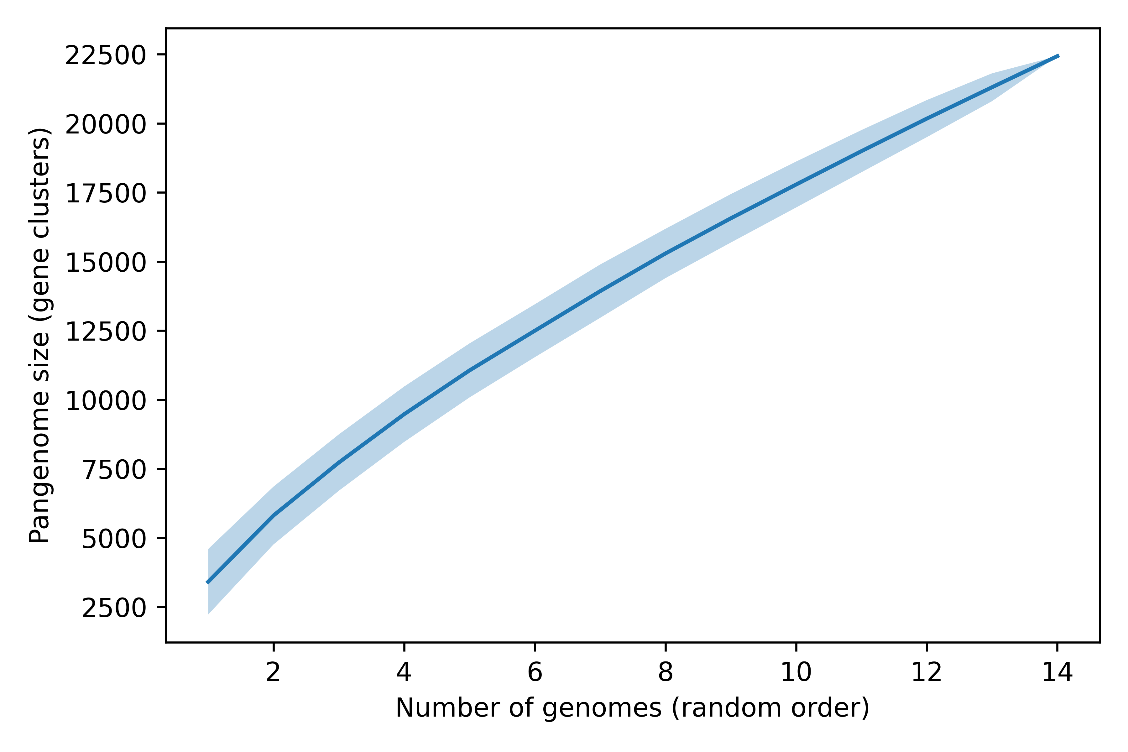


**Supplementary Figure S6.** **Gene-cluster accumulation across the curated comparator panel (multi-species), based on Panaroo clusters; openness is expected at interspecies scale..**


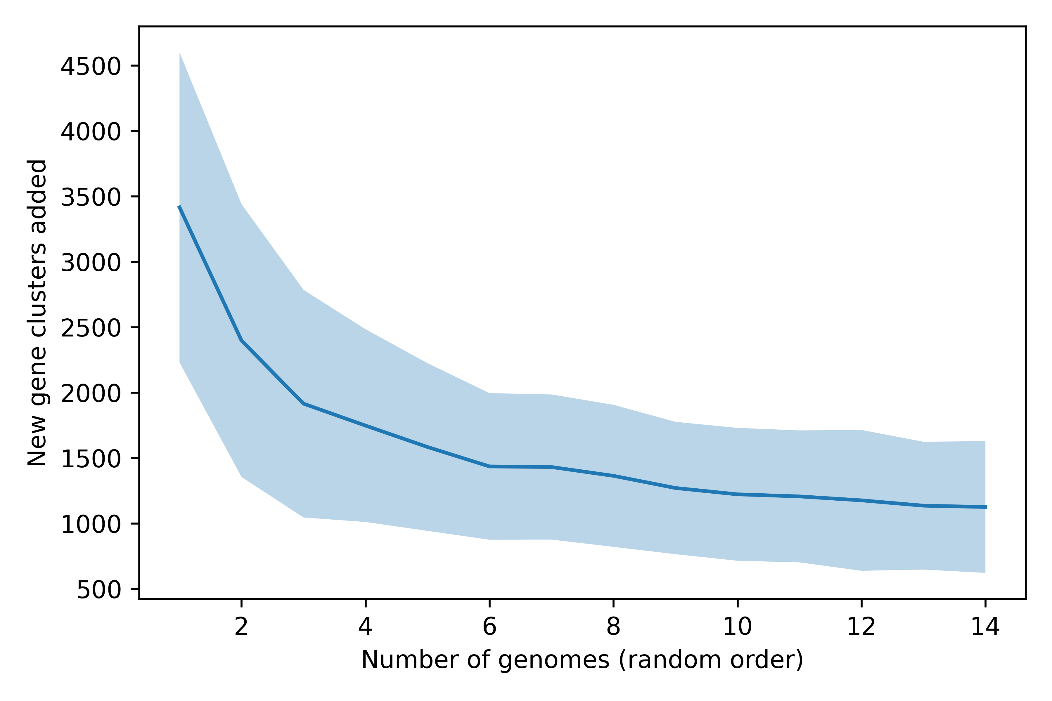


**Supplementary Figure S7. New gene-cluster discovery across the curated comparator panel (multi-species).**


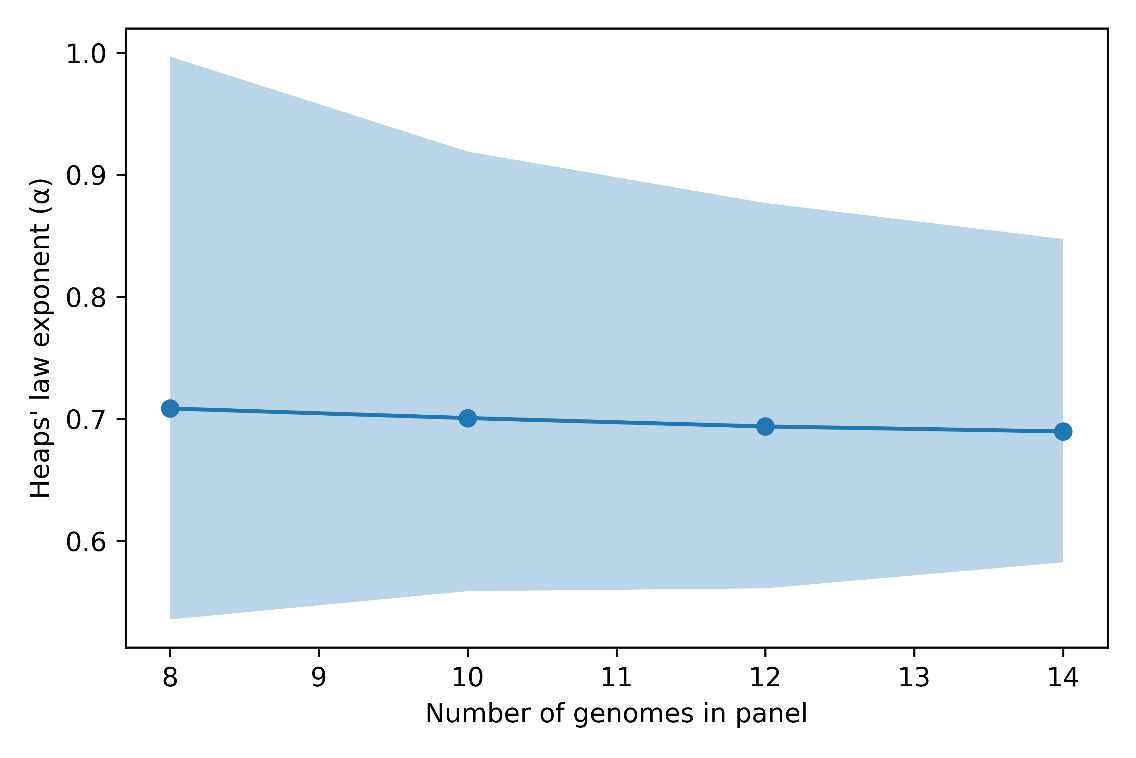


**Supplementary Figure S8.** **Sensitivity analysis of Heaps’ law exponent (α) across nested genome panels (n = 8, 10, 12, 14). Shaded region indicates 95% confidence interval of α.**


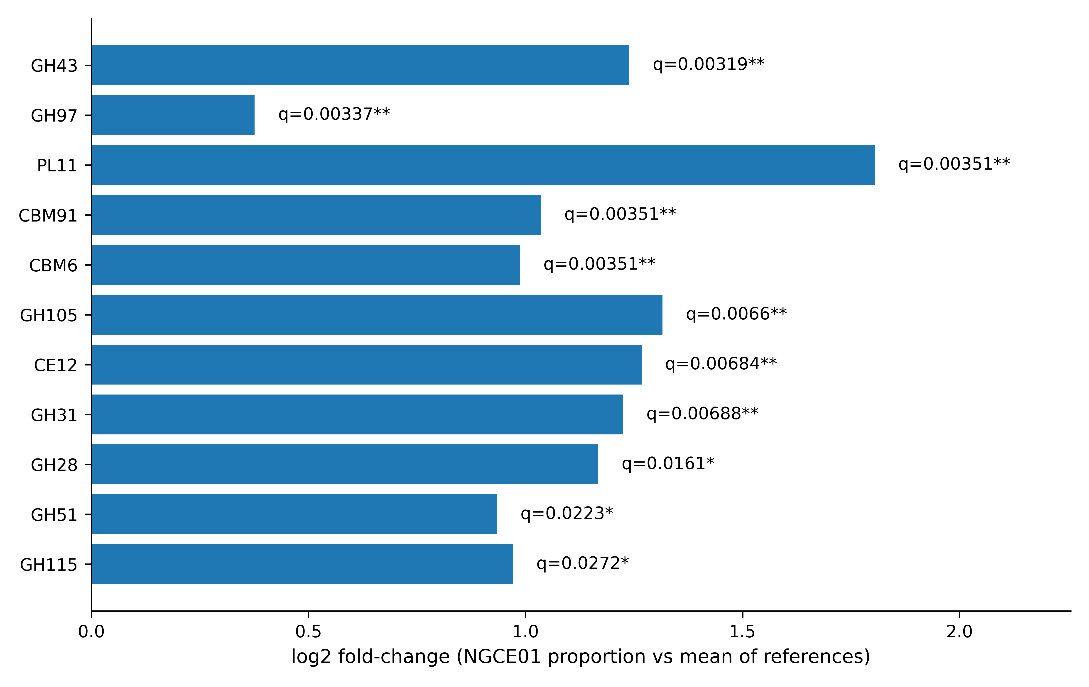


**Supplementary Figure S9. CAZyme family enrichment in NG-CE01 relative to 13 reference *Chryseobacterium* genomes.** Horizontal bars show the log2 fold-change in **family proportion** for NG-CE01 compared with the **mean proportion across the 13 reference genomes**. Only families with higher proportional representation in NG-CE01 (**log2FC > 0**) and passing multiple-testing corrections are displayed. Each label reports the **FDR-adjusted q-value** for the enrichment test, highlighting families most over-represented in NG-CE01 (e.g., GH43, PL11, GH105, GH31) consistent with expanded glycan-processing capacity. Enrichment was assessed using a **one-sided test** (NG-CE01 > references) on proportional family abundance, with p-values adjusted for multiple comparisons using the **Benjamini–Hochberg false discovery rate (FDR)** procedure. Families were considered significant at **q ≤ 0.05** (FDR cutoff). Significance symbols indicate: *q ≤ 0.05 (*), **q ≤ 0.01 (**).*


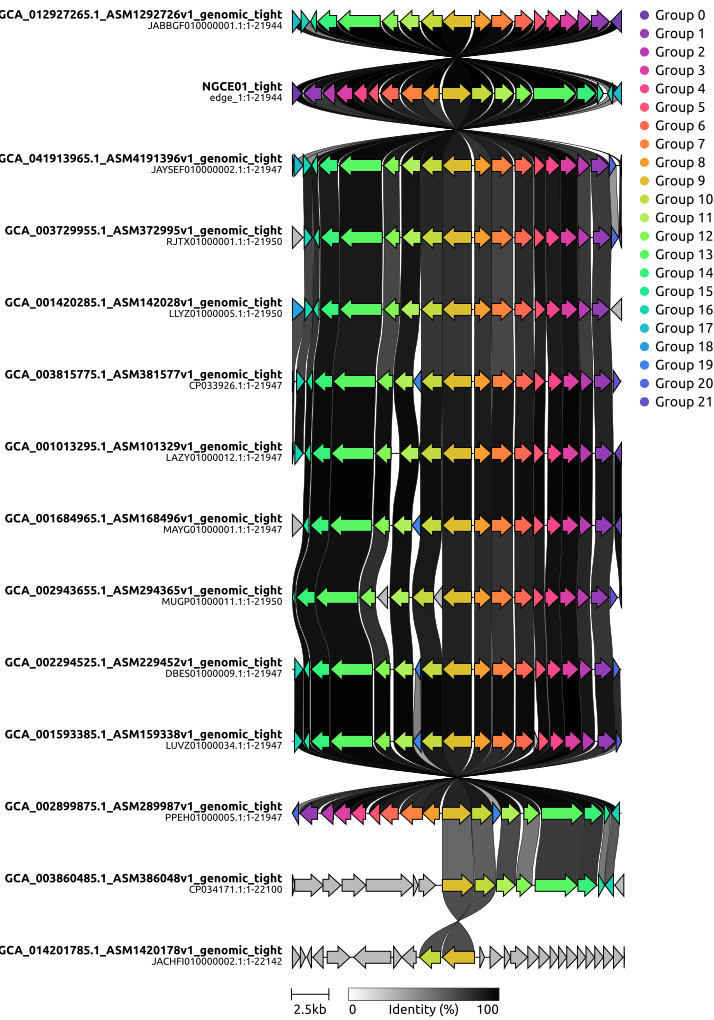


**Supplementary Figure S10. Synteny comparison of the CBM48–GH13 α-glucan utilization locus across NGCE01 and the 13-reference Chryseobacterium panel.** Clinker was used to align a tight genomic window centered on the NGCE01 CBM48–GH13 anchor and its best-hit homologs in each reference genome. Arrows represent CDS features drawn to scale and oriented by strand; genes are colored by orthology group assignments generated by clinker. Shaded links connect homologous genes between loci, with shading intensity reflecting pairwise amino-acid identity (legend). The figure shows a conserved CBM48–GH13 backbone shared across the panel, accompanied by variable accessory gene content and local rearrangements in several references, consistent with locus-level remodeling of an α-glucan foraging module rather than simple CAZyme count differences.


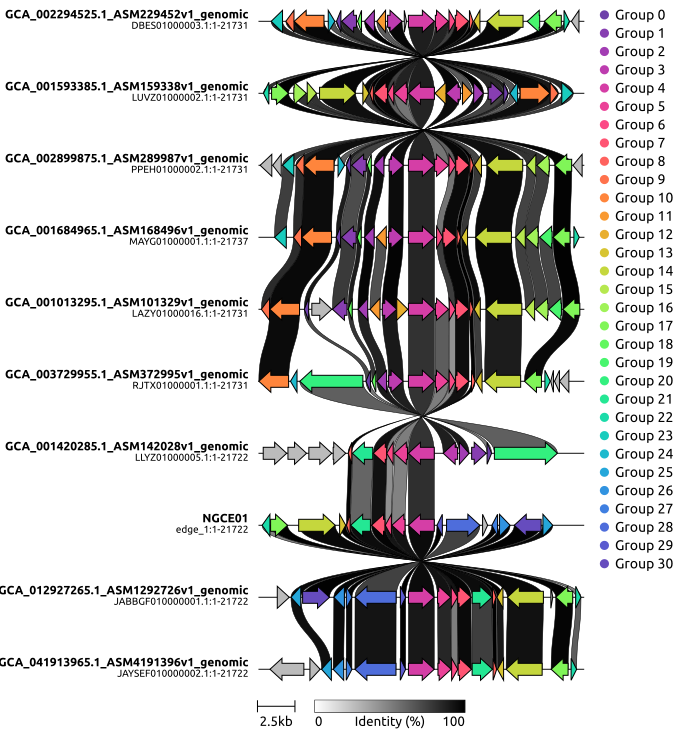


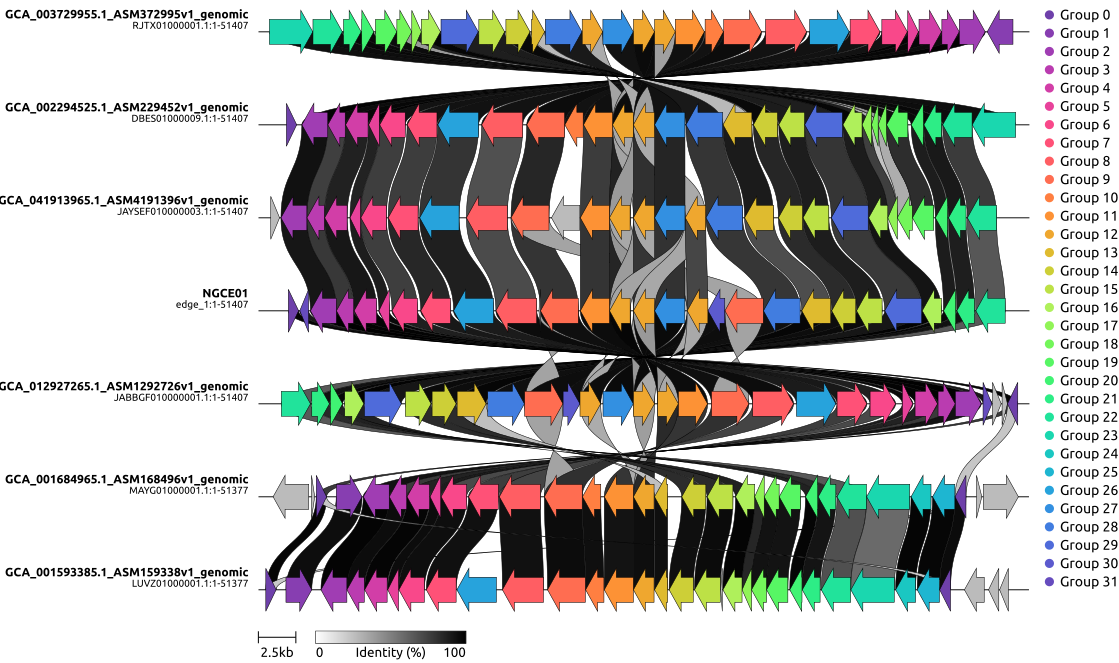
**Supplementary Figure S11. Synteny comparison of the CBM6–GH5 β-glucan/cellulose-like utilization locus across NGCE01 and reference Chryseobacterium genomes.** A tight genomic window centered on the NGCE01 CBM6–GH5 anchor was extracted and compared against best-hit loci in the reference panel using clinker. CDS features are shown as scaled arrows (strand indicated by direction) and colored by clinker orthology group; connecting ribbons indicate homologous relationships, with shading intensity proportional to amino-acid identity (legend). The locus exhibits a conserved backbone among genomes carrying the module, but pronounced variability in accessory gene content and local arrangement across several references, supporting locus-level remodeling around the CBM6–GH5 functional core.

**
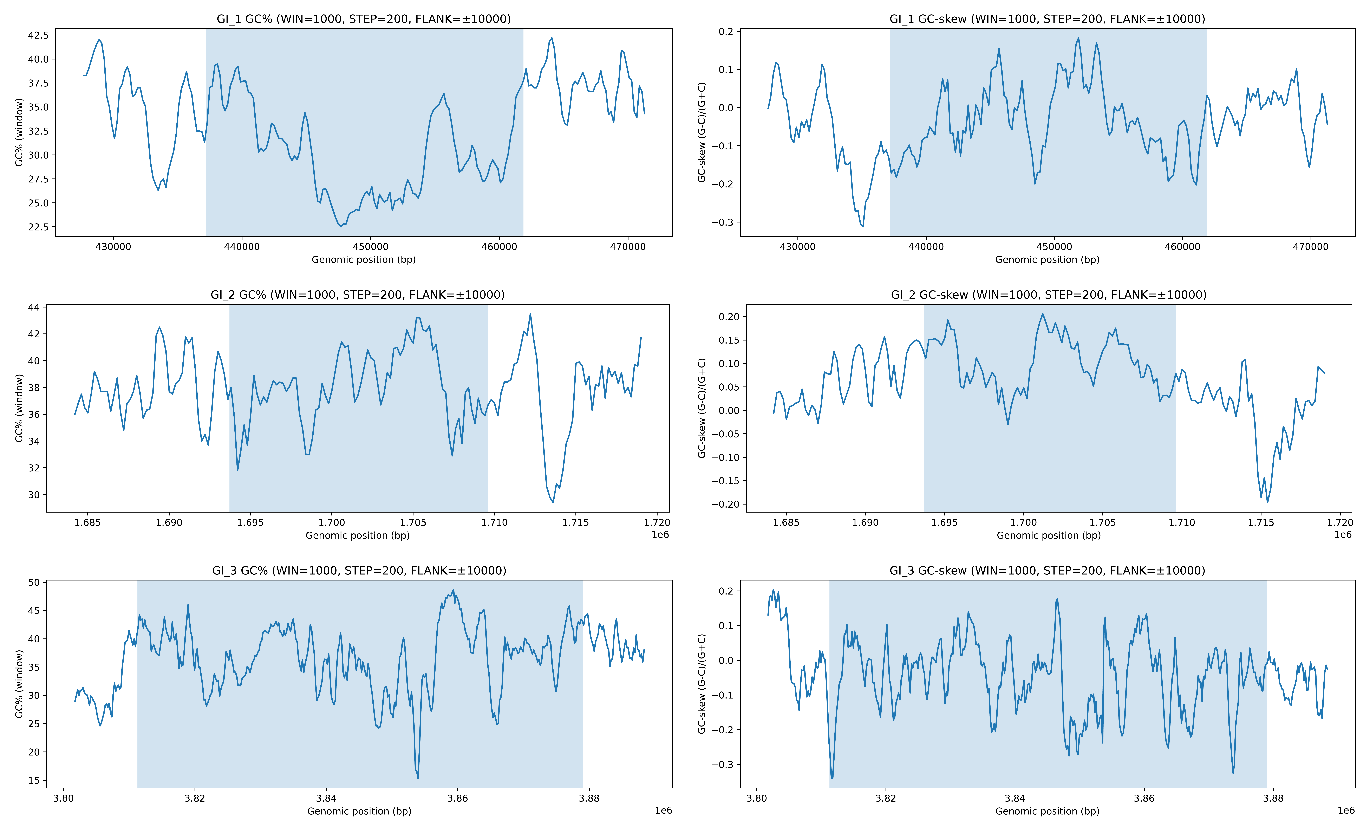
Supplementary Figure S12. Synteny comparison of the GH43-rich hemicellulose-processing “super-block” across NGCE01 and reference Chryseobacterium genomes.** A tight genomic window centered on the NGCE01 GH43 anchor was extracted and compared to best-hit loci across the reference panel using clinker. CDS features are displayed as scaled arrows (orientation indicates strand) and colored by clinker orthology group membership; ribbons connect homologous genes, with ribbon shading reflecting amino-acid identity (legend). Across genomes, the GH43-centered scaffold is broadly conserved but shows substantial locus remodeling via accessory gene turnover and local rearrangements, consistent with modular refactoring of a hemicellulose/arabinoxylan utilization locus rather than uniform conservation of gene content.

**Supplementary Figure 13.** **Sliding-window GC% and GC-skew across genomic islands and flanking regions on edge_1.** For each island (GI_1–GI_3), GC% (left panels) and GC-skew (right panels; (G−C)/(G+C)) were computed in 1 kb windows with 200 bp step across ±10 kb flanks. Shaded regions indicate the island coordinates. GI_1 shows a pronounced GC decrease relative to flanks, whereas GI_2 and GI_3 show more subtle composition shifts.

**
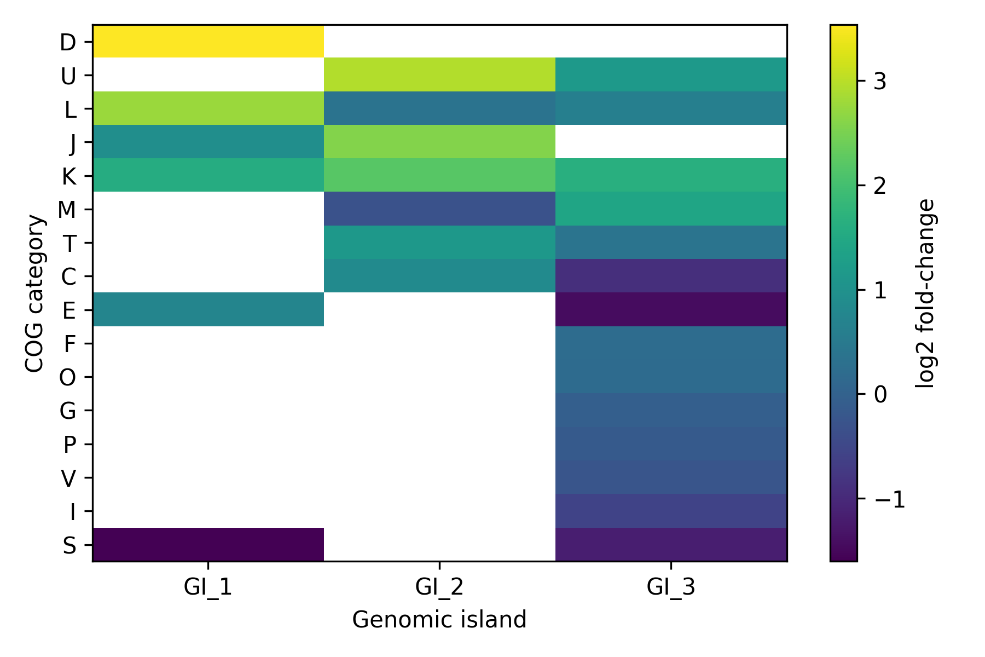
**

**Supplementary Figure S14.** **COG-category enrichment heatmap (log2 fold-change) for GI_1–GI_3 relative to whole-genome background**. Rows denote COG categories and columns denote islands. Values represent log2 fold-change of category proportions among COG-assigned genes in each GI versus the genome background; blank cells indicate zero counts in the GI for that category. GI_1 shows strongest elevation in replication/recombination/repair (L), while GI_3 is elevated in cell envelope biogenesis (M) and transcription (K).

**
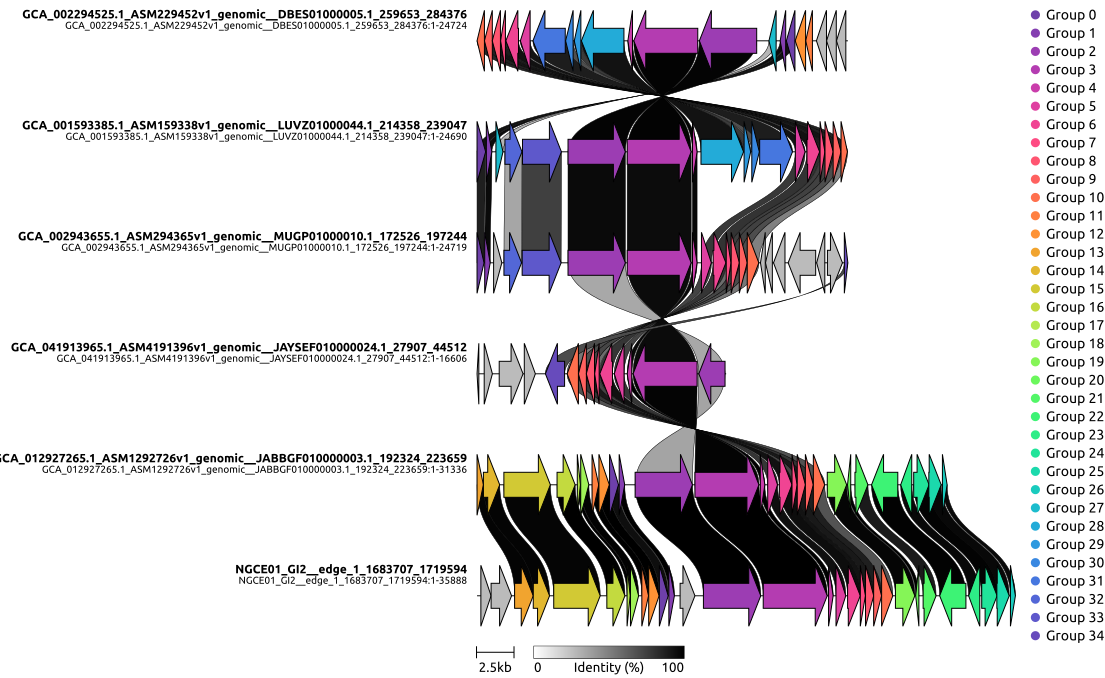
**

**Supplementary Figure S15.** **Gene-synteny conservation of GI_2 across NG-CE01 and five reference genomes.** Clinker alignment of the GI_2 locus (NG-CE01) against the five best-supported reference regions shows broad collinearity and shared gene clusters, consistent with high gene-content similarity (high Jaccard across top matches). Links indicate amino-acid identity between homologous genes; gene arrows indicate orientation.

**
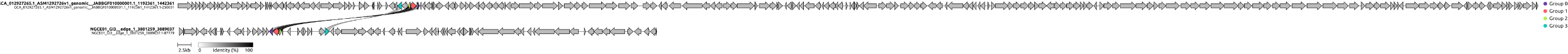
**


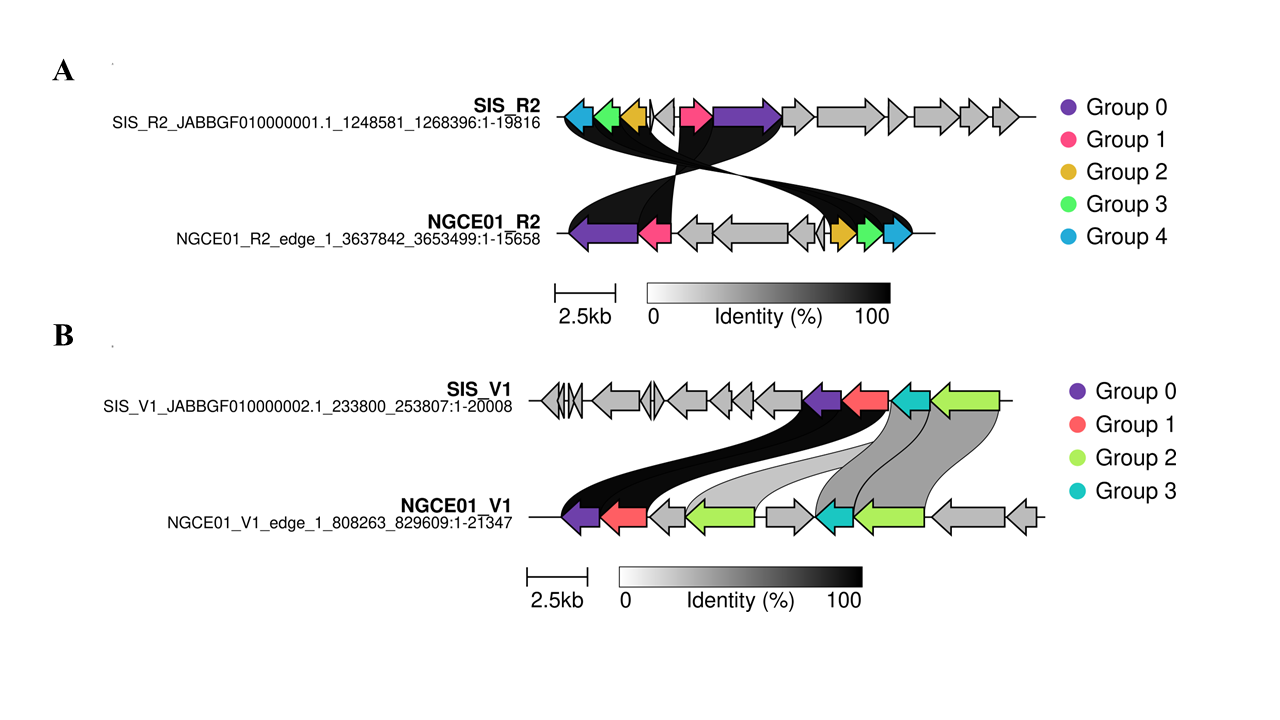
**Supplementary Figure S16. Targeted synteny snapshot for GI_3 against the single best-matching reference genome.** Because GI_3 showed near-zero gene-content similarity to most references (Jaccard = 0 across 12/13 genomes), synteny was visualized against only the highest-sharing reference to avoid empty/degenerate multi-track plots. The alignment highlights limited conserved segments embedded within a largely NG-CE01-specific island structure.

**Supplementary Figure S17. Targeted synteny comparison of representative curated loci in *Chryseobacterium* sp. NG-CE01 versus the nearest sister genome. (A)** R2 intrinsic resistance-like efflux neighborhood (cusC–bepE_4–ttgG) compared between NG-CE01 and *C. cheonjiense* RJ-7-14 (GCA_012927265.1; contig JABBGF010000001.1). **(B)** V1 SusC/SusD/TonB uptake neighborhood compared between NG-CE01 and RJ-7-14 (contig JABBGF010000002.1). Gene arrows denote predicted CDS orientation; shaded connectors indicate homologous segments with nucleotide identity (%) shown by the scale. Panels illustrate conserved locus architecture despite incomplete RBH overlap, consistent with accessory turnover within broadly conserved chromosomal neighborhoods (Supplementary Table S38).
